# Multi-omic longitudinal study reveals immune correlates of clinical course among hospitalized COVID-19 patients

**DOI:** 10.1016/j.xcrm.2023.101079

**Published:** 2023-05-23

**Authors:** Joann Diray-Arce, Slim Fourati, Naresh Doni Jayavelu, Ravi Patel, Cole Maguire, Ana C. Chang, Ravi Dandekar, Jingjing Qi, Brian H. Lee, Patrick van Zalm, Andrew Schroeder, Ernie Chen, Anna Konstorum, Anderson Brito, Jeremy P. Gygi, Alvin Kho, Jing Chen, Shrikant Pawar, Ana Silvia Gonzalez-Reiche, Annmarie Hoch, Carly E. Milliren, James A. Overton, Kerstin Westendorf, IMPACC Network, James Abraham, Michael Adkisson, Marisa Albert, Luz Altamirano Torres, Bonny Alvarenga, Matthew L. Anderson, Evan J. Anderson, Azlann Arnett, Hiromitsu Asashima, Mark A. Atkinson, Lindsey R. Baden, Brenda Barton, Katherine Beach, Elizabeth Beagle, Patrice M. Becker, Matthew R. Bell, Mariana Bernui, Chris Bime, Arun Boddapati Kumar, Leland J. Booth, Brittney Borresen, Scott C. Brakenridge, Laurel Bristow, Robert Bryant, Carolyn S. Calfee, Juan Carreño Manuel, Sidney Carrillo, Suzanna Chak, Iris Chang, Jennifer Connors, Michelle Conway, David B. Corry, David Cowan, Brett Croen, Charles S. Dela Cruz, Gina Cusimano, Lily Eaker, Carolyn Edwards, Lauren I.R. Ehrlich, David Elashoff, Heidi Erickson, David J. Erle, Shelli Farhadian, Keith Farrugia, Benoit Fatou, Andrea Fernandes, Ana Fernandez-Sesma, Gabriela K. Fragiadakis, Sara Furukawa, Janelle N. Geltman, Rajani Ghale, Maria González Carolina Bermúdez, Michael I. Goonewardene, Estella Guerrero Sanchez, Faheem W. Guirgis, David A. Hafler, Sydney Hamilton, Paul Harris, Arash Hayati Nemati, Carolyn M. Hendrickson, Nelson I. Higuita Agudelo, Thomas Hodder, Steven M. Holland, Catherine L. Hough, Christopher Huerta, Kerin C. Hurley, Scott R. Hutton, Akiko Iwasaki, Alejandra Jauregui, Meenakshi Jha, Brandi Johnson, David Joyner, Kirsten N. Kangelaris, Geoffrey Kelly, Zain Khalil, Zenab Khan, Farrah Kheradmand, James N. Kim, Hiroki Kimura, Albert I. Ko, Bernard Kohr, Monica Kraft, Matthew Krummel, Michele F. Kutzler, Jessica Lasky-Su, Serena Lee, Deanna Lee, Michael Leipold, Claudia Lentucci, Carolyn Leroux, Edward Lin, Shanshan Liu, Christina Love, Zhengchun Lu, Lenka Maliskova, Brittany Manning Roth, Monali Manohar, Mark Martens, Grace A. McComsey, Kerry McEnaney, Renee McLin, Esther Melamed, Nataliya Melnyk, Kevin Mendez, William B. Messer, Jordan P. Metcalf, Gregory Michelotti, Eran Mick, Subhasis Mohanty, Jarrod Mosier, Lubbertus C.F. Mulder, Maimouna Murphy, Kari R.C. Nadeau, Ebony Nelson, Allison Nelson, Viet Nguyen, Jordan Oberhaus, Bernadine Panganiban, Kathryn L. Pellegrini, Harry C. Pickering, Debra L. Powell, Scott Presnell, Bali Pulendran, Adeeb H. Rahman, Ahmad Rashid Sadeed, Ariel Raskin, Elaine F. Reed, Susan Ribeiro Pereira, Adreanne M. Rivera, Jacob E. Rogers, Angela Rogers, Brandon Rogowski, Rebecca Rooks, Yael Rosenberg-Hasson, Jessica Rothman, Justin F. Rousseau, Ramin Salehi-Rad, Mehmet Saluvan, Hady Samaha, Joanna Schaenman, Ron Schunk, Nicholas C. Semenza, Subha Sen, Jonathan Sevransky, Vicki Seyfert-Margolis, Tanzia Shaheen, Albert C. Shaw, Scott Sieg, Sarah A.R. Siegel, Natalia Sigal, Nadia Siles, Brent Simmons, Viviana Simon, Gagandeep Singh, Lauren Sinko, Cecilia M. Smith, Kinga K. Smolen, Li-Zhen Song, Komal Srivastava, Peter Sullivan, Caitlin Syphurs, Johnstone Tcheou, George P. Tegos, Greg K. Tharp, Alexandra Tong Ally, Alexandra Tsitsiklis, Ricardo F. Ungaro, Tatyana Vaysman, Arthur Viode, Randi Vita, Xiaomei Wang, Alyssa Ward, Dawn C. Ward, Andrew Willmore, Kyra Woloszczuk, Kari Wong, Prescott G. Woodruff, Leqi Xu, Simon van Haren, Adriana van de Guchte, Yujiao Zhao, Charles B. Cairns, Nadine Rouphael, Steven E. Bosinger, Seunghee Kim-Schulze, Florian Krammer, Lindsey Rosen, Nathan D. Grubaugh, Harm van Bakel, Michael Wilson, Jayant Rajan, Hanno Steen, Walter Eckalbar, Chris Cotsapas, Charles R. Langelier, Ofer Levy, Matthew C. Altman, Holden Maecker, Ruth R. Montgomery, Elias K. Haddad, Rafick P. Sekaly, Denise Esserman, Al Ozonoff, Patrice M. Becker, Alison D. Augustine, Leying Guan, Bjoern Peters, Steven H. Kleinstein

**Affiliations:** 1 Clinical and Data Coordinating Center, Boston Children’s Hospital, Boston, MA 02115, USA; 2 Emory School of Medicine, Atlanta, GA 30322, USA; 3 Benaroya Research Institute, University of Washington, Seattle, WA 98101, USA; 4 University of California San Francisco, San Francisco, CA 94115, USA; 5 The University of Texas at Austin, Austin, TX 78712, USA; 6 Icahn School of Medicine at Mount Sinai, New York, NY 10029, USA; 7 Precision Vaccines Program, Boston Children’s Hospital, Harvard Medical School, Boston, MA 02115, USA; 8 Yale School of Medicine, New Haven, CT 06510, USA; 9 Yale School of Public Health, New Haven, CT 06510, USA; 10 Knocean, Inc., Toronto, ON M6P 2T3, Canada; 11 La Jolla Institute for Immunology, La Jolla, CA 92037, USA; 12 Drexel University, Tower Health Hospital, Philadelphia, PA 19104, USA; 13 National Institute of Allergy and Infectious Diseases, National Institute of Health, Bethesda, MD 20814, USA; 14 Stanford University School of Medicine, Palo Alto, CA 94305, USA; 15 Broad Institute of MIT & Harvard, Cambridge, MA 02142, USA

**Keywords:** COVID-19, SARS-CoV-2, multi-omics, systems immunology, immunophenotyping, longitudinal modeling

## Abstract

The IMPACC cohort, composed of >1,000 hospitalized COVID-19 participants, contains five illness trajectory groups (TGs) during acute infection (first 28 days), ranging from milder (TG1–3) to more severe disease course (TG4) and death (TG5). Here, we report deep immunophenotyping, profiling of >15,000 longitudinal blood and nasal samples from 540 participants of the IMPACC cohort, using 14 distinct assays. These unbiased analyses identify cellular and molecular signatures present within 72 h of hospital admission that distinguish moderate from severe and fatal COVID-19 disease. Importantly, cellular and molecular states also distinguish participants with more severe disease that recover or stabilize within 28 days from those that progress to fatal outcomes (TG4 vs. TG5). Furthermore, our longitudinal design reveals that these biologic states display distinct temporal patterns associated with clinical outcomes. Characterizing host immune responses in relation to heterogeneity in disease course may inform clinical prognosis and opportunities for intervention.

## Highlights

• Distinct baseline and temporal patterns are associated with the clinical course
• Persistent viral levels, despite high antibody titers, are associated with severity
• Severity is linked to reduced cytotoxic NK cells, increased inflammation, and thrombosis
• Myocardial damage markers distinguish critical patients who recover from those who die

## Graphical abstract

**Figure:**
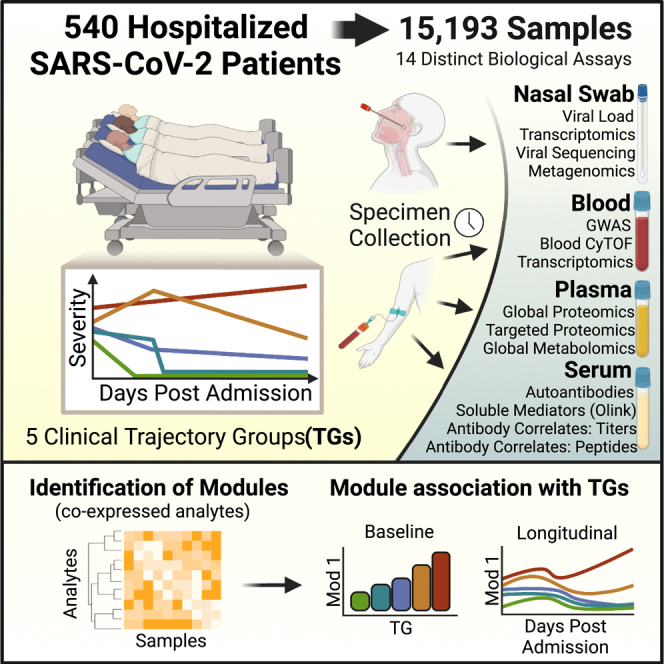

## Teaser

Diray-Arce et al. conduct deep immunophenotyping of acute COVID-19 infection using more than 15,000 longitudinal samples from 540 hospitalized patients in the IMPACC cohort. The study comprehensively defines baseline and longitudinal immunologic states that are associated with mild to fatal disease trajectory groups.

## Introduction

Throughout the COVID-19 pandemic, scientists worldwide have characterized immune responses and host-pathogen interactions to severe acute respiratory syndrome coronavirus 2 (SARS-CoV-2) infection to gain insight into disease pathogenesis and identify potential interventions for COVID-19. Studies assessing distinct elements of viral variants and cellular and humoral immunity from different participant populations have greatly improved our understanding of SARS-CoV-2 pathogenesis.^1^^,^^2^^,^^3^^,^^4^^,^^5^^,^^6^ However, to design and deploy precision prognostics and therapeutics, it is essential to address the heterogeneity in clinical outcomes of COVID-19 and precisely define correlates of host immune responses to that heterogeneity.

The clinical manifestations of COVID-19 are diverse, ranging from asymptomatic disease to hospitalization and death.^7^^,^^8^^,^^9^ Even among hospitalized patients, who are at the highest risk for death, clinical courses are highly variable. To provide a comprehensive and unbiased study of the clinical course, immunology, virology, and genetics of acute COVID-19, we established a geographically diverse US consortium of 15 centers and 20 hospital recruitment sites (Immunophenotyping Assessment in a COVID-19 Cohort, or IMPACC).^10^ IMPACC analyzed participant characteristics to capture the dynamics of clinical course and defined five disease course trajectories spanning rapid recovery through fatal outcomes.^11^

Here, we carried out deep immunophenotyping of 15,193 longitudinal samples from 540 IMPACC adult participants with a confirmed positive SARS-CoV-2 PCR over the first 28 days after hospital admission. To define the immune status of the study participants, we employed six core immunophenotyping approaches on blood samples: serology (anti-SARS-CoV-2-specific and anti-interferon [IFN] antibodies), proteomics (circulating markers from serum and plasma reflecting immune status from protein states), metabolomics (metabolites and lipids), CyTOF (leukocyte frequency and phenotype), gene expression (host bulk RNA sequencing [RNA-seq] and metagenomics), and genomics (DNA sequence, genome-wide association study [GWAS]). In addition, we analyzed the nasal epithelium, the port of entry of SARS-CoV-2 infection, for viral load and viral sequences and host transcriptomic profiles. Overall, we identified biologic states associated with the five COVID-19 disease trajectory groups defined by IMPACC, revealing potential determinants of clinical heterogeneity and potential actionable targets for prognostic biomarkers and therapeutic intervention.

## Results

### Immunophenotyping of participants within five clinical trajectory groups

**Figure 1:**
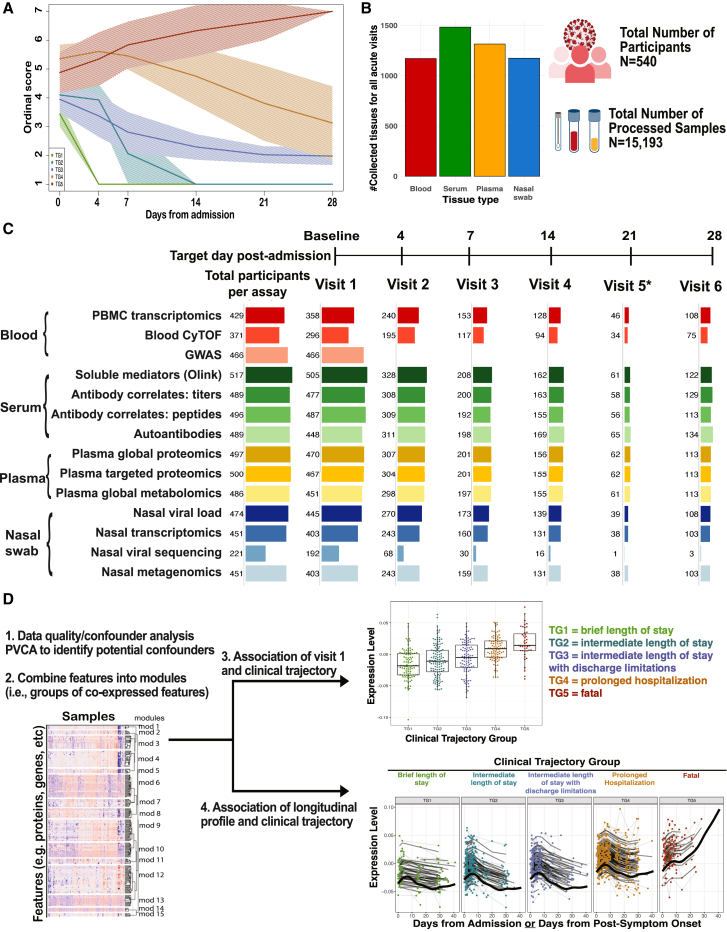
Overview of IMPACC cohort, sample collection, and immunophenotyping (A) Clinical trajectory group assignment of IMPACC cohort participants (N = 1,164).^10^^,^^11^^,^^12^ (B) The total number of collected tissues (whole blood, serum, plasma, and nasal swab samples) for all acute visits (up to day 28 post-admission, including escalation visits). A total of 15,193 samples were profiled from 540 participants across 20 hospital recruitment sites from 15 biomedical centers. (C) The total number of participants profiled by 14 different immunophenotyping assays over the course of the scheduled acute visits (visits 1–6). (D) Data analysis involved a rigorous data quality and confounder analysis, dimensionality reduction to combine features into modules, and association of module levels at visit 1 and their longitudinal pattern with the clinical trajectory group. Expression levels of modules at visit 1 are depicted as boxplots, while longitudinal patterns are shown as line graphs.

We carried out deep immunophenotyping on longitudinal data and samples over the initial 28 days post-hospital admission of 540 adult participants with PCR-confirmed SARS-CoV-2 infection enrolled in the IMPACC cohort between May 6, 2020 and December 9, 2020 (Table S1). Five illness trajectory groups were identified previously^11^ using clinical data from the entire IMPACC cohort (1,164 participants) and latent class modeling of longitudinal observation of a modified ordinal score,^12^ reflecting both the degree of respiratory support required and the presence or absence of activity limitations or oxygen requirement at discharge.^11^ The model classified each participant into one of five groups: trajectory group 1 (TG1; n = 119) was characterized by relatively mild respiratory disease and a brief hospital stay (median [interquartile range (IQR)] 3 [2] days) with no limitations at hospital discharge; TG2 (n = 149) generally required more respiratory support than TG1 and had a longer length of hospital stay (LOS) (median [IQR] 7 [4] days) but no limitations at discharge; and TG3 (n = 110) was characterized by roughly similar respiratory support requirements and LOS (median [IQR] 7 [7] days) as TG2 but generally had limitations at discharge. Two additional groups had overall higher respiratory support requirements during their hospital stay: TG4 (n = 106) generally received more aggressive respiratory support and experienced a prolonged LOS (median [IQR] 20 [12] days), and TG5 (n = 56) was characterized by high respiratory support requirements and fatal illness by day 28 (Figure 1A). Detailed clinical characteristics for the entire IMPACC cohort based on TG assignment have been previously reported.^11^ Participant demographics, comorbidities, time from symptom onset to hospitalization, and baseline clinical respiratory status, radiographic findings, and clinical laboratory data for the 540 participants with deep immunophenotyping data analyzed here reflect characteristics of the entire IMPACC cohort (Table S1).

Clinical laboratory values were collected both at baseline and at scheduled visits during the hospital stay if ordered by the clinical care team.^10^ Longitudinal multi-omics profiles were generated for each participant employing 14 distinct assays on blood and nasal swab samples at each visit. In total, 15,193 biological samples were processed and analyzed from 540 participants (Figure 1B; Table S1). These assays included nasal viral load and sequence, serology, blood cytometry, plasma proteomics, serum cytokine/chemokine, plasma metabolomics, nasal and peripheral blood mononuclear cell (PBMC) transcriptomics, nasal metagenomics, and genetics (Figure 1C).

### A common analytic framework to identify associations with clinical severity

We developed a common analytic framework for all assays (Figure 1D; STAR Methods). Briefly, this framework included a dimensionality reduction step followed by mixed-effects modeling for association with the five clinical trajectory groups, with confounding effects properly adjusted in this process. For assay readouts with >50 features, we identified correlated feature modules (referred to here as “modules”) using weighted gene co-expression network analysis (WGCNA).^13^ For a given module in an assay, we define the module values across samples as the first principal component constructed using features included in this module. We investigated the behavior of each feature (or module) both at visit 1 (within 72 h of hospital admission) and longitudinally (up to 28 days post-hospital admission) and correlated it with clinical outcomes. More specifically, we tested both if a feature exhibited a monotonic trend from the mildest (TG1) to most severe (TG5) disease course at visit 1 using mixed-effect ordinal regression (clmm) and if a feature showed differential kinetics over the whole time course (visits 1–6) via a generalized additive model with mixed effects (gamm4) where we examined if the average (referred to as intercept in the gamm4 documentation) or shape (referred to as the smoothing term in the gamm4 documentation) differs across the clinical trajectory groups. Features with a false discovery rate (FDR) <5% were considered significant based on the adjusted p value (referred to as adj.p).^14^ For both analyses, significant features were further tested for differences between each pair of TGs to facilitate interpretation.^15^^,^^16^

### Viral loads and antibody responses associated with disease trajectory

Viral loads and antibody responses are key aspects of host-pathogen interactions that relate to disease severity.^17^^,^^18^^,^^19^ We assessed nasopharyngeal viral loads by RT-PCR, viral variants by whole-genome sequencing, anti-receptor binding domain (RBD) and anti-spike immunoglobulin G (IgG) antibodies by ELISA, and antibodies targeting the entire SARS-CoV-2 linear peptidome by programmable phage display.^20^

**Figure 2:**
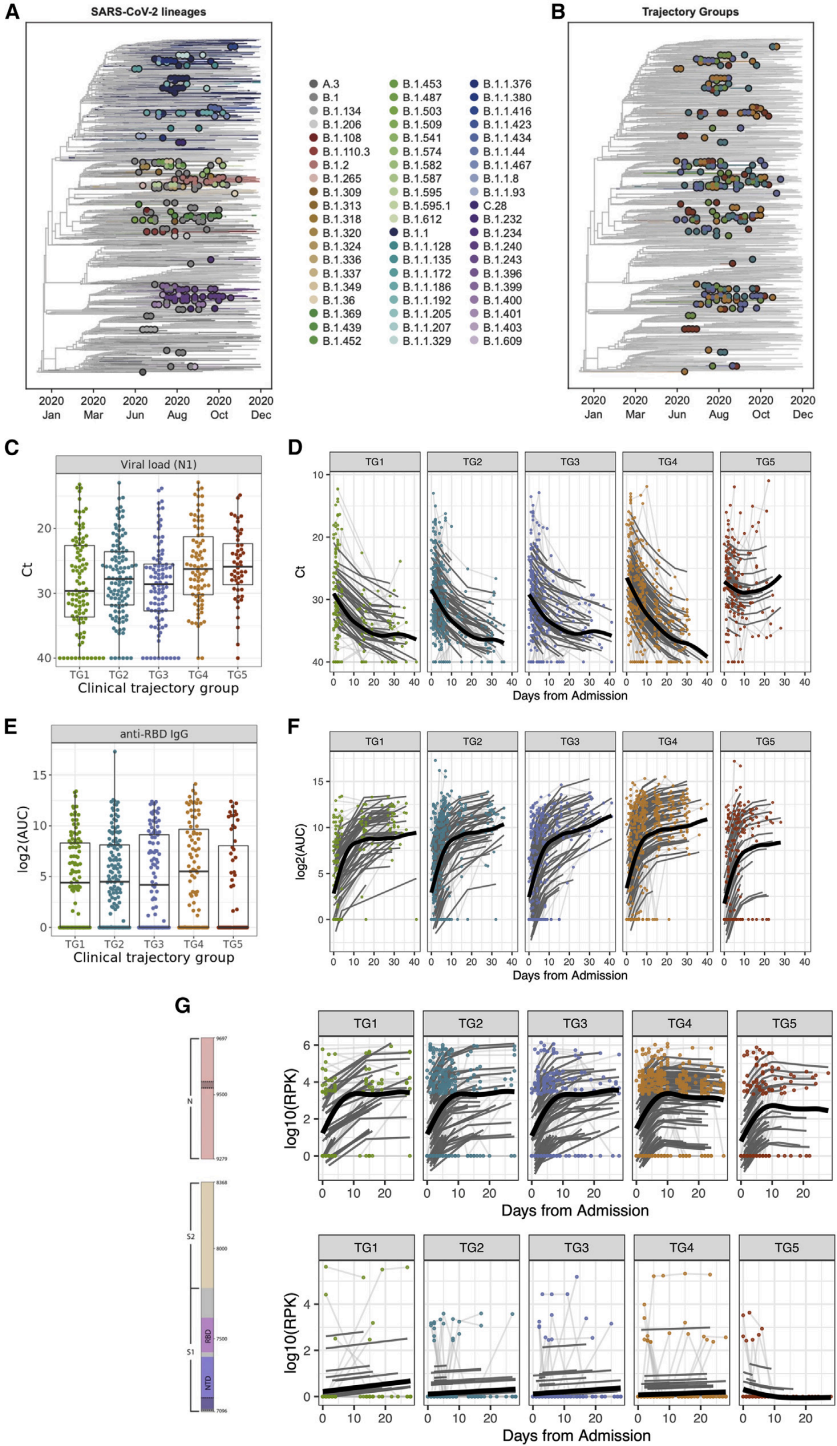
SARS-CoV-2 viral loads and antibody responses were associated with clinical trajectory group (A) Viral sequencing identified 60 PANGO lineages across the cohort. (B) The clinical trajectory group was not associated with any of the 9 lineages detected. (C) Viral loads (SARS-CoV-2 N1 gene Ct values) measured from samples collected at visit 1 (significantly higher in participants with more severe disease [adj.p = 0.037]). For each boxplot, the vertical line indicates the median, the box indicates the interquartile range, and the whiskers indicate 1.5 times the interquartile range. (D) Viral loads (SARS-CoV-2 N1 gene Ct values) from samples collected during the acute visits (shape: adj.p = 0.001, average: adj.p = 1.68e−5). (E) Anti-RBD IgG area under the curve (AUC) values measured from samples collected at visit 1 (lower in TG5 [adj.p = 0.68]). For each boxplot, the vertical line indicates the median, the box indicates the interquartile range, and the whiskers indicate 1.5 times the interquartile range. (F) Anti-RBD IgG AUC values from samples collected during the acute visits (shape: adj.p = 0.07, average: adj.p = 0.3). (G) Seroreactivity (log10 summed RPK across SARS-CoV-2 regions) across samples collected from the acute visits were measured longitudinally in two distinct regions (highlighted in gray within spike and N annotations): decreased seroreactivity in the NTD (shape: adj.p = 6.78e−6, average: adj.p = 0.058) and decreased overall seroreactivity in the LINK domain of the nucleoprotein (shape: adj.p = 0.023). (C and D) Because lower Ct values indicate higher viral loads, the y axis is reversed. (C and E) Shown are median values (horizontal lines), IQRs (boxes), and 1.5 IQRs (whiskers), as well as all individual points.

Whole-genome viral amplification generated complete viral genomes from 316 nasopharyngeal swab samples collected from 221 participants. Genotyping identified 60 lineages from Phylogenetic Assignment of Named Global Outbreak^21^^,^^22^ (PANGO) across the cohort (Figure 2A). All viral genomes were of the Wuhan strain. No variants of interest or concern, such as Delta or Omicron variants, were detected as the samples were collected prior to the occurrence of these variants. Clinical trajectory group was not associated with any of the 9 lineages that were detected across at least 3 recruitment sites or with participant-specific mutations (Figure 2B).

SARS-CoV-2 viral loads were measured on 1,174 nasopharyngeal swab samples collected from 474 participants. We did not detect associations between viral load and sex, age, enrollment site, and other sample metadata and demographic variables. However, the RT-PCR median cycle threshold (Ct) values for both SARS-CoV-2 nucleocapsid protein genes N1 and N2 differed significantly among the five clinical trajectory groups at hospital admission (visit 1) (N1 Ct, adj.p = 0.04, and N2 Ct, adj.p = 0.04; Figures 2C and S1A). The median viral loads were lowest (higher Ct values) in the participant group with mildest disease (TG1) and highest in the group with most severe disease (TG5). Longitudinal analysis identified additional significant differences in the shape of the viral loads across time (N1 Ct, adj.p = 0.001, and N2 Ct, adj.p = 0.0003, Figures 2D and S1B). While a decline in viral loads was observed for all of the trajectory groups, viral loads in participants with the most severe disease (TG5) plateaued after the first week of hospitalization at a Ct value still <30, suggesting persistent viral RNA throughout the 28 days (Figure 2D).

Antibody titers against SARS-CoV-2 RBD IgG and spike IgG were measured in 1,335 serum samples collected from 489 participants. Anti-RBD and anti-spike IgG values at visit 1 were quantitatively lowest in participants with the most severe disease (TG5), but no significant difference was detected among the five clinical trajectory groups (anti-RBD IgG, adj.p = 0.68, and anti-spike IgG, adj.p = 0.68; Figures 2E and S1C). In contrast, the average (anti-spike IgG, adj.p = 0.07) and shape (anti-spike IgG, adj.p = 0.01, and anti-RBD IgG, adj.p = 0.07; Figures 2F and S1D) of the longitudinal responses were different across the trajectory groups, with TG4 showing the highest values of anti-spike and anti-RBD IgG.

Proteome-wide, linear peptide SARS-CoV-2 (and other human coronaviruses [CoVs]) antibody profiling with VirScan^23^ (i.e., serum phage immunoprecipitation sequencing [PhIP-seq]) was performed on 1,312 serum samples from 496 participants. No batch effects were observed using principal-component analysis (PCA) (Figure S2A) and principal variance component analysis (PVCA) (Figure S2B). Visit 1 pan-SARS-CoV-2 antibody profiles (Figure S2C) did not show any significant association between clinical trajectory group and seroreactivity to any viral protein or region. Focusing on seroreactivity to the spike (S) protein and nucleoprotein (N), the longitudinal analysis identified 323 significant 20 amino acid (aa) windows that were significantly associated with clinical trajectory groups and that mapped to 8 antigenic regions (Figures 2G and S2D; Table S2). In addition, baseline cross-reactivity to human seasonal CoVs did not correlate with the trajectory group (Figure S2E). Most notably, more severe disease (TG5) was associated with increased seroreactivity to the N-terminal domain (NTD) of S and decreased antibody seroreactivity to the LINK domain of N (adj.p = 0.023) (Figure S2D).

Serum samples from 489 participants were screened for autoantibodies against type I IFNs (α, β, and ω) that may enhance susceptibility to severe SARS-CoV-2 infection.^2^ A higher percentage of individuals with more severe disease (TG4, 9.6%, and TG5, 7.8%) had functional blocking anti-IFN antibodies than seen in mild disease (<5% for each of TG1–3; Table S3; p = 0.001). Overall, these data show that viral loads along with anti-SARS-CoV-2 and anti-IFN antibody levels (all lowest in TG1) are significantly associated with clinical disease trajectory, suggesting an important role for antibodies in the host responses and clinical outcomes.

### Analysis of serum and plasma proteomics identifies modules related to natural killer (NK) cells and coagulation pathways associated with severe disease

Soluble proteins are key effectors of immunity in blood. Cytokines, chemokines, and secreted receptors mediate a fast response and short-lived signaling, leading to slower but also longer-lasting changes in plasma protein abundances. Two complementary methodologies were used to investigate the link between protein markers and the clinical trajectory groups. A Proximity Extension Assay (PEA)-based technology (Olink) was used to quantify 92 inflammatory cytokines, chemokines, and soluble receptors in serum; liquid chromatography/mass spectrometry (LC/MS) was used to monitor 241 selected classical plasma proteins in a targeted fashion and 508 plasma proteins that were detected and quantified in discovery mode. The rationale for this 3-pronged approach was to increase the coverage of the serum/plasma proteome by using dedicated workflows for chemokines, cytokines, and secreted receptors and two different fractions of the plasma proteome before and after depletion of the most abundant plasma proteins.^24^

### Olink-link based cytokine, chemokine, and secreted receptor analysis

Olink was generated and analyzed on 1,386 serum samples from 517 participants. The Olink assay detects and quantifies cytokines, chemokines, and secreted receptors (for brevity, all referred to as “soluble proteins”). No batch effect was observed using PCA (Figure S3A) and PVCA (Figure S3B). WGCNA identified six modules ranging from 6 to 30 soluble proteins (Figure S3C; Table S4). The ImmuneXpresso^25^ database, associating cytokines, chemokines, and secreted receptors to their action on immune cells, was used to label the six Olink modules. At visit 1, five of these modules were significantly associated with clinical outcome (Table S4).

**Figure 3:**
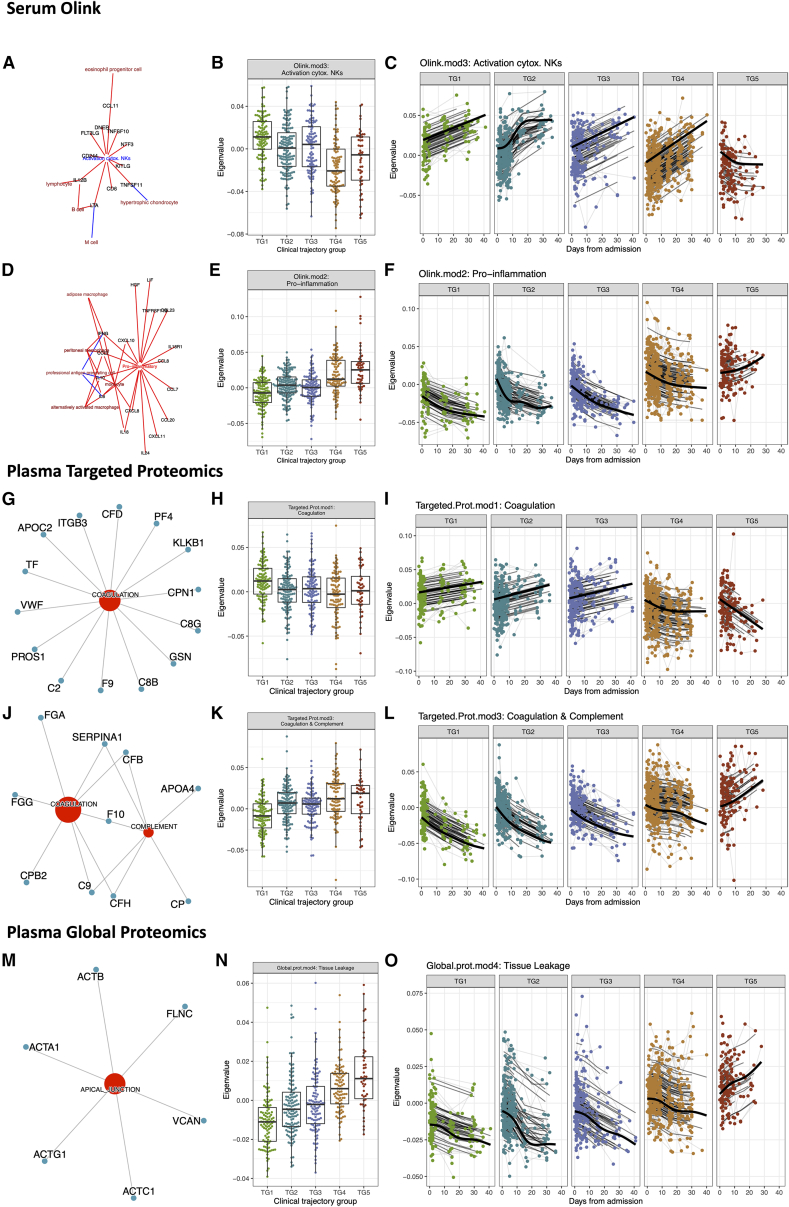
Association of serum proximity extension assay (Olink) and plasma proteomics modules with clinical trajectory groups (A–F) Analysis of serum Olink data identified significant associations in the expression levels of (A–C) Olink.mod3 and (D–F) Olink.mod2 among clinical trajectory groups. ImmuneXpresso,^25^ a text-mining tool linking cytokines/chemokines to cells, was used to annotate (A) Olink.mod3 (activator of cytotoxic NKs) and (D) Olink.mod2 (pro-inflammatory cytokines). (A and D) Significant enrichments (i.e., Fisher’s exact test p ≤ 0.05) are presented in the network. Blue arrows correspond to negative correlation/repression, while red arrows indicate positive correlation/production/activation. (B and C) Levels of Olink.mod3 (B) at visit 1 and (C) over time. (E and F) Levels of Olink.mod2 (E) at visit 1 and (F) over time. (G–O) Analysis of targeted and global mass spectrometry-based plasma proteomics data identified significant associations of (G–I) Targeted.Prot.mod1, (J–L) Targeted.Prot.mod3, and (M–O) Global.prot.mod4 with the clinical trajectory group. (G) MSigDB hallmark pathway analysis of the 58 proteins of Targeted.Prot.mod1 identified an association with coagulation. (H and I) Levels of Targeted.Prot.mod1 (H) at visit 1 and (I) over time. (J) MSigDB hallmark pathway analysis of the 26 proteins of Targeted.Prot.mod3 identified an association with coagulation and complement hallmark gene sets. (K and L) Levels of Targeted.Prot.mod3 at (K) visit 1 and (L) over time. Analysis of global mass spectrometry-based plasma proteomics data identified significant associations of Global.prot.mod4 with the clinical trajectory group. (M) MSigDB hallmark pathway analysis of the 54 proteins of Global.prot.mod4 identified an association with apical junctions, myogenesis, and epithelial mesenchymal transition. (N and O) Levels of Global.prot.mod4 at (N) visit 1 and (O) over time. (B, E, H, K, and N) For each boxplot, the vertical line indicates the median, the box indicates the interquartile range, and the whiskers indicate 1.5 times the interquartile range. (B, C, E, F, H, I, K, L, N, and O) Each point is a sample from an individual participant. Light gray lines connect samples from the same participant. Thick black lines correspond to a smooth spline fit for all participants in each trajectory group.

One of these five modules (Olink.mod3, annotated as “activators of NKs”) was higher in participants who recovered relatively quickly (TG1–3) (adj.p = 8.85e−11). This module, composed of 11 soluble proteins, was enriched for features related to activating cytotoxic NK cells and included molecules such as CD244 and interleukin 12B (IL-12B) (Figure 3A). Six out of the 11 proteins were annotated to “activator of lymphocytes” based on ImmuneXpresso^25^ and a literature search.^26^^,^^27^^,^^28^^,^^29^ This module had an increased expression in milder trajectory groups, suggesting a role in disease recovery (Figure 3B). Consistent with this hypothesis, the expression of this module increased across time in groups TG1 through TG4, but not in the fatal trajectory group (TG5), where the opposite trend was observed (shape: adj.p = 4.44e−12, average: adj.p = 3.39e−18; Figure 3C). Notably, participants in TG4 that presented with severe disease but survived past day 28 started with lower levels of “activators of NKs” but exhibited an increase of those markers over time to levels comparable to TG1–3. In contrast, four modules (Olink.mod1 [adj.p = 4.01e−6] annotated as “cytokines produced by neutrophils” including the cytokine tumor necrosis factor [TNF] and IL-17A; Olink.mod2 [adj.p = 3.55e−18] annotated as “pro-inflammatory”; Olink.mod4 [adj.p = 4.08e−4] including the ADA deaminase; and Olink.mod6 [adj.p = 2.38e−5] annotated as “activators of macrophages”) were higher at visit 1 in participants with a more severe disease course (Table S4). The most significant module, Olink.mod2, was composed of pro-inflammatory cytokines and chemokines, including IL-6, CXCL-8 (IL-8), and CXCL-10 (IP10) (Figure 3D) (13/17 proteins) annotated as “produced by monocytes.”^30^^,^^31^^,^^32^ Baseline (Figure 3E) and longitudinal analyses revealed that this pro-inflammatory module persisted at elevated levels in participants that ultimately died (TG5), while it decreased over time in participants in the other trajectory groups (TG1–4) (shape adj.p = 4.70e−10, average adj.p = 4.40e−42; Figure 3F). In addition, cytokines in the pro-inflammatory modules were directly induced by SARS-CoV-2 infection (Figure S3D). Overall, these results identified early cytokines and chemokines as well as an NK cell link that are associated with clinical trajectories that distinguish fatal from non-fatal disease.

### Targeted mass spectrometry-based classical plasma proteomics analysis

In total, 1,302 plasma samples from 500 participants were subjected to a targeted LC/MS-based proteomics assay. Using the multiple reaction monitoring (MRM) data acquisition mode, we tracked 241 classical plasma proteins, many of which have immune modulatory roles and thus are important for a more complete molecular immunophenotyping. PVCA (Figure S4A) and PCA (Figures S4B and S4C) indicated batch effects based on the two phases in which the samples were processed and analyzed, which were corrected for using the ComBat algorithm.^33^ For this targeted dataset of classical plasma proteins, WGCNA resulted in 7 modules ranging in size from 16 to 62 proteins. Two of the seven modules (Targeted.Prot.mod1 and Targeted.Prot.mod3) showed significant differences across the five clinical trajectory groups (Figure S4D).

Targeted.Prot.mod1 was enriched for proteins annotated to the “coagulation” pathway (14/62 proteins), including the fibrinolysis stimulator plasma kallikrein (KLKB1) (Figure 3G). Its expression differed significantly between TGs at visit 1 (adj.p = 2.19e−3; Figure 3H) and longitudinally (shape adj.p = 3.06e−14, average adj.p = 1.61e−14; Figure 3I). Participants in the mild to moderate clinical trajectory groups (TG1–3) started out with increased levels of Targeted.Prot.mod1 relative to participants from the more severe trajectory groups (TG4–5). In addition to starting at higher levels (Figure 3H), participants in TG1–3 showed a steady increase in their abundance levels over time (Figure 3I). In contrast, participants in TG4–5 showed a clear downward pattern during their hospital stays, highlighting the prognostic nature associated with the dynamics and directionality of the proteins in Targeted.Prot.mod1. Interestingly, participants that ultimately died (TG5) continued to show a downward trend, while the expression leveled off after 10–15 days in severely ill participants who eventually recovered or stabilized (TG4) (Figure 3I; Table S5).

Targeted.Prot.mod3 also contained proteins associated with the “coagulation” pathway (9/33 proteins) including the fibrinolysis inhibitor carboxypeptidase B2 (CPB2) in addition to proteins from the complement pathway (8/33 proteins) (Figure 3J). Like Targeted.Prot.mod1, the expression of Targeted.Prot.mod3 also differed significantly between TGs at visit 1 (adj.p = 1.32e−7; Figure 3K) and longitudinally (shape adj.p = 5.52e−15, average adj.p = 8.90e−25; Figure 3L). However, the abundance levels of the proteins in Targeted.Prot.mod3 at visit 1 and their dynamics were the opposite of those observed in Targeted.Prot.mod1, i.e., lower levels were associated with less severe disease manifestations and faster recovery (TG1–3) (Figure 3K). The lower abundances at visit 1 were followed by a steady decrease in their abundance in plasma over time (Figure 3L; Table S5). In contrast, the plasma concentrations from participants in TG5 showed a steep increase over time, demonstrating the importance of trajectory analyses to leverage the full prognostic value of plasma proteins. The plasma from participants in TG4 showed an intermediate trajectory: an elevated level near the time of hospital admission (visit 1) was followed by a slight decrease. However, concentrations diminish about 3 weeks after hospitalization, consistent with the delayed recovery of these severely ill COVID-19 participants (Figure 3L). Longitudinal analysis of the proteins associated with Targeted.Prot.mod1 and Targeted.Prot.mod3 showed opposing temporal trajectories for the different clinical trajectory groups as one would expect for stimulators and inhibitors of the same biological process such as fibrinolysis.

### Global mass spectrometry-based plasma proteome analysis

To increase the depth of the plasma proteome, we biochemically depleted the most abundant plasma proteins from 1,309 plasma samples associated with 497 participants. The depleted plasma samples were trypsinized and analyzed using LC/MS-based shotgun proteomics (henceforth called “global” proteomics). We did not observe any batch effect using PVCA (Figure S5A) and PCA (Figure S5B) in this global proteomics dataset. We identified 2,109 proteins in total, 508 of which were present in at least 50% of the samples. WGCNA of the expression levels of these 508 proteins identified seven modules ranging in size from 23 to 89 proteins. With the exceptions of Global.prot.mod2 and Global.prot.mod7, the other five modules were significantly associated with clinical trajectory groups at visit 1 (Figure S5C).

The 27 proteins associated with Global.prot.mod4 were enriched in proteins associated with apical junctions (6/27 proteins), including myosins of cardiac (MYH7) as well as musculoskeletal origin (MYH1) (Figure 3M). Interestingly, the majority of the proteins in this module were exclusively observed after biochemical depletion of the most abundant proteins. Without such depletion of the most abundant plasma proteins, the proteins in Global.prot.mod4 would not be observable using the same analytical instrumentation.

Participants with mild to moderate disease course (TG1–3) started out with significantly lower levels of Global.prot.mod4 than the participants with more severe disease trajectories (TG4–5) (adj.p = 2.68e−19, Figure 3N; Table S6). In addition, participants in TG1–3 showed a clear downward trend during recovery, i.e., further reduction of these markers for cardiac injury. This longitudinal pattern of Global.prot.mod4 proteins differentiated severely ill participants that eventually recovered (TG4), who shared a downward trend, from those with fatal outcomes (TG5), who had a continuous upward trend (shape adj.p = 4.53e−11; Figure 3O; Table S6). These trends suggest significant involvement and damage of heart and lung in the acute phase of the disease. Worsening myocardial injury is associated with increased epithelial damage, as indicated by markers of apical junction damage^34^ and epithelial-mesenchymal transition.^35^ This is consistent with the higher cardiac troponin levels, associated with myocardial injury, previously observed for the participants in the most severe trajectory group in the IMPACC cohort.^11^

### Plasma global metabolomics reveals metabolic dysregulation in hospitalized participants

**Figure 4:**
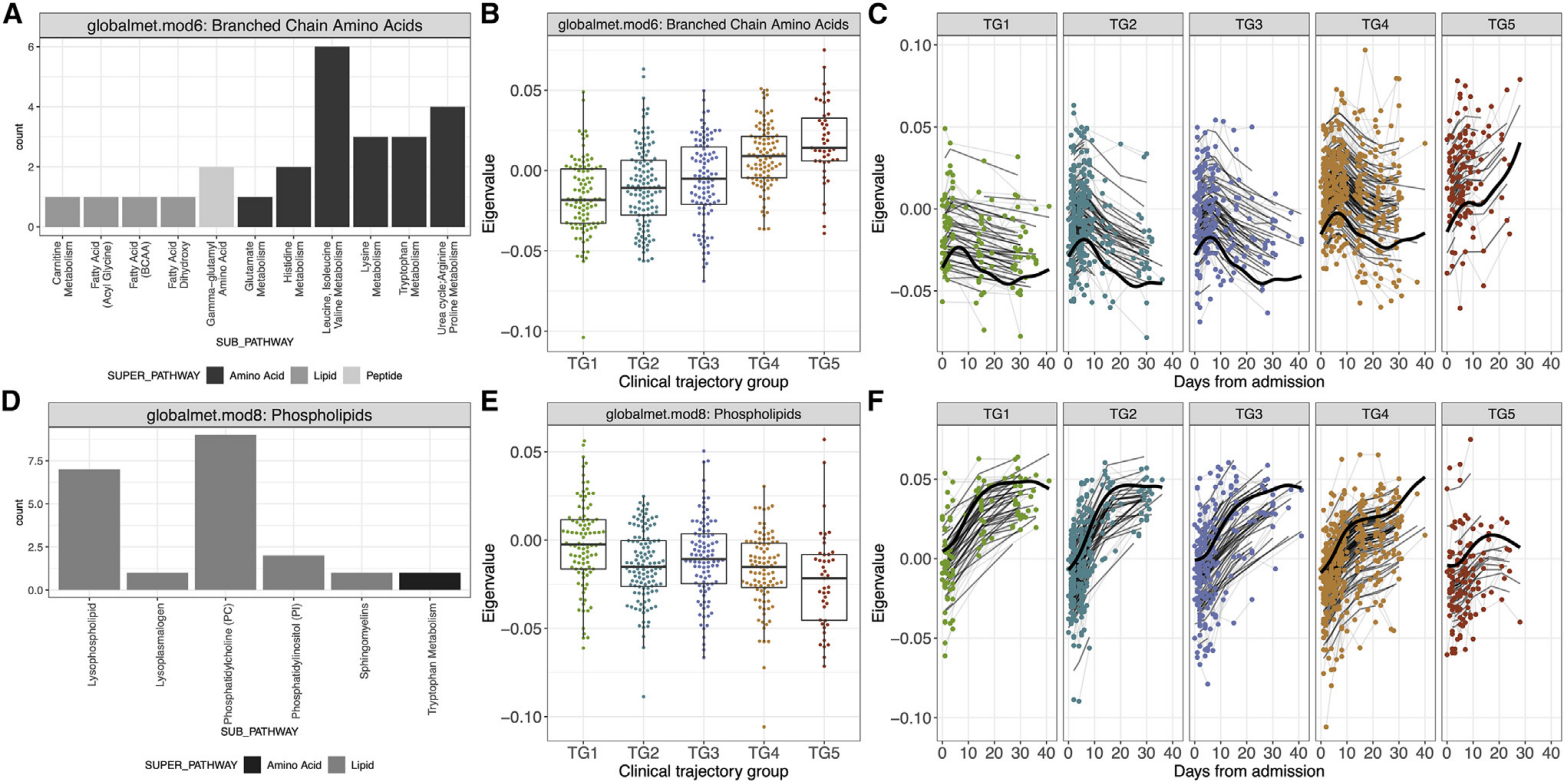
Association of plasma metabolomics modules with clinical trajectory groups (A–F) Analysis of plasma metabolomics data identified significant levels of (A–C) globalmet.mod6 and (D–F) globalmet.mod8 among clinical trajectory groups. (A–C) Levels of globalmet.mod6, comprised of mostly branched amino acid and urea cycle metabolites, (A and B) at visit 1 and (C) over time. (D–F) Levels of globalmet.mod8, which is comprised of phospholipid metabolites, were associated with severity at (D and E) visit 1 (adj.p = 7.33e−5) and (F) longitudinally. (B and E) For each boxplot, the vertical line indicates the median, the box indicates the interquartile range, and the whiskers indicate 1.5 times the interquartile range.

Untargeted metabolomics using mass spectrometry (LC-MS) was performed on 1,275 plasma samples from 486 participants. After quality control and assurance procedures (Figures S6A–S6C), we identified 1,017 metabolite features based on their *m*/*z* ratio and retention time. PCA (Figure S6D) and PVCA identified event location (outpatient vs. inpatient, 11.5% variance for baseline, 10.5% variance for longitudinal analysis; Figure S6E) and body mass index (BMI; 14% variance for visit 1 analysis; Figure S6F) as accounting for a significant fraction of the variance. These factors were subsequently included as covariates in the longitudinal models. WGCNA identified 42 modules ranging from 5 to 296 metabolites (Figures S7A and S7B). Eighteen out of 42 modules measured at visit 1 were significantly associated with clinical outcome (Table S7). Seven of these modules demonstrated higher levels in participants with mild disease, while 11 modules were associated with severe disease (TG5). This included branched-chain aa and urea cycle metabolites (globalmet.mod6), phenylalanine and tyrosine metabolism (globalmet.mod35), and monoacylglycerol metabolism (globalmet.mod24). Interestingly, one of these modules, globalmet.mod6, consisted of branched aa and urea cycle metabolites (Figure 4A) and had a higher level in the more severe trajectory groups (Figure 4B; adj.p = 2.87e−13) raising the possibility of a role in disease severity. Consistent with this hypothesis, the module levels eventually decreased over time in the milder trajectory groups (TG1–4) but significantly increased across time in the most severe trajectory group (TG5) (shape adj.p = 2.95e−9, average adj.p = 4.3e−29) (Figure 4C). Longitudinal analysis also identified 26 additional modules with average or shape having a significant association with clinical trajectory group (Table S7). Among the most significant modules associated with trajectory groups, we identified globalmet.mod8, composed of many phospholipid metabolites including arachidonic acids (Figure 4D), as having higher concentration in participants with mild disease at hospital admission (Figure 4E; adj.p = 7.33e−5). This module also increased over time in all but the fatal group (TG5), where levels eventually decreased over time (Figure 4F; shape adj.p = 7.98e−4, average adj.p = 3.11e−8). We identified additional pathways such as histidine metabolism (globalmet.mod3) and glycerophospholipids (globalmet.mod21) that demonstrated the same decreasing pattern. Overall, this analysis identified significant dysregulation of the plasma metabolome associated with disease severity. Increases in plasma concentrations of branched-chain aa metabolites, including those within the histidine, lysine, urea, and tryptophan pathways, were associated with more severe disease trajectories (Figure S7C). In contrast, severe disease was also associated with lower and decreasing concentrations of phospholipid metabolites (Figure S7D).

### Cell frequencies in blood of severe hospitalized COVID-19 participants show high frequencies of neutrophils and monocytes, with decreased cytotoxic NK cells

**Figure 5:**
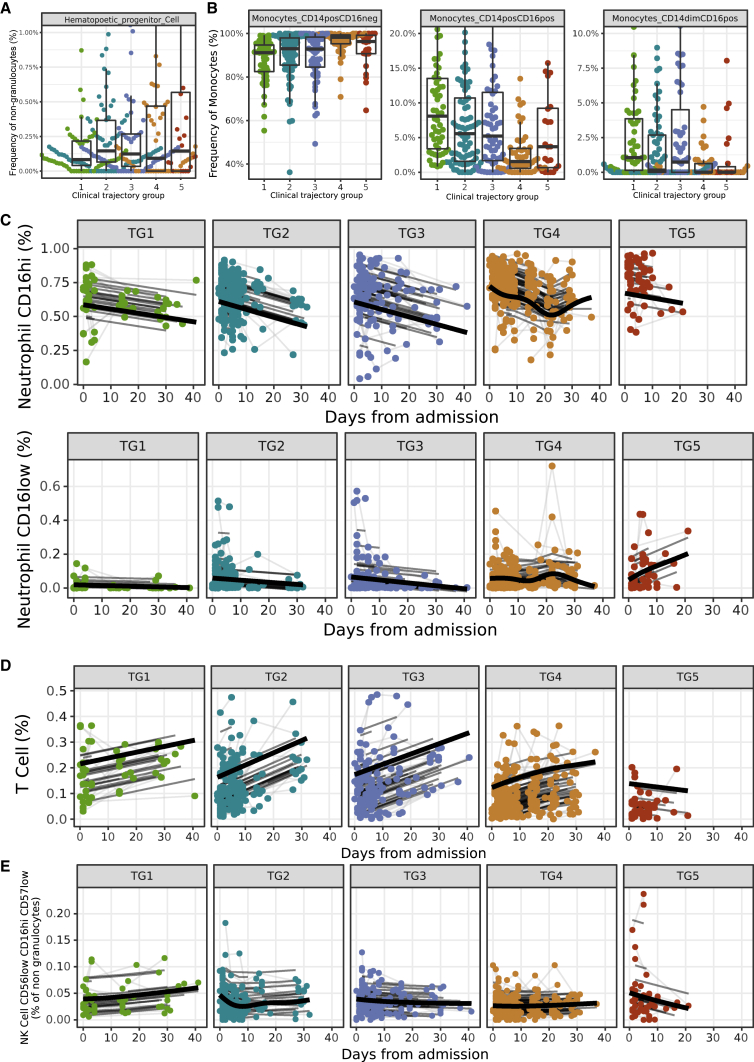
Association of cell subset frequencies with clinical trajectory groups (A) Visit 1 analysis identified the frequency of hematopoietic progenitor cells (HPCs) among non-granulocytes as different among clinical trajectory groups (adj.p = 6.34e−3), with higher average expression in the more severe groups. (B) The frequencies of CD14^+^CD16^−^, CD14^+^CD16^+^, and CD14^dim^CD16^+^ monocyte subsets among parental monocytes at visit 1. (A and B) For each boxplot, the vertical line indicates the median, the box indicates the interquartile range, and the whiskers indicate 1.5 times the interquartile range. (C–E) Longitudinal analysis of (C) neutrophil subset frequencies (CD16hi, average adj.p = 9.76e−4, shape adj.p = 6.74e−3; CD16low, average adj.p = 0.0109, shape adj.p = 0.0310), (D) T cell frequencies (average adj.p = 6.01e−7, shape adj.p = 0.0123), and (E) cytotoxic NK cell frequencies among non-granulocytes.

CyTOF profiling was performed on 811 blood samples collected from 371 participants. We used a panel of 43 antibodies designed to identify cell lineages and intracellular markers of functional status. Sixty-five cell subsets were identified in whole blood using a semi-automated gating strategy (Figure S8A). We did not detect any batch effect using PCA (Figure S8B) and PVCA (Figure S8C). The frequencies of 9 cell subsets measured at visit 1 were significantly associated with clinical outcome. Specifically, higher frequencies of lymphocytes, including T cells and NK cells, were associated with mild disease trajectories (TG1–3). In addition, higher frequencies of neutrophils, hematopoietic progenitor cells (adj.p = 6.34e−3; Figure 5A), and CD14^+^CD16^−^ classical monocytes (CD14^+^CD16^−^: adj.p = 3.83e−4, CD14^+^CD16^+^: adj.p = 3.73e−4; Figure 5B) were associated with more severe disease trajectories. Some of these cell subsets also showed significant changes over time that were associated with clinical trajectory groups. Indeed, participants in the most severe trajectory group (TG5) had a higher frequency of neutrophils at admission (primarily driven by CD16hi neutrophils). While this subset tended to decrease over time, the CD16low neutrophils increased over time in the severe trajectory group (TG5) (Figure 5C). This contrasts with participants that recovered, who had either constant or decreasing frequencies of neutrophils (both CD16hi and CD16low) over time (CD16hi, average adj.p = 1.08e−3, shape adj.p = 6.62e−3; CD16low, average adj.p = 0.0113, shape adj.p = 0.0317; Figure 5C). The frequency of total CD4 and CD8 T cells increased over time in all trajectory groups except for the most severe trajectory group (CD4, average adj.p = 4,18e−9, shape adj.p = 0.0251; CD8, average adj.p = 1.15e−4, shape adj.p = 0.0105; Figure 5D; Table S8), which saw instead an increase of myeloid cells over time (Figure S8D). The increase of CD4 and CD8 T cells in those aforementioned trajectory groups was driven by increases among many of the CD4 and CD8 cell subsets including CD4 and CD8 naive T cells, effector memory CD4 and CD8 T cells, and regulatory T cells (Tregs). Longitudinal analysis also revealed that the frequency of cytotoxic NK cells producing granzyme B (CD56low, CD16hi, CD57low) increased over time in participants in TG1–4, while in the most severe trajectory group (TG5), cytotoxic NK cell frequencies decreased over time (shape adj.p = 7.08e−7; Figure 5E; Table S8). Altogether, we identified immune cells distinguishing the five clinical trajectory groups including an increase in hematopoietic progenitor cells and classical monocytes that was persistent over time in participants with the most severe disease course. The heightened frequency of hematopoietic progenitor cells may reflect the emergency hematopoiesis that occurs in the most severe participants, while heightened pro-inflammatory monocytes may reflect the sustained and uncontrolled inflammation exacerbated by severe COVID-19. We also identified lymphopenia, neutrophilia, and a decrease in cytotoxic NK cells as associated with COVID-19 disease severity.

### Analysis of PBMC transcriptomics highlights modules related to inflammation and immune cell differentiation

**Figure 6:**
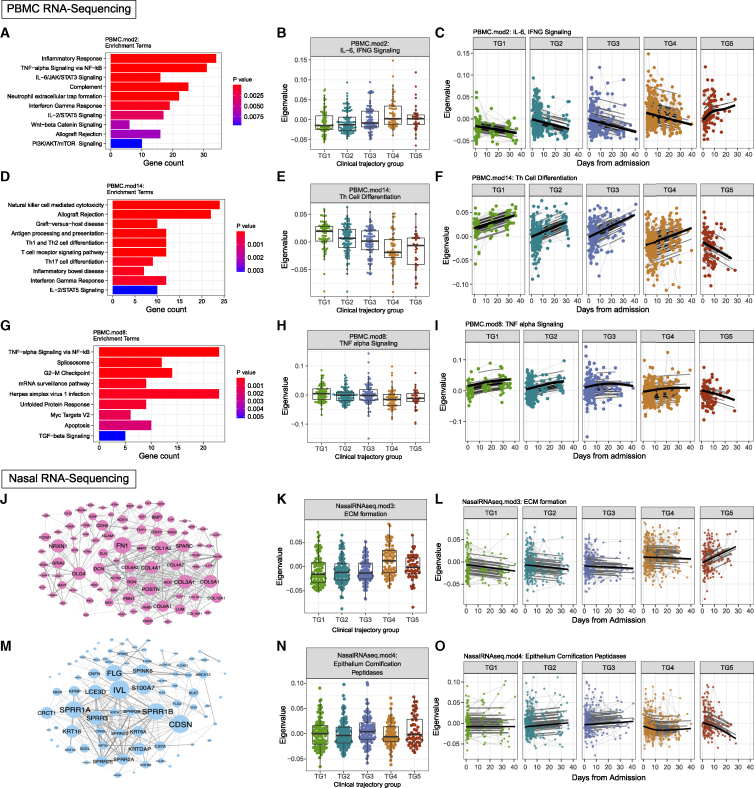
Association of PBMC transcriptomic and nasal transcriptomic modules with clinical trajectory groups (A–I) Analysis of PBMC transcriptomic data identified 21 modules with significant differences in expression levels between clinical trajectory groups at visit 1, including (A–C) PBMC.mod2, (D–F) PBMC.mod14, and (G–I) PBMC.mod8. (A, D, and G) These modules were interpreted using the top 10 enriched terms by MSigDB Hallmark,^39^ Reactome,^40^ and KEGG^41^ pathway databases ranked by p value after filtering for significant pathways with p <0.05. (B, E, and H) Module expression over trajectory groups at visit 1. (C, F, and I) Module expression by trajectory group over time. (J–O) Analysis of nasal transcriptomic data identified 7 modules with significant differences in expression levels among clinical trajectory groups, including (J–L) module 3 (NasalRNAseq.mod3) and (M–O) module 4 (NasalRNAseq.mod.4). Networks of protein-protein interactions among genes in (J) module 3 and (M) module 4 were retrieved from STRINGdb.^42^ Size of a node denotes degree, and edge thickness denotes strength of interaction as provided by STRINGdb.^42^ (B, E, H, K, and N) For each boxplot, the vertical line indicates the median, the box indicates the interquartile range, and the whiskers indicate 1.5 times the interquartile range.

We generated transcriptional profiles by RNA-seq for 1,033 PBMC samples from 429 participants. Batch effects were assessed using PCA (Figures S9A and S9B) and PVCA (Figure S9C). WGCNA identified 40 modules ranging from 86 to 1,676 genes. Twenty-one of these modules measured at visit 1 were significantly associated with clinical trajectory groups (Figure S9D). Among these 21 modules, PBMC.mod2 (containing 802 genes) was enriched for several pathways that have previously been associated with COVID-19, including TNF-α signaling via nuclear factor κB (NF-κB) inflammatory response,^36^ IFN-γ response,^37^ and IL-6/JAK/STAT3 signaling^38^ (Figure 6A). This module showed higher expression at visit 1 in participants from the more severe trajectory groups (TG4-5; adj.p = 7.99e−3; Figure 6B; Table S9) and showed a statistically significant change in the shape of expression over time between the trajectory groups (shape adj.p = 0.025, average adj.p = 1.33e−10; Figure 6C).

A module with high statistical significance, both at visit 1 (adj.p = 1.78e−8) and longitudinally (shape adj.p = 1.64e−7, average adj.p = 2.77e−20), was PBMC.mod14, containing 356 genes. Enriched pathways for PBMC.mod14 included Th17 cell differentiation, Th1 and Th2 cell differentiation, T cell receptor signaling, and IL-2/STAT5 signaling^36^ (Figure 6D). PBMC.mod14 showed decreasing expression at visit 1 with increasing disease severity (Figure 6E). Additionally, this module showed increasing expression over time in trajectory groups that ultimately recovered (TG1–4) but decreasing expression in participants in the fatal trajectory group (TG5), suggesting a role in disease recovery (Figure 6F).^37^ PBMC.mod29 also contained genes relating to T cell receptor signaling (Figure 6D).^38^ PBMC.mod8 consisted of 416 genes with functions relating to TNF-α signaling via NF-κB and transforming growth factor β (TGF-β) signaling (Figure 6G). Higher expression of PBMC.mod8 (Table S9) at visit 1 was associated with milder disease trajectories (adj.p = 2.76e−4; Figure 6H). Like PBMC.mod14, the expression of PBMC.mod8 generally increased over time in all but the most severe trajectory group (TG5) with decreasing expression over time (shape adj.p = 0.03, average adj.p = 1.39e−9; Figure 6I).

Interestingly, in multiple cases, the same pathway was found to be enriched in modules with opposing associations with trajectory group. For example, the TNF-α via NF-κB, IL-2/STAT5, and TCR signaling pathway genes that were part of PBMC.mod2 generally increased with trajectory group (at visit 1 and longitudinally). These same pathways have genes that were decreasing in PBMC.mod8 (TNF-α via NF-κB) and PBMC.mod14 (IL-2/STAT5 and TCR signaling). The genes driving each of these enrichments were distinct, as each gene is only associated with a single module (Figure S9E), and also reflected different biological mechanisms. Genes belonging to TNF-α via the NF-κB pathway in PBMC.mod2 were downstream of signaling from TNFR1 (TNF receptor 1), including the receptor itself, while only PBMC.mod8 genes were downstream of TNFR2 (Figure S10A). Overall, these results identify gene expression changes in multiple pathways associated with disease severity at both visit 1 and over time.

### Genetic associations with severe disease overlap previously reported Human Genome Initiative association

To identify genetic determinants of severe disease, we generated a high-quality dataset of 466 participants genotyped at 1,060,358 common variants across the genome, including the X and Y chromosomes. After controlling for population stratification (genomic inflation factor λ = 0.98) and other quality assessment measures (Figures S11A–S11E), we performed a GWAS of severe illness (TG4–5 vs. TG1–3). Given the sample size and modest effect sizes of common variants, no marker reached the genome-wide significant threshold of p < 5 × 10^−8^ (Figure S11F). We were, however, able to replicate one of ten associations with COVID-19 hospitalization previously reported by the Human Genome Initiative^43^ (11-34528766-C-T, p = 0.03; Table S10), which was linked to a role for ELF5, a transcription factor active in epithelial cells. This observation suggested that the genetic basis of severe disease in our cohort is similar to that reported by the HGI.^44^

### Dysregulated airway epithelial barrier functions relate to disease severity and mortality

We generated host transcriptional profiles from nasal swab samples to assess the upper airway mucosal responses, the initial site of SARS-CoV-2 infection and first line of barrier and immunologic defense to the virus. RNA-seq data was generated for 1,078 nasopharyngeal swab samples collected from 451 participants. After correcting for technical covariates (plate and median CV), PCA (Figure S12A) and PVCA did not reveal any batch effects (Figures S12B and S12C). WGCNA identified eight modules with size ranging from 92 to 1,761 genes (Figure S12D). Overall, the expressions of three out of eight modules were significantly associated with clinical outcome at visit 1, and six modules were associated with clinical outcome on the longitudinal pattern. NasalRNASeq.mod1 was enriched for genes related to multiple innate immune signaling pathways including neutrophil activation, IL-6, IL-1, TNF-α, Toll-like receptors, and type 1 and type 2 IFN signaling, among others, and was higher in participants with more severe disease trajectories (TG4–5) (Figure S12E). The NasalRNASeq.mod3 was enriched for genes involved in extracellular matrix formation including fibronectin 1 (*FN1*), periostin (*POSTN*), and 16 collagen genes and also enriched in genes associated with cell-cell adhesion and epithelial mesenchymal transition (Figure 6J). Expression of this module was increased in more severe trajectory groups (TG4 and TG5) at visit 1 (Figure 6K) and decreased over time in all groups but the most severe trajectory group (TG5), where the opposite pattern was observed (shape adj.p = 0.004, average adj.p = 3.08e−13; Figure 6L; Table S11), suggesting a role in disease severity. NasalRNASeq.mod4 was enriched for genes involved in epithelial cornification including filaggrin (*FLG*), *SPINK5*, and 11 keratin genes and was also enriched in serine-type peptidases including tissue kallikreins (Figure 6M). In contrast, the expression of NasalRNASeq.mod4 was lower in participants in the more severe trajectory groups (TG4 and TG5) (Figure 6N) and decreased over time, specifically in TG5 (shape adj.p = 0.018, average adj.p = 0.07; Figure 6O; Table S11). Overall, this analysis identified significant dysregulation of airway epithelial barrier responses that were associated with disease severity and mortality. In particular, a multi-faceted inflammatory response occurs directly in the airway in severe COVID-19 as well as increased expression of extracellular matrix, adhesion, and collagen genes that may represent the initial cellular damage driving severe inflammation.

### Analysis of upper airway metagenomics reveals abundance in anaerobes in more severe trajectory group

In our previous publication describing clinical features of the entire IMPACC cohort, we noted differences in bacterial infections based on trajectory group, with bacteremia clinically reported in a higher proportion of participants in TG4 (45/212, 21%) and TG5 (28/108, 26%) than TG1–3 (40/844, 4.7%). Here, we performed meta-transcriptomic analysis on the same host nasal RNA-seq data generated from 1,077 nasopharyngeal samples collected from 451 participants. PCA (Figures S13G and S13H), non-metric Bray-Curtis dissimilarity analysis, and PVCA did not reveal any batch effects (Figure S13I). There was no significant association of bacterial abundance (Figure S13A) or α diversity (Figure S13B) with clinical trajectory either at visit 1 or longitudinally. The relative abundance of bacterial genera at visit 1 also showed no significant associations with clinical trajectory. However, the longitudinal patterns of 22 bacterial genera were significantly associated with clinical outcome (Table S12). The relative abundance of anaerobic bacteria including *Bacteroides spp*. (shape adj.p = 0.038, average adj.p = 5.4e−4; Figure S13C), *Fusobacterium spp*. (shape adj.p = 0.25, average adj.p = 0.001; Figure S13D), and *Prevotella spp*. (shape adj.p = 0.0501, average adj.p = 5.4e−4; Figure S13E) was higher overall in more severe trajectory groups, and the expression of these bacterial genera increased over time in the most severe trajectory group (TG5). In contrast, the relative abundance of 10 bacterial genera, including the well-known commensal *Cutibacterium spp*., was lower overall and further decreased over time in the most severe trajectory group (TG5) (shape adj.p = 0.16, average adj.p = 1.7e−5; Figure S13F). Overall, this analysis identified temporal changes in the relative abundance of multiple bacterial taxa that were associated with disease severity and mortality. These changes in upper airway microbial communities may influence inflammatory signaling or viral replication.

### Overlap across data types reveals consistent pathways associated with disease severity

**Figure 7:**
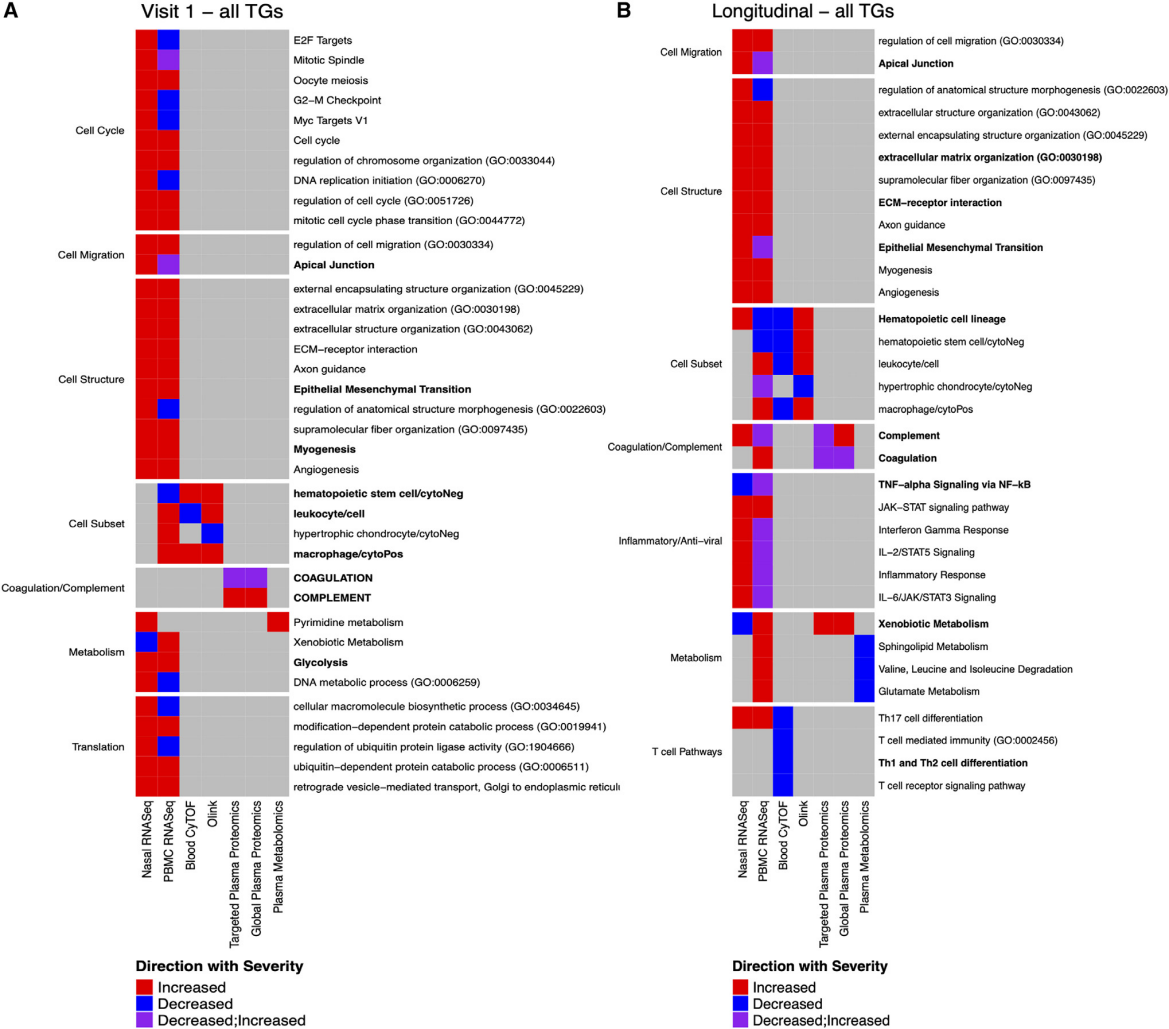
Markers of disease severity overlapping across assays Overlapping pathways associated with more moderate or more severe trajectory groups (A) at the time of hospitalization (visit 1; left) or (B) during the longitudinal follow up during the acute phase of the disease (right). For each overlapping pathway (row), the assays contributing to its identification as a marker of COVID-19 disease severity (column) are indicated. The color of each cell reflects whether the pathway is associated with moderate (blue) or severe (red) disease or both (purple). Pathways were manually separated into groups of biologically related processes based on their names.

The analysis of each assay identified many modules that were significantly associated with clinical TG, both at visit 1 and longitudinally. We assessed the overlap of pathways that were enriched in these modules to identify common biological processes across data types (e.g., mRNA and protein) and tissues (i.e., blood and upper airways). Among the modules that were significantly associated with TGs at visit 1, 37 pathway annotations were enriched in multiple data types (Figure 7A). The most overlapping annotation was related to monocytes/macrophages and was associated with PBMC transcriptomics and blood CyTOF as well as Olink. This included genes coding for myeloid cell-specific markers CD93 and Toll-like receptor 4 (TLR4) and the soluble proteins CCL4 (MIP-1β) and TNFSF14 (LIGHT), known to activate macrophages and abrogate T cell responses, as well as elevated frequencies of monocytes among the most severe COVID-19 cases.^45^^,^^46^^,^^47^ In general, overlapping annotations were shared between PBMC and upper airway transcriptional responses. Pathways related to cell cycle and cell migration were perturbed in both tissue compartments. In the upper airway, higher expression of these modules was generally associated with more severe disease trajectory groups, suggesting more active, localized responses in severe disease. Common pathways enriched among modules identified in the longitudinal (shape) analysis highlighted additional disease-associated perturbations (Figure 7B). In particular, inflammatory responses and T cell-associated pathways were observed in both PBMC and upper airway transcriptomics assays. The positive and negative associations of T cell-associated pathways with severe disease in the upper airways and blood, respectively, may reflect the migration of these subsets out of the blood. Two pathways (xenobiotic metabolism and complement) were observed in four separate assays: PBMC and upper airway transcriptomics along with targeted and global proteomics. The association of xenobiotic metabolism was driven by multiple genes (CYP1B1, ALDH2, and CES1 were part of PBMC.mod18) and proteins (APOE part of Targeted.Prot.mod4 and CRP part of the Global.prot.mod3) in the pathway. The association of xenobiotic metabolism with increased severity is likely a reflection of the large metabolomic reprogramming experienced by severe COVID-19 participants. The association of complement was driven by multiple genes (CR1, C5AR2, C5AR1 part of the PBMC.mod2 and genes CD40LG part of the NasalRNAseq.mod6) and proteins (CP, CFB, C9 part of the Targeted.Prot.mod3 and Global.prot.mod3). Complement activation products orchestrate a pro-inflammatory environment that contributes to the maintenance of a severe inflammatory response to SARS-CoV-2 and is likely to cause several of the symptoms observed after infection.

## Discussion

For a comprehensive profile of acute COVID-19, we have undertaken unbiased large-scale transcriptomic, proteomic, metabolomic, cytometric, serologic, genomic, microbiome, and viral state analyses of 540 hospitalized COVID-19 participants, recruited from 20 hospitals associated with 15 biomedical centers, who were longitudinally followed up to 28 days post-admission. Major advantages of IMPACC include the prospective enrollment of diverse adult populations from across the US and sample sparing assays of blood and tissue/fluids using comprehensive molecular omics methods. Additionally, the collection of extensive clinical data allows for identification of five distinct clinical trajectories that discriminated ranges of respiratory disease severity.^11^ This clinical phenotyping has advantages over conventional cross-sectional assessments by fully leveraging longitudinal data indicative of respiratory illness severity to characterize a participant’s outcomes during hospitalization, from mild respiratory disease (TG1) to severe respiratory disease ending in death (TG5). Using this systems analysis approach, we both confirm the findings of immune dysregulation from smaller, cross-sectional cohorts as well as identify cellular and soluble factors, at hospital admission and longitudinally, that are associated with disease severity and death from SARS-CoV-2 infection. Higher viral load and elevated inflammatory pathways in the airway are linked to more severe COVID-19 in this cohort.

Further characterization of molecular factors that are associated with disease trajectories enable identification of distinct cellular and molecular mechanisms that contribute to a fatal outcome. A primary finding is the association of delayed viral clearance with death, despite detectable antibody responses, which suggests ongoing viral replication and potential differences in antibody quality or functionality in those with fatal outcomes. While antibody quality and functionality were not directly assessed, individuals who died exhibited increased seroreactivity to the NTD of S and decreased reactivity to the LINK domain of N. In addition, consistent with prior studies,^2^ participants with more severe COVID-19 had neutralizing autoantibodies (auto-Abs) against type I IFNs (TG4 = 9.6%, TG5 = 7.8%) that may contribute to the severity of disease in these individuals.

Lack of direct correspondence between viral loads and anti-viral antibody response suggests that dysregulation in other elements of the immune response plays a role in fatal cases.^2^ Immuno-profiling of innate and adaptive leukocyte subsets in blood using CyTOF and RNA-seq reveals that the most severe trajectory group (TG5) had a lower frequency of granzyme B-producing cytotoxic NK cells and lower expression of cytotoxic gene pathways. NK cells kill virally infected cells,^48^ and reduced levels of these cells may contribute to the viral persistence in TG5. Notably, analysis of cytokine/chemokine expression (Olink) identified a significant increase of activators of cytotoxic NK cells, including IL-12B and the immunoregulatory signaling molecule CD244, in less severe infection.^49^ We also found decreased phospholipid components, including phosphatidylcholines, associated with more severe disease trajectories. Phosphatidylcholines contribute to the formation of the immunological synapse, macrophage activation, NK cell function and T and B cell activity^50^^,^^51^ underlying severe/fatal disease,^49^^,^^50^ suggesting a role for these metabolites in regulating anti-viral immunity and promoting protection from severe disease. In summary, our results identified a deficiency of NK cell subsets and activity that could lead to impaired viral clearance as a mechanism underlying severe/fatal COVID-19.

More severe disease trajectories were associated with the activity of multiple pro-inflammatory pathways at baseline, and this activity persisted in people who did not survive the infection (TG5). Active pathways include TNF-α signaling via NF-κB, IL-6 signaling, and the IL-6/Jak/STAT3 pathway as noted previously in smaller cross-sectional studies.^52^ Genes contained within the TNF-α signaling pathway that displayed both increased gene expression at the initial visit and increasing expression over time in TG5 were found exclusively in genes known to be downstream of the TNFR1, but not TNFR2, including TNFR1 itself. Among genes downstream of TNFR1, c-FLIP, which functions to inhibit apoptosis and stimulate inflammatory components of the TNF-α signaling pathway,^53^ was also increased in expression in TG5. Inflammatory cell death induced by TNF and IFN-γ signaling has been linked to COVID-19 mortality.^54^ Supporting this pro-inflammatory role of the TNF signaling pathway is the combined expression of leukocyte recruitment factors CXCL1/2/3. Additionally, contained in PBMC.mod8 (showing decreasing expression in TG5) is c-Jun, a transcription factor that activates pro-apoptotic genes. These key components of the TNF pathway, though enriched in modules showing opposite expression trajectories, show that anti-apoptosis and pro-inflammatory mechanisms are activated in more severe trajectory groups. These inflammatory cytokines can recruit pro-inflammatory innate immune cells including monocytes and neutrophils, which will amplify inflammatory pathways leading to a “cytokine storm.”

Our results reveal a hyperinflammatory state across the airway and systemically as a correlate of severe infection and death. CyTOF analysis also shows a greater frequency of neutrophils in the more severe trajectory groups 4 and 5, a marker of severe COVID-19 outcome as noted previously^55^^,^^56^ and possibly reflecting secondary bacterial infection.^57^ Neutrophil influx into the lung may damage lung epithelial cells and contribute to lung pathology, which may be amplified by release of NETs and neutrophil granule contents. In addition, cytokine/chemokine assays (Olink) identified multiple modules associated with disease severity including cytokines produced by neutrophils, pro-inflammatory modules, and activators of macrophages. Inflammatory biomarkers, including IL-6, were higher at baseline in both TG4 and TG5 compared with milder disease, similar to previous findings.^58^ Longitudinal Olink measurements suggest a clear association between resolution of inflammation in 28-day survivors (TG1-4) vs. non-survivors (TG5), confirming the value of evaluating both clinical outcomes and measures of inflammation over time rather than in a cross-sectional fashion. Elevated products of neutrophils identified by nasal and blood RNA-seq are all associated with TG4 and TG5 and remained elevated over time. Examining metabolite profiles, we identified plasma branched-chain aa (BCAA) and urea components as significantly elevated at baseline and further increased over time with severe trajectories. Increased BCAA components enhance reactive oxygen species (ROS) production, endothelial cell pro-inflammatory activities,^59^^,^^60^ and insulin resistance.^61^ Histidine and lysine residues, often found in viral envelope proteins, play roles in the activation of serine proteases assisting viral entry to host cells.^62^^,^^63^ RNA-seq analysis of upper airway samples identified that severe/fatal disease is associated with higher initial and increased subsequent expression of genes related to extracellular matrix formation and cell adhesion, including fibronectin.^64^^,^^65^ These findings suggest a potential etiology for our plasma proteome results that demonstrated thrombosis.^64^^,^^65^ Our data suggest that in severe COVID-19, adverse remodeling of the airway epithelium, the first line of barrier and immunologic defense against respiratory viruses and the initial site of infection for SARS-CoV-2, may initiate a prothrombotic state systemically.^53^^,^^54^^,^^55^

Increased plasma concentrations of various myosin chains of cardiac and/or musculoskeletal origin were detected in the most severely ill COVID-19 participants. This provides evidence for damage to skeletal and cardiac muscle tissues in severe COVID-19 and might reflect damage to the blood vessels and myocardium, as well as muscle breakdown from a catabolic stress response. Muscle damage is associated with a poor prognosis in COVID-19.^66^ In our study, all COVID-19 participants that eventually recovered (TG1–4) show a slight increase or steady state of plasma fibrinolysis stimulators and coagulation inhibitors. Coagulation is a carefully balanced counterplay of thrombosis (blood clot formation) and fibrinolysis (breakdown of blood clots). The observed relationship between a massively dysregulated coagulation cascade and disease severity is consistent with the widely reported blood clotting complications in COVID-19 participants. For example, elevated plasma levels of D-dimer, a fibrin-degradation product, are a marker of increased risk of severe disease and mortality.^67^

Changes in the respiratory microbiome may moderate inflammatory gene expression, immune signaling, and viral replication. We found an enrichment of anaerobes in the genera *Prevotella* and *Bacteroides* in the upper airways of participants with more severe trajectories. Conversely, we found that commensal *C*. *spp*. were enriched in participants with milder trajectories, whereas loss of these species over time was associated with fatal disease. These results suggest a possible role for some taxa in disease pathogenesis, or alternatively, they may reflect disruption of the upper airway microbiome resulting from the host immune response to SARS-CoV-2 infection. The observations may also, in part, reflect greater exposure of those with more severe COVID-19 to antibiotics.^68^ Future work can extend these observations to both build improved prognostic models and understand the specific contributions of these taxa to respiratory tract inflammation and viral replication.

Our study also identified elements that may be protective from severe disease. Notably, in the upper airway epithelium, mild disease was associated with higher expression of genes related to epithelial cornification typically seen in squamous epithelium, whereas this pathway declined significantly in fatal disease. Given that SARS-CoV-2 does not replicate significantly in squamous epithelium and that multi-ciliated cells are the primary site of SARS-CoV-2 infection,^69^^,^^70^ this finding suggests a protective response mediated via epithelial reprogramming toward squamous cells that can generate local anti-viral responses.^68^ This finding is also consistent with the higher viral load and prolonged viral shedding associated with fatal disease. Additionally, we identified genes from PBMC pathways related to T cell receptor signaling, in which Th1, Th2, and Th17 cell differentiation was increased in disease recovery groups (TG1–4). These findings are consistent with observations of lymphopenia in COVID-19 cases^71^ and later findings that altered T cell activity and decreased abundance were also associated with severe disease.^3^

Overall, our study featured multiple strengths, including (1) a large, geographical diverse cohort compared with most COVID-19 studies employing omics approaches, (2) longitudinal design with extensive clinical data capture, (3) immunophenotyping employing 14 assay types, and (4) rigorous data management, quality control and assurance, and a standardized analysis pipeline. This comprehensive approach enabled deep immunophenotyping of the acute phase of COVID-19 from 540 hospitalized participants enrolled in the IMPACC cohort and identified several significant associations with clinical course. Specifically, we identified decreases in activators of NK cells and phospholipid metabolites, increased blood neutrophils, increased circulating myosins that may indicate muscle damage, changes in the cells that line the airways (epithelial reprogramming), and an increased abundance of anaerobes in the airway of participants the succumbed to SARS-CoV-2 infection. Broadly, these results point to heightened levels of viremia driving an inflammatory response locally and systemically, leading to impaired anti-viral innate and adaptive immunity as well dysregulation in metabolic pathways in participants with severe disease trajectories. While many of the perturbed pathways were observed in multiple assays, most were unique to a single assay, highlighting the utility of a multi-omics approach.

### Limitations of the study

While featuring multiple strengths, potential limitations of our study include (1) the identification of associations but not cause-effect relationships, (2) the lack of immunophenotyping of the pre-infection biologic state, which could influence disease progression, or healthy control participants for comparison, (3) the exclusion of pregnant women and children,^10^ and (4) the timing of cohort enrollment before vaccination or the widespread circulation of important variants, including SARS-CoV-2 B.1.617.2 (Delta) and B.1.1.529 (Omicron). While this study employed a common analytic strategy across modalities and tissues, allowing the identification of likely shared biological drivers, modules were defined separately for each assay and were analyzed independently. An alternate analysis that starts by defining multi-omics modules as the unit for analysis would allow for the direct identification of correlations between features and associated pathways. In some cases where common pathways were identified by multiple assays (Figure 7), their association with severe disease was discordant. In these cases, it is possible that distinct components of the pathway drive significance in each assay (e.g., up-regulation of inhibitory cytokines and down-regulation of the associated pathway) or that the changes reflect cell migration (e.g., migration of activated cells from blood to the upper airways). Some of the associations with COVID-19 disease severity may also be confounded by clinical treatment (e.g., medications administered to manage COVID-19). However, the analysis of the entire IMPACC cohort did not detect any impact of either remdesivir or systemic corticosteroid use on nasal viral load or SARS-CoV-2 serology titers.^11^ Future analysis of the full IMPACC cohort with deep immunophenotyping data may allow for an assessment of the effect of medications.

## Consortia

The members of the IMPACC Network are James Abraham, Michael Adkisson, Marisa Albert, Luz Altamirano Torres, Bonny Alvarenga, Matthew L. Anderson, Evan J. Anderson, Azlann Arnett, Hiromitsu Asashima, Mark A. Atkinson, Lindsey R. Baden, Brenda Barton, Katherine Beach, Elizabeth Beagle, Patrice M. Becker, Matthew R. Bell, Mariana Bernui, Christian Bime, Arun Boddapati Kumar, J. Leland Booth, Brittney Borresen, Steven E. Bosinger, Scott C. Brakenridge, Laurel Bristow, Anderson Brito Fernandes, Robert Bryant, Charles B. Cairns, Carolyn S. Calfee, Juan Carreño Manuel, Sidney Carrillo, Suzanna Chak, Ana C. Chang, Iris Chang, Jing Chen, Ernie Chen, Jennifer Connors, Michelle Conway, David B. Corry, Chris Cotsapas, David Cowan, Brett Croen, Charles S. Dela Cruz, Gina Cusimano, Ravi Dandekar, Joann Diray-Arce, Lily Eaker, Walter Eckalbar, Carolyn Edwards, Lauren I.R. Ehrlich, David Elashoff, Heidi Erickson, David J. Erle, Denise Esserman, Shelli Farhadian, Keith Farrugia, Benoit Fatou, Andrea Fernandes, Ana Fernandez-Sesma, Slim Fourati, Gabriela K. Fragiadakis, Sara Furukawa, Janelle N. Geltman, Rajani Ghale, Ana Gonzalez-Reiche Silvia, Maria González Carolina Bermúdez, I. Michael Goonewardene, Nathan D. Grubaugh, Leying Guan, Estella Guerrero Sanchez, Faheem W. Guirgis, Jeremy Gygi, Elias K. Haddad, David A. Hafler, Sydney Hamilton, Paul Harris, Arash Hayati Nemati, Carolyn M. Hendrickson, Nelson I. Agudelo Higuita, Annmarie Hoch, Thomas Hodder, Steven M. Holland, Catherine L. Hough, Christopher Huerta, Kerin C. Hurley, Scott R. Hutton, Akiko Iwasaki, Alejandra Jauregui, Naresh Jayavelu Doni, Meenakshi Jha, Brandi Johnson, David Joyner, Kirsten N. Kangelaris, Geoffrey Kelly, Zain Khalil, Zenab Khan, Farrah Kheradmand, Alvin T. Kho, James N. Kim, Seunghee Kim-Schulze, Hiroki Kimura, Steven H. Kleinstein, Albert I. Ko, Bernard Kohr, Anna Konstorum, Monica Kraft, Florian Krammer, Matthew Krummel, Michele A. Kutzler, Charles R. Langelier, Jessica Lasky-Su, Serena Lee, Brian H. Lee, Deanna Lee, Michael Leipold, Claudia Lentucci, Carolyn Leroux, Ofer Levy, Edward Lin, Shanshan Liu, Christina Love, Zhengchun Lu, Holden Maecker, Cole Maguire, Lenka Maliskova, Brittany Roth Manning, Monali Manohar, Mark Martens, Grace A. McComsey, Kerry McEnaney, Renee McLin, Esther Melamed, Nataliya Melnyk, Kevin Mendez, William B. Messer, Jordan P. Metcalf, Greg Michelotti, Eran Mick, Carly E. Milliren, Subhasis Mohanty, Ruth R. Montgomery Jarrod Mosier, Lubbertus C.F. Mulder, Maimouna Murphy, Kari R.C. Nadeau, Ebony Nelson, Allison Nelson, Viet Nguyen, Jordan Oberhaus, James A. Overton, Al Ozonoff, Bernadine Panganiban, Ravi Patel, Shrikant Pawar, Kathryn L. Pellegrini, Bjoern Peters, Harry C. Pickering, Debra L. Powell, Scott Presnell, Bali Pulendran, Jingjing Qi, Adeeb H. Rahman, Jayant Rajan, Ahmad Rashid Sadeed, Ariel Raskin, Elaine F. Reed, Susan Ribeiro Pereira, Adreanne M. Rivera, Jacob E. Rogers, Angela Rogers, Brandon Rogowski, Rebecca Rooks, Lindsey B. Rosen, Yael Rosenberg-Hasson, Jessica Rothman, Nadine Rouphael, Justin F. Rousseau, Ramin Salehi-Rad, Mehmet Saluvan, Hady Samaha, Joanna Schaenman, Andrew W. Schroeder, Ron Schunk, Rafick Sekaly, Nicholas C. Semenza, Subha Sen, Jonathan Sevransky, Vicki Seyfert-Margolis, Tanzia Shaheen, Albert C. Shaw, Scott Sieg, Sarah A.R. Siegel, Natalia Sigal, Nadia Siles, Brent Simmons, Viviana Simon, Gagandeep Singh, Lauren Sinko, Cecilia M. Smith, Kinga K Smolen, Li-Zhen Song, Komal Srivastava, Hanno Steen, Peter Sullivan, Caitlin Syphurs, Johnstone Tcheou, George P. Tegos, Greg K. Tharp, Alexandra Tong, Alexandra Tsitsiklis, Ricardo F. Ungaro, Tatyana Vaysman, Arthur Viode, Randi Vita, Xiaomei Wang, Alyssa Ward, Dawn C. Ward, Kerstin Westendorf, Andrew Willmore, Michael R. Wilson, Kyra Woloszczuk, Kari Wong, Prescott G. Woodruff, Leqi Xu, Harm van Bakel, Simon van Haren, Patrick van Zalm, Adriana van de Guchte, and Yujiao Zhao.

## STAR★Methods

### Key resources table

**Table:** 

REAGENT or RESOURCE	SOURCE	IDENTIFIER
**Antibodies**		

Maxpar® Direct™ Immune Profiling Assay (MDIPA) Kit	Fluidigm	Cat#201325
CD8a-146ND	Fluidigm	Cat#3146001B; RRID:AB_2687641
Granzyme B Antibody, anti-human/mouse/rat, REAfinity	Miltenyi	Cat#130-116-486
Goat Anti-Human IgA-UNLB	Southern Biotech	Cat#2050-01
Purified anti-human IgM Antibody	Biolegend	Cat#314502
Mouse Anti-Human IgG1 Fc-UNLB	Southern Biotech	Cat#9054-01
Purified anti-mouse/human CD11b Antibody	Biolegend	Cat#101202
Purified anti-human/mouse/rat CD278 (ICOS) Antibody	Biolegend	Cat#313502
Purified anti-human CD39 Antibody	Biolegend	Cat#328202
Purified anti-human CD169 (Sialoadhesin, Siglec-1) Antibody	Biolegend	Cat#346002
Purified anti-human CD64 (Maxpar® Ready) Antibody	Biolegend	Cat#305029
Purified anti-human CD71 Antibody	Biolegend	Cat#334102
Anti-Human CD279/PD-1 (EH12.2H7)-175Lu	Fluidigm	Cat#3175008B
Anti-Human CD61 (VI-PL2)-209Bi	Fluidigm	Cat#3209001B
Anti-Human CD3 (UCHT1)-141Pr antibody	Fluidigm	Cat#3141019B
Anti-Human HLA-DR (L243)-143ND antibody	Fluidigm	Cat#3143013B
Anti-Human CD69 (FN50)-144ND antibody	Fluidigm	Cat#3144018B
Anti-Human CD4 (RPA-T4)-145ND antibody	Fluidigm	Cat#3145001B
Anti-Human CD8a (RPA-T8)-146ND antibody	Fluidigm	Cat#3146001B
Anti-Human CD20 (2H7)-147Sm antibody	Fluidigm	Cat#3147001B
Anti-Human CD127 (A019D5)-149Sm antibody	Fluidigm	Cat#3149011B
Anti-Human MIP-1β (D21-1351)-150ND antibody	Fluidigm	Cat#3150004B
Anti-Human CD123 (6H6)-151Eu antibody	Fluidigm	Cat#3151001B
Anti-Human TNFα (Mab11)-152Sm antibody	Fluidigm	Cat#3152002B
Anti-Human CD62L (DREG-56)-153Eu antibody	Fluidigm	Cat#3153004B
Anti-Human CD45 (HI30)-154Sm antibody	Fluidigm	Cat#3154001B
Anti-Human IL-6 (MQ2-13A5)-156Gd antibody	Fluidigm	Cat#3156011B
Anti-Human IFN-γ (B27)-158Gd antibody	Fluidigm	Cat#3158017B
Anti-Human CD11c (Bu15)-159Tb antibody	Fluidigm	Cat#3159001B
Anti-Human CD14 (M5E2)-160Gd antibody	Fluidigm	Cat#3160001B
Anti-Human CD80/B7.1 (2D10.4)-161Dy antibody	Fluidigm	Cat#3161023B
Anti-Human CD66b (80H3)-162Dy antibody	Fluidigm	Cat#3162023B
Anti-Human CD56 (NCAM16.2)-163Dy antibody	Fluidigm	Cat#3163007B
Anti-Human CD15 (W6D3)-164Dy antibody	Fluidigm	Cat#3164001B
Anti-Human CD61 (VI-PL2)-165Ho antibody	Fluidigm	Cat#3165010B
Anti-Human CD11b (ICRF44)-167Er antibody	Fluidigm	Cat#3167011B
Anti-Human CD206 (15–2)-168Er antibody	Fluidigm	Cat#3168008B
Anti-Human CD54 (HA58)-170Er antibody	Fluidigm	Cat#3170014B
Anti-Human CD68 (Y1/82A)-171Yb antibody	Fluidigm	Cat#3171011B
Anti-Human CD16 (3G8)-209Biantibody	Fluidigm	Cat#3209002B
Anti- CoV Nucleocapsid protein (6H3) antibody	Abcam	Cat#ab273434
Anti-Human Eotaxin (43915) antibody	R&D	Cat#MAB3201
Anti-Human ACE-2 (535919) antibody	NOVUS	Cat#MAB9332-100
Anti-Human Cytokeratin (C-11) antibody	Biolegend	Cat#628602
Anti- CoV Spike protein (1A9) antibody	GeneTex	Cat#GTX632604
Anti-Human EPX (MM82.2.1) antibody	MAYO CLINIC	https://www.mayoclinic.org
Anti-Human IL-8 (E8N1) antibody	Biolegend	Cat#511402
Anti-Human IL-1β (H1b-27) antibody	Biolegend	Cat#511602
Anti-Human IFN-β (IFNb/A1) antibody	Biolegend	Cat#514002
Anti-Human Siglec-8 (837535) antibody	R&D	Cat#MAB7975
Anti-human IgG (Fc specific)-Peroxidase antibody produced in goat	Sigma-Aldrich	Cat#A0170; RRID: AB_257868
Goat anti-human IgM-HRP	SouthernBiotech	Cat#2020–05; RRID: AB_2795603
Anti-human IgA (α-chain specific)-Peroxidase antibody produced in goat	Sigma-Aldrich	Cat#A0295; RRID: AB_257876
Anti-Glial Fibrillary Associated Protein	Agilent	Cat#Z033429-2
Anti-human IgG (PE)	ThermoScientific	Cat#12-4998-8
Anti-human pSTAT1 (AF647)	BD	Cat#612597
Anti-human CD14 (FITC)	BD	Cat#555397

**Bacterial and virus strains**		

BLT5403, T7 Select Kit	Novagen	Cat#70550-3
T7 Bacteriophage, T7 Select Kit	Novagen	Cat#70550-3

**Biological samples**		

Plasma samples from IMPACC cohort	Multiple clinical sites	N/A
Whole blood from hospitalized COVID19 patients-collected in EDTA tubes	Multiple clinical sites	N/A
Veri-Cells™ Heavy Metal (Ta) PBMC	Biolegend	Cat#427203
Serum samples from IMPACC cohort	Multiple clinical sites	N/A
Stimulated Plasma from Healthy Controls	Stanford University	N/A
Plasma from Healthy Controls	Stanford University	N/A
Serum from Healthy Controls	Stanford University	N/A

**Chemicals, peptides, and recombinant proteins**		

DNA/RNA Shield Collection Tube w/Swab - DX	Zymo Research	Cat#R1107-E
Quick-DNA/RNA MagBead	Zymo Research	Cat#R2131
Stranded Total RNA Prep, Ligation with Ribo-Zero Plus	Illumina	Cat#20040529
HS NGS Fragment Kit	Agilent	Cat#DNF-474-0500
K-562 Total RNA	Thermo Fisher	Cat#AM7832
qScript XLT 1-Step RT-qPCR ToughMix	Quantabio	Cat#95133-02K
2-propanolol (LC-MS)	MilliporeSigma	Cat#1027814000
Acetonitrile (LC-MS)	MilliporeSigma	Cat# 1000294000
Water, Baker Analyzed LC/MS Reagent Grade	J.T. Baker	Cat#9831-02
Ammonium Formate (LC-MS)	J.T. Baker	Cat#M530-08
Perfluoropentanoic acid	Sigma	Cat#396575
Ammonium Bicarbonate	Fisher	Cat#A643
Ammonium Hydroxide	Sigma	Cat#338818
Cell-ID™ 20-Plex Pd Barcoding Kit	Fluidigm	Cat#201060
Saponin	Sigma	Cat#47036
Human TruStain FcX™ (Fc Receptor Blocking Solution)	Biolegend	Cat#422302; RRID:AB_2818986
Heparin sodium salt	Sigma	Cat#H3393
SmartTube PROT1 stabilizer PROT1-250ML	SmartTube	Fisher Cat# 501351692
SmartTube ThawLyse - THAWLYSE1	SmartTube	Fisher Cat# 501351696
Paraformaldehyde (PFA), 16% w/v aqueous, methanol-free	Alfa Aesar	Fisher Cat# AA433689L
Fetal bovine serum, characterized, heat-inactivated	HyClone	Fisher Cat#SH30396.03
Dimethyl sulfoxide	Fisher	Cat#BP231-100
Maxpar MCP9 Antibody Labeling Kit, 111Cd	Fluidigm	Cat#201111A
Maxpar MCP9 Antibody Labeling Kit, 112Cd	Fluidigm	Cat#201112A
Maxpar MCP9 Antibody Labeling Kit, 114Cd	Fluidigm	Cat#201114A
Maxpar MCP9 Antibody Labeling Kit, 116Cd	Fluidigm	Cat#201116A
Maxpar® X8 Antibody Labeling Kit, 142ND	Fluidigm	Cat#201142B
Maxpar® X8 Antibody Labeling Kit, 159Tb	Fluidigm	Cat#201159B
Maxpar® X8 Antibody Labeling Kit, 162Dy	Fluidigm	Cat#201162B
Maxpar® X8 Antibody Labeling Kit, 165Ho	Fluidigm	Cat#201165B
Maxpar® X8 Antibody Labeling Kit, 169Tm	Fluidigm	Cat#201169B
Maxpar® X8 Antibody Labeling Kit, 142Nd—4 Rxn	Fluidigm	Cat#201142A
Maxpar® X8 Antibody Labeling Kit, 148Nd—4 Rxn	Fluidigm	Cat#201148A
Maxpar® X8 Antibody Labeling Kit, 155Gd—4 Rxn	Fluidigm	Cat#201155A
Maxpar® X8 Antibody Labeling Kit, 166Er—4 Rxn	Fluidigm	Cat#201166A
Maxpar® X8 Antibody Labeling Kit, 169Tm—4 Rxn	Fluidigm	Cat#201169A
Maxpar® X8 Antibody Labeling Kit, 172Er—4 Rxn	Fluidigm	Cat#201172A
Maxpar® X8 Antibody Labeling Kit, 173Yb—4 Rxn	Fluidigm	Cat#201173A
Maxpar® X8 Antibody Labeling Kit, 174Yb—4 Rxn	Fluidigm	Cat#201174A
Maxpar® X8 Antibody Labeling Kit, 175Lu—4 Rxn	Fluidigm	Cat#201175A
Maxpar® X8 Antibody Labeling Kit, 176Yb—4 Rxn	Fluidigm	Cat#201176A
Cell-ID™ Cisplatin	Fluidigm	Cat#201064
Cell-ID™ Intercalator	Fluidigm	Cat#201192A
Cell-ID™ 20-Plex Pd Barcoding Kit	Fluidigm	Cat#201060
Maxpar® Water—500 mL	Fluidigm	Cat#201069
Maxpar® Cell Staining Buffer	Fluidigm	Cat#201068
Maxpar® PBS	Fluidigm	Cat#201058
EQ Four Element Calibration Beads	Fluidigm	Cat#201078
Bond-Breaker TCEP Solution, Neutral pH	Thermo Fisher	Cat#77720
PFA	EMC	50-980-487
Osmium tetroxide	ACROS ORGANICS	319010050
Recombinant SARS-CoV-2 receptor binding domain (RBD)	Krammer Laboratory at the Icahn School of Medicine at Mount Sinai	https://labs.icahn.mssm.edu/krammerlab/reagents/
Recombinant SARS-CoV-2 spike protein (S)	Krammer Laboratory at the Icahn School of Medicine at Mount Sinai	https://labs.icahn.mssm.edu/krammerlab/reagents/
SIGMAFAST™ OPD (*o*-Phenylenediamine dihydrochloride)	Sigma-Aldrich	Cat#P9187
3-molar hydrochloric acid	Thermo Fisher Scientific	Cat#S25856
Tween 20	Fisher Bioreagents	Cat#BP337-100
Non-fat dry milk Omniblok	AmericanBio	Cat#AB10109-01000
Bovine Serum Albumin Fraction V	Roche	Cat#10735078001
Protein A conjugated magnetic beads	Invitrogen	Cat#10008D
Protein G conjugated magnetic beads	Invitrogen	Cat#10009D
T4 ligase	New England Biolabs	Cat#M0202S
Phusion DNA Polymerase	New England Biolabs	Cat# M0530L
Urea	Sigma-Aldrich	
Ammonium Bicarbonate	Sigma-Aldrich	09830-1KG
Iodoacetamide	Sigma-Aldrich	I1149-25G
Dithiothreitol	Sigma-Aldrich	D9779-10G
LC/MS grade Formic Acid	Thermo Scientific	A117-50
Perchloric Acid	Sigma-Aldrich	311421-50ML
1-Propanol	Sigma-Aldrich	34871-1L
Sera-Mag Speed Beads 65	Sigma-Aldrich	65152105050250
Sera-Mag Speed Beads 45	Sigma-Aldrich	45152105050250
HPLC grade Water	Fisher chemical	W5-4
LC/MS grade Water	Fisher chemical	W6-1
LC/MS grade Acetonitrile	Fisher chemical	A955-1
HPLC grade Methanol	Fisher chemical	A452-4
LC/MS grade Methanol	Fisher chemical	A456-4
LC/MS grade Isopropanol	Fisher chemical	A461-1
Sequence grade Porcine Trypsin	Promega	V5117
K562 Cell Line Tryptic Peptide Mixture Standard 100 μg	Promega	V6951
Trifluoroacetic acid	Sigma-Aldrich	T6508-100ML
Ambion Nuclease-Free Water	Invitrogen	Cat#AM9937
Recombinant human IFNa	R&D	Cat#11101-2
Recombinant human IFNb	Peprotech	Cat#300–02BC
Recombinant human IFNw	Peprotech	Cat#300-02J
Sulfo-NHS	ThermoScientific	Cat#A39269
EDC	ThermoScientific	Cat#77149

**Critical commercial assays**		

Quick-DNA/RNA Pathogen MagBead	Zymo Research	R2146
RNase-Free DNase Set	Qiagen	79254
NEBNext Ultra II Directional RNA Library Prep Kit for Illumina	New England Biolabs	E7760
AMPure XP Beads	Beckman-Coulter	A63882
Quick-RNA MagBead Kit	Zymo Research	R2133
SMART-Seq v4 Ultra Low Input RNA Kit for Sequencing	Takara Bio	634894
Nextera XT DNA Library Preparation Kit	Illumina	FC-131-1096
DNA Prep, Tagmentation	Illumina	20018705
Chemagic Blood 400 (96) kit	Perkin Elmer	CMG-1091
Global Diversity Array (GDA)	Illumina	20031810
Covaris E210	Covaris, LLC.	10521
T7 Select 10-3b Cloning kit	EMD Millipore	EMD Millipore
AMPure XP Beads	Beckman Coulter	Cat#A63881
Olink Target 96 Inflammation Reagent Kit	Olink Proteomics	Cat#95302, Lot#B02101

**Deposited data**		

IMPACC cohort data files	ImmPort Database	SDY1760
IMPACC Genomic and transcriptomics data	dbGAP	phs002686.v1.p1
Table S1. Demographics and clinical characteristics of cohort participants at baseline (N = 540), related to Figure 1, Table S2. Regions of seroreactivity within the SARS-CoV-2 proteome (antibody titers: peptides) modules and association, related to Figure 2, Table S3. Sex, age, and trajectory group distribution of participants with and without auto-Abs against IFNs, related to Figure 2, Table S4. Olink modules and association with clinical outcome, related to Figure 3, Table S5. Targeted proteomics modules and association with clinical outcome, related to Figure 3, Table S6. Global proteomics modules and association with clinical outcome, related to Figure 3, Table S7. Global metabolomics modules and association with clinical outcome, related to Figure 4, Table S8. Blood CyTOF modules and association with clinical outcome, related to Figure 5, Table S9. PBMC transcriptomics modules and association with clinical outcome, related to Figure 6, Table S10. Genomic associations with COVID-19 hospitalization previously reported by HGI, related to Figure S8, Table S11. Nasal transcriptomics sample module results, related to Figure 6, Table S12. Nasal metagenomics results and association results, related to Figure S10, Table S13. Information on data preparation, related to STAR Methods, Table S14. Number of samples used for WGCNA module creation, along with assay-specific parameters, related to STAR Methods and Figures S1–S13	This paper Mendeley data	Mendeley Data: https://doi.org/10.17632/vcskpv8tjk.1

**Public databases**		

Genome Reference Consortium Human Build 38^72^	Genome Reference Consortium Schneider et al.^72^	GRCh38 https://www.ncbi.nlm.nih.gov/assembly/GCF_000001405.26/
Ensembl release 91^73^	European bioinformatics institute Cunningham et al.^73^	https://www.ebi.ac.uk/about/news/updates-from-data-resources/ensembl-release-91/
SARS-CoV-2 ref. ^74^	GenBank Wu et al.^74^	NCBI strain GenBank: MN908947.3 https://www.ncbi.nlm.nih.gov/nuccore/MN908947.3
SARS-CoV-2 lineages^22^	Phylogenetic Assignment of Named Global Outbreak (PANGO) Rambaut et al.^22^	https://cov-lineages.org/
KEGG Pathway^41^	Kyoto Encyclopedia of Genes and Genomes Kanehisa et al.^41^	https://www.genome.jp/kegg/
MSigDB Hallmark^39^	Gene Set Enrichment Analysis Molecular Signatures Database Liberzon et al.^39^	https://www.gsea-msigdb.org/gsea/msigdb/
Reactome^40^	Reactome Pathways Database Gillespie et al.^40^	https://reactome.org/
STRINGdb^42^	STRING Database Szklarczyk et al.^42^	https://string-db.org/
ImmuneXpresso^25^	ImmuneXpresso Knowledgebase Kveler et al.^25^	http://immuneexpresso.org/immport-immunexpresso/public/immunexpresso/search
COVID-19 Drug and Gene Set Library^75^	Kuleshov et al.^75^	https://maayanlab.cloud/covid19/

**Experimental models: Cell lines**		

Expi293F cells	Thermo Fisher	Cat#A14528

**Oligonucleotides**		

2019-nCOV_N1-F GAC CCC AAA ATC AGC GAA AT	Integrated DNA technologies	Cat#10006713
2019-nCOV_N1-R TCT GGT TAC TGC CAG TTG AAT CTG	Integrated DNA technologies	Cat#10006713
2019-nCOV_N1-P ACC CCG CAT TAC GTT TGG TGG ACC	Integrated DNA technologies	Cat#10006713
2019-nCOV_N2-F TTA CAA ACA TTG GCC GCA AA	Integrated DNA technologies	Cat#10006713
2019-nCOV_N2-R GCG CGA CAT TCC GAA GAA	Integrated DNA technologies	Cat#10006713
2019-nCOV_N2-P ACA ATT TGC CCC CAG CGC TTC AG	Integrated DNA technologies	Cat#10006713
RP-F AGA TTT GGA CCT GCG AGC G	Integrated DNA technologies	Cat#10006713
RP-R GAG CGG CTG TCT CCA CAA GT	Integrated DNA technologies	Cat#10006713
RP-P TTC TGA CCT GAA GGC TCT GCG CG	Integrated DNA technologies	Cat#10006713
SARS-CoV-2 tilling oligonucleotides for whole genome amplification^76^	Gonzalez-Reiche et al.^76^	https://doi.org/10.1126/science.abc1917

**Recombinant DNA**		

Vector pCAGGS Containing the SARS-Related Coronavirus 2, Wuhan-Hu-1 Spike Glycoprotein Gene (soluble, stabilized)	BEI Resources	Cat#NR-52394
Vector pCAGGS Containing the SARS-Related Coronavirus 2, Wuhan-Hu-1 Spike Glycoprotein Receptor Binding Domain (RBD)	BEI Resources	Cat#NR-52309
Human Coronavirus Synthetic DNA	Twist Bioscience	https://www.twistbioscience.com

**Software and algorithms**		

CZID Pipeline	Chan Zuckerberg Initiative	www.czid.org
bcl2fastq v2.20.0.422	Illumina	https://support.illumina.com/sequencing/sequencing_software/bcl2fastq-conversion-software.html
FastQC_v0.11.5^77^	Andrew S.	https://github.com/s-andrews/FastQC
STARv2.4.2a^78^	Dobin et al.^78^	https://github.com/alexdobin/STAR
Qualimap^79^	Okonechnikov et al.^79^	http://qualimap.conesalab.org
Cutadapt_v3.7^80^	Martin, Marcel https://doi.org/10.14806/ej.17.1.200	https://cutadapt.readthedocs.io/en/stable/
Preseq_v3.1.1^81^	Daley and Smith^81^	https://github.com/smithlabcode/preseq
MultiQC^82^	Ewels et al.^82^	https://multiqc.info
WGCNA R package (version 1.69–81)^13^	Langfelder, Peter, and Steve Horvath. "WGCNA: an R package for weighted correlation network analysis." BMC bioinformatics 9, no. 1 (2008): 1–13.	https://cran.r-project.org/web/packages/WGCNA/index.html
lme4 R package (version 1.1–27.1)^83^	Bates, Douglas, Deepayan Sarkar, Maintainer Douglas Bates, and L. Matrix. "The lme4 package." R package version 2, no. 1 (2007): 74	https://cran.r-project.org/web/packages/lme4/index.html
Ordinal R package (version 2019.12–10)^84^	Christensen, Rune Haubo B. "Cumulative link models for ordinal regression with the R package ordinal." Submitted in J. Stat. Software 35 (2018).	https://cran.r-project.org/web/packages/ordinal/index.html
gamm4 R package (version 0.2–6)^85^	Wood, Simon, Fabian Scheipl, and Maintainer Simon Wood. "Package ‘gamm4’." Am Stat 45, no. 339 (2017): 0–2.	https://cran.r-project.org/web/packages/gamm4/index.html
ComplexHeatmap R package (version 2.6.2)^86^	Gu Z, Eils R, Schlesner M (2016). “Complex heatmaps reveal patterns and correlations in multidimensional genomic data.” Bioinformatics.	https://www.bioconductor.org/packages/release/bioc/html/ComplexHeatmap.html
pvca R package (version 1.30.0)^87^	Bushel P.^88^ pvca: Principal Variance Component Analysis (PVCA). R package version 1.34.0.	https://www.bioconductor.org/packages/release/bioc/html/pvca.html
SamTools (v1.1, 1.2, 1.4)^89^	Danecek et al.^90^	http://samtools.sourceforge.net RRIF:SCR_002105
Trimmomatic-toolkit (v0.36.5)^91^	Bolger, A. M., Lohse, M., & Usadel, B.^91^; Trimmomatic: A flexible trimmer for Illumina Sequence Data. Bioinformatics, btu170.	RRID:SCR_011848
HTSeq-count (v0.4.1)^92^	Anders et al.^92^15	RRID:SCR_011867
Picard (v1.134)^93^	Broad Institute	RRID:SCR_006525
FASTQC (v0.11.3)^77^	Babraham Institute	RRID:SCR_014583
Data.table R package 1.14.2^94^	Dowle, M et al.^94^ Data.table R package version 1.14.2	https://cran.r-project.org/web/packages/data.table/index.html
DT R package 0.21^95^	Xue, Yihui et al.^95^; DT: A Wrapper of the JavaScript Library DataTables R package version 0.21	https://cran.r-project.org/web/packages/DT/index.html
E1071 R package^96^	Meyer, D et al.,^97^ e1071: Misc Functions of the Dept of Statistics, Probability Theory Group. R package version 1.7–9.	https://cran.r-project.org/web/packages/e1071/index.html
Metabolon Laboratory Information Management System (LIMS)	Metabolon	https://www.metabolon.com/
MassFragment Application Manager	Waters	Waters MassLynx v.4.1 Waters Corp Milford, USA https://www.waters.com/waters/en_US/MassFragment-/nav.htm?locale=/&cid=1000943
MetaboAnalyst 5.0^98^	MetaboAnalyst	https://www.metaboanalyst.ca/
Cytutils R package v0.1.0^97^	Amir et al.^97^	https://github.com/ismms-himc/cytutils
Fluidigm software-acquisition, normalization, concatenation v7.0.8493	Fluidigm	https://www.fluidigm.com/products-services/software
Cytobank^99^	Beckman Coulter	https://premium.cytobank.org
Prism 9	GraphPad	https://www.graphpad.com/
R v4.0.2	The Comprehensive R Archive Network	https://cran.r-project.org/
FLASH v1.2.11^100^	Magoc and Salzberg^100^	https://ccb.jhu.edu/software/FLASH/
Bowtie2 v2.2.7^101^	Langmead and Salzberg^102^	http://bowtie-bio.sourceforge.net/bowtie2/index.shtml
NCBI BLAST v2.11.0^103^	Altschul et al.^103^	https://blast.ncbi.nlm.nih.gov/Blast.cgi
CD-HIT^104^^,^^105^	Li and Godzik^104^ Fu et al.^105^	http://weizhong-lab.ucsd.edu/cd-hit/download.php
COVID_pipe (https://github.com/mjsull/COVID_pipe)^76^	Gonzalez-Reiche et al.^76^	https://doi.org/10.5281/zenodo.3775031
Minimap2 v2.17-r941^106^	Li^106^	https://doi.org/10.1093/bioinformatics/bty191
Shovill v1.1.0^107^	Kwong, Gladman and Goncalves da Silva	https://github.com/tseemann/shovill
Pilon v1.24^108^	Walker et al.^1^^37^	http://doi.org/10.1371/journal.pone.0112963
Canu v2.2^109^	Koren et al.^109^	http://doi.org/10.1101/gr.215087.116
Prokka v1.14.6^110^	Seeman^110^	http://doi.org/10.1093/bioinformatics/btu153
Seqkit v2.1.0^111^	Shen et al.^111^	http://doi.org/10.1371/journal.pone.0163962
Kraken2 v2.1.2^112^	Wood et al.^112^	https://doi.org/10.1186/s13059-019-1891-0
Skyline v.21.2.1.377^113^	MacCossLab	http://skyline.ms
LabSolutions v.5.97	Shimadzu Scientific Instruments	https://www.ssi.shimadzu.com/products/informatics/labsolutions.html
Perseus^114^	Tyanova et al.^1^^46^	https://maxquant.org/perseus//
Fluidigm Real-Time PCR Analysis v4.7.1	Fluidigm	https://www.fluidigm.com/products-services/software
Olink NPX Manager v3.3.2.434	Olink Proteomics	https://www.olink.com/products-services/data-analysis-products/npx-manager/
Nextstrain v. 3.2.0^115^	Hadfield et al.^115^	https://github.com/nextstrain/ncov
Nextclade v. 1.11.0^115^	Aksamentov et al.^116^	https://doi.org/10.21105/joss.03773
Pangolin v. 1.11.0^21^	O’Toole et al.^21^	https://doi.org/10.1093/ve/veab064
Baltic v.0.1.6^117^	Dudas^117^	https://github.com/evogytis/baltic
IQ-TREE2 v.1.6.12^118^	Minh et al.^118^; Hoang et al.^119^	https://doi.org/10.1093/molbev/msaa015, https://doi.org/10.1093/molbev/msx281

**Other**		

Turbovap Evaporator	Biotage	Zymark TurboVap Cat#Z-TLVE
Waters Acquity UPLC	Waters	Waters Acquity
BEH C18 columns	Waters	Waters Acquity 2.1 × 100 mm, 1.7 μm columns
Q-Exactive with Orbitrap mass analyzer	Thermo Scientific	Cat#IQLAAEGAAPFALGMBDK
HILIC columns	Waters UPLC	Waters UPLC BEH Amide 2.1 × 150 mm, 1.7 um
Hamilton MicroLab Star Liquid Handling Robotic System	Hamilton Company	https://www.hamiltoncompany.com/automated-liquid-handling/platforms/microlab-star
Geno/Grinder 2000	SPEX Sample Prep	Geno/Grinder 2000
NovaSeq 6000	Illumina	N/A
0.45μm filter plates	Arctic White	AWFP-F20022
1000 μl Pipette Tips	Opentrons	991–00005
300 μl Pipette Tips	Opentrons	991–00008
20 μl Pipette Tips	Opentrons	999–00014
10 μl Pipette Tips	Opentrons	999–00014
20 μl Pipette Tips	Axygen	T-20-R-S
200 μl Pipette Tips	Axygen	T-200-C-L-R-S
Sealing tape 96-well Plates	4titude	4ti-0581
25mL Reservoir	Argos	B3125-100
4-well Reservoir	Axygen	RES-MW4-HP
12-well Reservoir	Axygen	RES16MC-12-N
0.5 mL 96-well Plates	VWR	76210–520
0.8 mL 96-well Plates	VWR	76210–524
MACROSpin C18 plates	The Nest Group Inc.	SNS SS18VL
EvoTip	Evosep	EV2008
PepSep LC 8cm column	Pepsep	PSC-8-150-15-UHP-nC - 8 cm nanoConnect column
Shimadzu LC column	Shimadzu	227-32100-02
Captive Spray Emitter (ZDV) 20 μm	Bruker	1865710
Combitips® advanced, Eppendorf Quality™, 0.5 mL	Eppendorf	0030089421
Combitips® advanced, Eppendorf Quality™, 2.5 mL	Eppendorf	0030089448
Combitips® advanced, Eppendorf Quality™, 5 mL	Eppendorf	0030089448
Combitips® advanced, Eppendorf Quality™, 10 mL	Eppendorf	0030089464
EvoSep One	Evosep	EV-1000
Thermomixer	Eppendorf	N/A
timsTOF Pro	Bruker Daltonik GmBH	N/A
Column Oven Sonation PRSO-V2	Sonication lab solutions	PRSO-V2
Nexera Mikros	Shimadzu Scientific Instruments	N/A
LCMS 8060	Shimadzu Scientific Instruments	N/A
Fluidigm Dynamic Array 96.96 GE IFC	Fluidigm	Cat#BMK-*M*-96.96
Fluidigm Ctril Line Fluid,150ul	Fluidigm	Cat#89000021
Magnetic COOH Beads Region 34	BioRad	Cat#MC10034-01
Magnetic COOH Beads Region 43	BioRad	Cat#MC10043-01
Magnetic COOH Beads Region 63	BioRad	Cat#MC10063-01
Amine coupling kit	BioRad	Cat#171406001

### Resource availability

All requests for information regarding reagents and resources should be directed to the lead contact and will be fulfilled by the lead contact or corresponding authors.

#### Lead contact

Further information and requests for resources and reagents should be directed to and will be fulfilled by Dr. Steven Kleinstein (steven.kleinstein@yale.edu).

#### Materials availability

This study did not generate new unique reagents.

### Experimental model and study subject details

#### IMPACC cohort characteristics

The IMPACC cohort enrolled participants from 20 hospitals affiliated with 15 geographically distributed academic institutions across the U.S. Eligible participants were patients hospitalized with symptoms or signs consistent with COVID-19, which had SARS-CoV-2 infection confirmed by RT-PCR to remain in the study. The detailed study design, schedule for clinical data, biological sample collection and demographic information about the participants were previously described.^10^^,^^11^ Briefly, detailed clinical assessments and nasal, blood, and endotracheal aspirates (intubated participants only) were collected within 72h of hospitalization (visit 1) and on days 4, 7, 14, 21, 28 after hospital admission (visits 2–6, respectively). If a participant required escalation of care or was re-admitted to the hospital prior to Day 28, additional samples were collected within 24 and 96 h of care escalation or readmission. If participants were discharged prior to day 14 or 28, attempts were made to collect limited clinical information and biologic samples on days 14 and/or 28 in outpatients. Disease severity was assessed using a 7-point ordinal scale based on degree of respiratory illness,^10^ modified from Beigel et al.

#### Cell culture conditions

Expi293F cells (Gibco #A14527) used for antibody titer assay were cultured in Expi293 Expression Medium before transfection as described previously^121^ and in SARS-CoV-2 recombinant RBD and spike proteins method section.

### Method details

#### Sample processing and batch randomization

Biological sample collection and processing followed a standard protocol utilized by every participating academic institution. The complete IMPACC sample processing protocol was published previously.^10^ To mitigate potential batching effects, a randomization procedure was developed to help ensure that longitudinal samples from the same individuals were run on the same plates and were randomly distributed across the plates. We stratified this randomization by disease severity (mild/moderate versus severe) and age (younger versus older) with the representation of these strata across plates. In addition, we verified that race, ethnicity, gender, and site were well represented across the plates.

#### Nasal viral PCR, host transcriptomics, and metagenomics

##### RNA preparation

Inferior nasal turbinate swabs were collected and placed in 1mL of Zymo-DNA/RNA shield reagent (Zymo Research). RNA was extracted from 250 μL of sample and eluted into a volume of 50ul using the KingFisher Flex sample purification system (ThermoFisher) and the quick DNA-RNA MagBead kit (Zymo Research) following the manufacturer’s instructions. Each sample was extracted twice in parallel. The 2 eluted RNA samples were pooled and aliquoted into 20 μL aliquots using a Rainin Liquidator 96 pipettor for downstream RT-qPCR, RNA-sequencing, and viral sequencing.

#### RealTime quantitative polymerase chain reaction

Master mixes containing nuclease-Free water, combined primer/probe mixes, and One-Step RT-qPCR ToughMix (Quantabio) were prepared on ice, and 15 μL was dispensed in each well of a 384-reaction plate (Thermofisher) CoV-2 was quantitated using the CDC qRT-PCR assay (primers and probes from IDT). Briefly, this comprises two reactions targeting the CoV-2 nucleocapsid gene (N1 and N2) and one reaction targeting *RPP30* (RP). Each batch included positive controls of plasmids containing N1/N2 and RP target sequence (2019-nCoV_N_Positive Control and Hs_RPP30 Positive Control, IDT) to allow quantitation of each transcript. Primer/probe sequences were: 2019-nCOV_N1-F GAC CCC AAA ATC AGC GAA AT, 2019-nCOV_N1-R TCT GGT TAC TGC CAG TTG AAT CTG, 2019-nCOV_N1-P ACC CCG CAT TAC GTT TGG TGG ACC, 2019-nCOV_N2-F TTA CAA ACA TTG GCC GCA AA, 2019-nCOV_N2-R GCG CGA CAT TCC GAA GAA, 2019-nCOV_N2-P ACA ATT TGC CCC CAG CGC TTC AG, RP-F AGA TTT GGA CCT GCG AGC G, RP-R GAG CGG CTG TCT CCA CAA GT and RP-P TTC TGA CCT GAA GGC TCT GCG CG. After RNA extracts were gently vortexed and added 5 μL per sample. Plates were centrifuged for 30 s at 500 × g, 4C. Quantitative polymerase chain reaction was performed using a Quantstudio5 (Thermo Fisher) with cycling conditions:1 cycle 10 min at 50°C, followed by 3 min at 95°C, 45 cycles 3 s at 95°C, followed by 30 s at 55.0°C.

#### RNA-sequencing cDNA library production

From each nasal RNA sample, 10ul was aliquoted to a library construction plate using the Perkin Elmer Janus Workstation (Perkin Elmer, Janus II). Ribosomal depletion, cDNA synthesis, and library construction steps were performed using the Total Stranded RNA Prep with Ribo-Zero Plus kit, following the manufacturer’s instructions (Illumina). All steps were automated on the Perkin Elmer Sciclone NGSx Workstation to reduce batch-to-batch variability and increase sample throughput. Final cDNA libraries were quantified using the Quant-it dsDNA High Sensitivity assay, and library insert size distribution was checked using a fragment analyzer (Advanced Analytical; kit ID DNF474). Samples, where adapter dimers constituted more than 4% of the electropherogram area, were failed before sequencing. Technical controls (K562, Thermo Fisher Scientific, cat# AM7832) were compared to expected results to ensure that batch to batch variability was minimized. Successful libraries were normalized to 10nM for sequencing.

#### RNA-sequencing clustering and sequencing

Barcoded libraries were pooled using liquid handling robotics prior to loading. Massively parallel sequencing-by-synthesis with fluorescently labeled reversibly terminating nucleotides was carried out on the NovaSeq 6000 sequencer using S4 flowcells with a target depth of 50 million 100 base-pair paired-end reads per sample (25 million read pairs).

#### Nasal viral genome sequencing and assembly

For viral genome sequencing,^76^ cDNA synthesis was performed with random hexamers and ProtoScript II (New England Biolabs, E6560) starting from 7 μL of total RNA extracted from clinical specimens. The SARS-CoV-2 genome was then amplified with Q5 Hot Start High-Fidelity DNA polymerase (New England Biolabs, cat. M0493) using two sets of custom-designed tiling primers generating overlapping amplicons of ∼1.5 and 2 kb. The PCR amplification parameters were: 1 min at 98C, 35 cycles of 15 s at 98C and 5 min at 63C, and final extension for 10 min at 65C. After equivolume pooling of the amplicons and cleanup with 1.8X volume of Ampure XT beads, libraries were prepared using the Nextera XT DNA Sample Preparation kit (Illumina, FC-131-1096), followed by paired-end sequencing (2 × 150nt) on the Illumina MiSeq platform. A custom reference-based analysis pipeline, https://github.com/mjsull/COVID_pipe, was used to assemble SARS-CoV-2 genomes. Whole-genome viral amplification was initially conducted on 1,154 nasopharyngeal swab samples collected from 474 participants, of which 531 samples had a detectable PCR band to attempt sequencing. Of these samples, 316 yielded complete viral genomes from 221 participants.

#### Antibody correlates: titers

##### Enzyme-linked immunosorbent assay (ELISA)

Antibodies antibody levels against the recombinant receptor-binding domain (RBD) and full-length spike were measured using a research-grade ELISA as described.^122^^,^^123^ Briefly, samples were heat-inactivated at 56°C for 1 h. 96-well plates (Thermo Fisher Lot # 4199147) were coated with 50 μL/well of RBD or spike proteins at 2 μg/mL concentration in phosphate-buffered saline (PBS; Gibco lot # 2388102) and incubated overnight at 4°C. Plates were washed 3× in an automatic plate washer (BioTek) with PBS 0.01% Tween 20 (Fisher Scientific, Cat#BP337-100, TPBS) and blocked for 1 h with 200 μL/well of 3% non-fat dry milk (Cat#AB10109-01000) prepared in TPBS. Serum samples were serially diluted (3-fold starting at 1:80 dilution) in 1% non-fat dry milk in TPBS. The blocking solution was removed, and 100 μL/well of serially diluted samples were added to the plates and incubated for 2h at 20°C. Plates were washed 3× with TPBS, and 50 μL/well of the corresponding secondary antibody, prepared in 1% non-fat dry milk in TPBS, were added for 1h at RT: Anti-human IgG (Fc specific)-Peroxidase antibody produced in goat (Sigma-Aldrich Cat#A0170); Goat anti-human IgM-HRP (SouthernBiotech Cat#2020–05); Anti-human IgA (α-chain specific)-Peroxidase antibody produced in goat (Sigma-Aldrich Cat#A0295). Plates were washed 3× with TPBS, and 100 μL/well of peroxidase substrate (SigmaFAST *o*-phenylenediamine dihydrochloride, Sigma-Aldrich Cat#P9187) were added for 10 min 50 μL/well of 3M hydrochloric acid (HCl, Thermo Fisher Scientific, Cat#S25856) was added to stop the reaction. Optical density (OD) was measured in a Synergy 4 (BioTek) plate reader at 490 nm. The area under the curve was calculated, considering 0.15 OD as the cutoff. Data were analyzed using Graphpad Prism 9.

#### SARS-CoV-2 recombinant RBD and spike proteins

Recombinant RBD and spike proteins of SARS-CoV-2 were generated and expressed as previously described.^124^ Briefly, constructs consisted of mammalian-cell codon-optimized nucleotide sequences for RBD (amino acids 319–541), including a signal peptide and hexahistidine tag, or the soluble version of the spike protein (amino acids 1–1,213) with a signal peptide, C-terminal thrombin cleavage site, T4 fold-on trimerization domain, and hexahistidine tag. These sequences were cloned into the mammalian expression vector pCAGGS. The nucleotide sequence of the spike protein was additionally modified to remove the polybasic cleavage site, and two stabilizing mutations (K986P and V987P) were introduced. The expression plasmids are available at BEI Resources Repository (https://www.beiresources.org/). Recombinant proteins were produced in Expi293F cells (Thermo Fisher) using the ExpiFectamine 293 Transfection Kit (Thermo Fisher) according to the manufacturer’s instructions. Expi293F cells (Gibco #A14527) were cultured in Expi293 Expression Medium before transfection as described previously^121^ and in SARS-CoV-2 recombinant RBD and spike proteins method section. Expi293F cells were not authenticated and tested negative for mycoplasma. Proteins were purified by gravity flow using Ni-NTA Agarose (Qiagen) and concentrated in Amicon centrifugal units (EMD Millipore). Purified proteins were analyzed by reducing sodium dodecyl sulfate–polyacrylamide gel electrophoresis (SDS–PAGE), and correct folding was confirmed by performing ELISAs with RBD-specific monoclonal antibody CR3022.

#### Autoantibody screening assay

Samples were screened for autoantibodies^2^ against type I IFNs in a multiplex, particle-based assay, in which differentially fluorescing magnetic beads were covalently coupled to recombinant human proteins (2.5 μg/reaction). Beads were combined and incubated with 1:100 diluted serum samples for 30 min. Each sample was tested once with a random assortment in each plate tested in duplicate to ensure minimal intra-assay variability. Beads were washed then incubated with PE-labeled goat anti-human IgG (1ug/mL) for an additional 30 min. Beads were washed again and run on a BioPlex X200 instrument in a multiplex assay. Participant samples with a fluorescence intensity greater than 3 standard deviations above the mean of 1099 healthy controls at the earliest timepoint received (>1310 FI for IFNa; >386 FI for IFNb; >1387 for IFNw) were tested for blocking activity in the pSTAT1 functional assay.

#### pSTAT1 functional assay

The blocking activity of anti-IFN-containing serum was determined by assessing STAT1 phosphorylation in healthy control cells following stimulation with the appropriate cytokine in the presence of 10% healthy control or participant serum.^2^ Surface-stained healthy control PBMCs (350,000/reaction) were cultured in serum-free RPMI medium with 10% healthy control (pooled human AB serum) or participant serum and were either left unstimulated or stimulated with IFNa, IFNw, or IFNb (10 ng/mL) for 15 min at 37°C. Cells were fixed, permeabilized, and stained for intranuclear phopsho-STAT1 (Y701). Cells were acquired on a BD LSR Fortessa cytometer with gating on CD14^+^ monocytes and analyzed with FlowJo software. A stimulation index was calculated for each sample by dividing the geometric mean fluorescence for the pSTAT1 channel for each stimulated condition by that of the unstimulated condition. The stimulation index for each cytokine was then normalized to that of the healthy control serum from the same assay, generating a normalized stimulation index (where >65% pSTAT1 = NOT blocking; 20–65% pSTAT1 = partially blocking; <20% pSTAT1 = blocking). Statistical analyses were performed using Fisher’s exact test of the overall association of autoantibodies among all 5 TGs.

#### Antibody correlates: coronavirus phage display (VirScan)

##### Coronavirus library design and cloning

As described previously,^125^ RefSeq sequences for SARS-CoV-2 (NC_045512), SARS-CoV-1 (NC_004718), and 7 other coronaviruses known to infect humans, including beta coronavirus England 1 (NC_038294), HuCoV 229E (NC_002645), HuCoV HKU1 (NC_006577), HuCoV NL63 (NC_005831), HuCoV OC43 (NC_006213), Infectious Bronchitis virus (NC_001451), and MERS CoV (NC_019843) were downloaded from the National Center of Biotechnology Information (NCBI). All open reading frames for each virus were divided into sequences of 38mer peptides, with consecutive peptides sharing a 19 amino acid overlap. All peptide sequences were collapsed on 99% amino acid sequence identity using cd-hit. A subsequent patch of spike peptides was added to the library to account for variation in the spike protein. All spike protein sequences present in NCBI databases, including the SARS-CoV-2 allele used to generate the initial library, were downloaded (as of 10/02/2020), aligned, and divided into 38mers with a 19 amino acid overlap as already described. Only peptides with >3 amino acid differences (<92% sequence similarity) were retained and added to the library. The combined set of peptide sequences was converted to DNA sequences using an R language script, randomizing codon selection. Twenty-one (21) nucleotide 5′ linker sequences, as well as a nucleic acid sequence encoding an FLAG tag (DYKDDDDK) at the 3′ end of each oligonucleotide sequence, were added. The final oligonucleotide sequences were 159 nucleotides in length, outputted to a FASTA file, and sent to Twist Biosciences for synthesis. A single vial of 10pmoles of lyophilized DNA was received from Twist. The lyophilized oligonucleotides were resuspended in 10mM Tris/1mM EDTA (TE) to a final concentration of 0.2nM and PCR amplified for cloning into T7 phage vector arms (Novagen/EMD Millipore, Inc).

#### Preparation of phage libraries

Phage libraries were prepared and amplified fresh from packaging reactions. To prepare phage libraries, a 300 mL culture of *E*. *coli* BLT5403 was incubated at 37°C with shaking until the mid-log phase, defined as OD_600_ = 0.4. The culture was then inoculated at a multiplicity of infection (MOI) of 0.001 and incubated at 37°C for 2.5 h or until complete lysis was observed, after which 5M NaCl was added to the lysate for stabilization, and the lysate was placed on ice. The lysate was then spun at 8000 g for 15 min to pellet cellular debris. The phage-containing supernatant was 0.2uM filtered, and 5X PEG/NaCl (PEG-8000 20%, NaCl 2.5 mM) added to a final 1X concentration and incubated overnight at 4°C. The PEG-phage lysate mix was centrifuged for 15 min at 4000 g at 4°C, and the pellet was resuspended in storage media (20 mM Tris-HCl, pH 7.5, 100 mM NaCl, 6 mM MgCl_2_) before 0.22μM filtration. Phage libraries were titered by plaque assay and adjusted to a working concentration of 10^10^ pfu/mL before incubation with participant sera. All VirScan experimental procedures and data analysis are described fully.^10^^,^^125^

#### Serum proximity extension assay (olink)

Study samples were assayed in plate batch layouts following a centralized randomized scheme described above. Three samples (IMPACC_Serum, IMPACC_Plasma, and IMPACC_Plasma_Stim) were used as IMPACC inter-plate references (Reference samples)] in every plate. All samples (participant sera and reference) were subjected to PEA (Olink) multiplex assay Inflammatory panel (Olink Bioscience, Uppsala, Sweden), according to the manufacturer’s instructions. This inflammatory panel included 92 proteins associated with human inflammatory conditions. An incubation master mix containing pairs of oligonucleotide-labeled antibodies to each protein was added to the samples and incubated for 16 h at 4°C. Each protein was targeted with two different epitope-specific antibodies, increasing the assay’s specificity. The presence of the target protein in the sample brought the partner probes in close proximity, allowing the formation of a double-strand oligonucleotide polymerase chain reaction (PCR) target. On the following day, the extension master mix in the sample initiated the specific target sequences to be detected and generated amplicons using PCR in 96 well plates. For the detection of the specific protein, Dynamic array integrated fluidic Circuit (IFC) 96 × 96 chip was primed, loaded with 92 protein-specific primers, and mixed with sample amplicons, including three inter-plate controls (IPS) and three negative controls (NC). Real-time microfluidic qPCR was performed in Biomark (Fluidigm, San Francisco, CA) for the target protein quantification.

#### Plasma global proteomics

Fifty microliters of neat plasma samples were diluted with 450 μL of water, and 25 μL of perchloric acid was added.^126^ After vigorous agitation, the suspension was kept at −20°C for 15 min, then centrifuged for 60 min (4°C, 3200 ×g). 390 μL of the supernatant was mixed with 40 μL of 1% trifluoroacetic acid and loaded onto a μSPE HLB plate, previously conditioned once with 300 μL methanol and twice with 500 μL of 0.1% trifluoroacetic acid. Proteins were eluted from the μSPE HLB plate with 100 μL of 90% acetonitrile and 0.1% trifluoroacetic acid. After elution, the samples were dried with a Speedvac, resuspended with 35 μL of 50 mM ammonium bicarbonate, and digested with 10 μL trypsin (500 ng) overnight at 37°C. Digestion was stopped by the addition of 5 μL 10% formic acid. The samples were stored at −80°C before LC/MS analysis. Two microliters of tryptic peptides were loaded onto Evotips and analyzed using an Evosep ONE liquid chromatography system (EVOSEP, Odense, Denmark) connected to a timsTOF Pro mass spectrometer (Bruker Daltonics, Billerica, MA, USA). The Evosep ONE was set to 60 samples per day, and the mass spectrometer was operated in DDA-PASEF mode. DDA-PASEF parameters were set as follows: *m*/*z* range 100–1700, the mobility (1/K0) range was set to 0.70–1.45 Vs./cm2, and the accumulation time was set to 100 ms.

#### Plasma targeted proteomics

All chemicals and reagents were purchased at the highest purities available. Solvents used in this study were LC/MS grade and were purchased from Fisher Chemicals (Thermo Fisher Scientific). Briefly, a volume of 10 μL of 10-fold diluted plasma was mixed with 60 μL of urea buffer (8M urea in 50 mM ammonium bicarbonate, Sigma Aldrich) and 15 μL of dithiothreitol buffer (DTT, 50 mM in urea buffer, Sigma Aldrich) before incubated 30 min on a thermomixer (800 rpm, room temperature). The samples were alkylated using iodoacetamide buffer (IAA, 375 mM in urea buffer, Sigma Aldrich) and incubated for 30 min (800 rpm, room temperature, and dark). A volume of 10 μL of DTT buffer was added to quench the alkylation. The samples were transferred to the SP3 beads mixture (Sera-Mag SpeedBeads, 1:1 v/v, GE Healthcare) previously washed with HPLC water (scale 1:10 protein to beads). Then a volume of 150 μL of absolute ethanol (Supelco) was added, and the mix was incubated for 15 min on a thermomixer (1,000 rpm at room temperature). The samples were placed on the magnetic rack, and the clear supernatant was removed. The beads were washed in three cycles in 200 μL of 80% ethanol. After the final washing step, the samples were trypsinized using 100 μL of trypsin buffer (Promega, 20 μg/mL in 50 mM ammonium bicarbonate) and placed on a thermomixer (1,000 rpm, 2 h, 37°). After digestion, the samples were centrifuged to pulldown the liquid and placed on a magnetic rack to collect the supernatant and were then acidified with 2% v/v formic acid in HPLC water (Sigma Aldrich). The C18 cleanup was performed using a 96-well MACROSPIN C18 plate (TARGA, The NestGroup Inc.), and the tryptic peptides were eluted off the C18 particles using 40% ACN/0.1% FA. The samples were dried and stored at −20°C until LC/MS analysis.^127^ The samples were analyzed using an LC system (Nexera Mikros, Shimadzu) equipped with a Capillary C18 column (0.2 × 100mm, 2.7um particle diameter, Shimadzu) coupled online to an 8060 triple quadrupole mass spectrometer instrument (Shimadzu). From each sample, 1 μg peptide quantity was separated using a non-linear gradient over a 15-min run time operated at 10 μL/min (5% solvent B for 0.2 min; 5 to 40%B for 10.3 min; 85%B for 1.5 min and 5% for 3 min). The final scheduling method was performed using the following parameters: 1.2 s of maximum loop time with minimum dwell time of 2 msec and pause time of 1 msec, Q1 and Q3 resolution set at the ‘unit’ level.

#### Plasma global metabolomics

Plasma metabolite profiling was conducted by Metabolon using in-house standards.^128^^,^^129^ The samples were divided into randomized sample batches, extracted, and prepared for analysis using Metabolon’s solvent extraction method (Evans, 2008). Recovery standards were added to the first step in the extraction process to ensure proper quality control. Protein was removed by methanol precipitation under vigorous shaking for 2 min (Glen Mills GenoGrinder 2000) and then by centrifugation. The supernatants were divided into five fractions: two for analysis by two separate reverse phases (RP)/UPLC-MS/MS methods with positive ion mode electrospray ionization (ESI); one for analysis by RP/UPLC-MS/MS with negative ion mode ESI; one for analysis by HILIC/UPLC-MS/MS with negative ion mode ESI; and one sample was reserved for backup analysis using Waters ACQUITY ultra-performance liquid chromatography (UPLC) and a Thermo Scientific Q-Exactive high resolution/accurate mass spectrometer interfaced with a heated electrospray ionization (HESI-II) source and Orbitrap mass analyzer operated at 35,000 mass resolution. Metabolites were identified by comparison to Metabolon library entries of standard metabolites^128^ based on three criteria: retention index (RI) within a narrow RI window of the proposed identification; accurate mass match to the library ±10 ppm; and the MS/MS forward and reverse scores between the experimental data and authentic standards. Compounds were categorized according to reporting standards set by the Chemical Analysis Working Group of the Metabolomics Standards Initiative,^130^^,^^131^^,^^132^ and appropriate orthogonal analytical techniques were applied to the metabolite of interest and a chemical reference standard. Metabolites were reported that had their corresponding accurate mass confirmed via MS with retention index, chemical, and composition ID.

#### Blood CyTOF

Samples from a given batch were acquired on the Fluidigm Helios mass cytometer in multiple acquisitions. The PROT-1 fixed whole blood samples were processed in batches of 20 samples. Due to sample quality issues, some samples remained pink or red after the barcoding step; those samples were discarded, and the remaining samples were pooled for the remaining staining steps. After staining was completed, the pooled sample was counted and split into 2–3 subsamples to be frozen as FBS/DMSO samples stored at −80C until the day of acquisition. On the day of acquisition, the Helios instrument was tuned according to the manufacturer’s software standards; if the signal of Tb159 or Tm169 from the Fluidigm Tuning Solution was more than 10% lower than previous days, the process was repeated until the margin was achieved. The final Tuning results were exported as a CSV from the software for the record.

One FBS/DMSO subsample was thawed, washed once with Fluidigm Cell Staining Buffer, and then counted on a Bio-Rad TC20 cell counter. If necessary, the sample was split into subsamples of 2 × 10^6^ cells, centrifuged, and the resulting pellet was left with a minimal overlay of CSB. One CSB subsample was washed twice in MilliQ water, or Fluidigm Cell Acquisition Solution then resuspended to 7–8 x 10^5^/mL in CAS or MilliQ containing a 10-fold dilution of Fluidigm EQ 4-Element normalization beads and acquired on the tuned Helios instrument using either the PSI or SuperSampler for sample introduction. This dilution was chosen to give approximately 250–350 events/sec acquisition rate. The next CSB subsample or FBS/DMSO subsample was processed when the previous sample had less than 1mL of sample remaining. The instrument was cleaned with Fluidigm Wash Solution whenever clogging occurred, or approximately every 2 × 10^6^ cell events were acquired. These cleaning steps resulted in multiple FCS files per pooled sample acquisition. Pooled samples were acquired until a total of 6 × 10^6^ cell events had been collected, or all FBS/DMSO samples were collected, whichever occurred first. This corresponds to an average target event number of 3 × 10^5^ events per original donor subsample.

#### Peripheral blood mononuclear cell transcriptomics

RNA was extracted from cells (2.5 × 10^5^ PBMCs) homogenized in 200 μL of Buffer RLT (Qiagen) and then extracted using the Quick-RNA MagBead Kit (Zymo) with DNase digestion. RNA quality was quantitated using Qubit HS RNA assays and assessed using a Fragment Analyzer (Agilent). Library preps were performed using the SMART-Seq v4 Ultra Low Input RNA Kit (Takara Bio) to synthesize full-length cDNA from an input of 10ng of RNA. After a bead-based clean-up to purify the cDNA, the Nextera XT kit was used to create libraries through a process of tagmentation and fragment amplification and appended with dual-indexed bar codes using the NexteraXT DNA Library Preparation kit (Illumina). Libraries were validated by capillary electrophoresis on a Fragment Analyzer (Agilent), pooled at equimolar concentrations, and sequenced on an Illumina NovaSeq6000 (Emory) at 100 bp, paired-end read length targeting ∼25 million reads per sample. Repeated measures from a group of PBMC samples collected from healthy controls and repeated measures of a subset of IMPACC samples were used across library prep and sequencing batches to assess inter-site batch effects throughout the study. Universal Human References controls were included to assess intra-site batch variation.

#### Genetics

DNA was extracted using the Chemagic 360 system (PerkinElmer Inc), DNA preparations with low yield or fragmented samples (detected on 1% agarose gel) were removed. These samples were genotyped on the Illumina Global Diversity Array per the manufacturer’s protocol [Illumina’s LCG protocol] (support.illumina.com/downloads/infinium-lcg-assay-reference-guide-15023139.html" title = "https://support.illumina.com/downloads/infinium-lcg-assay-reference-guide-15023139.html">https://support.illumina.com/downloads/infinium-lcg-assay-reference-guide-15023139.html). Briefly, genomic DNA was normalized to 200ng in 4 μL 1XTE buffer using pico green quantification. Samples were whole genome amplified for 22 h, fragmented, precipitated, resuspended, and then hybridized to arrays for 18 h. Arrays were then washed, stained, dried, and scanned to produce raw iDat files, from which variants were called using Illumina’s Genome Studio v.2.0.5. Data were then exported in vcf format and converted to plink ped/map format for further analysis.^133^^,^^134^

### Quantification and statistical analysis

#### OMIC-specific preprocessing from raw to computable matrices

##### Nasal host transcriptomics read processing and alignment

Base calls were generated in real-time on the NovaSeq6000 instrument (RTA 3.1.5). Demultiplexed, unaligned BAM files were produced by Picard^93^ ExtractIlluminaBarcodes, and IlluminaBasecallsToSam were converted to FASTQ format using SamTools bam2fq^89^ (v1.4). The sequence read, and base quality were checked using the Trimmomatic-toolkit^91^ (v0.36.5). Reads were processed using workflows managed on the Galaxy platform. Reads were trimmed by 1 base at the 3′ end, then trimmed from both ends until base calls had a minimum quality score of at least 30. Any remaining adapter sequence was removed as well. The STAR aligner^78^ (v2.4.2a) with the GRCh38^72^ reference genome and gene annotations from Ensembl release 91^73^ was used to align the trimmed reads. Gene counts were generated using HTSeq-count (v0.4.1).^92^ Quality metrics were compiled from Picard^93^ (v1.134), FASTQC^77^ (v0.11.3), Samtools (v1.2),^89^ and HTSeq-count (v0.4.1)94. Failed samples were identified as median cv gene coverage >0.8 and Aligned Counts <1 million. These samples were removed from further downstream analyses.

#### Nasal metagenomics

Taxonomic alignments were obtained from raw fastq files using the ID-seq pipeline,^135^ which first removes human sequence via subtractive alignment against human genome build 38, followed by quality and complexity filtering. Subsequently, reference-based taxonomic alignment at both the nucleotide and amino acid levels against sequences in the National Center for Biotechnology Information (NCBI) nucleotide (NT) and non-redundant (NR) databases, respectively, is carried out, followed by assembly of the reads matching each taxon. Taxa were aggregated at the genus level for analyses. Mapped taxa obtained from the ID-seq pipeline were then filtered to retain taxa with >0.1 reads per million reads sequenced (rpM) in both the NT and NR database alignments. Subsequently, a subset of previously reported common next-generation sequencing reagent contaminants^136^ (Sphingomonas, Bradyrhizobium, Ralstonia, Delftia, Propionibacterium, Methylobacterium, and Acidovorax) was filtered from the dataset. Finally, total bacterial abundance per sample was calculated by summing the rpM of all bacterial taxa present, and the Shannon Diversity Index was calculated using the vegan package v.2.6 in R.

#### Antibody titers peptide-based PhIP-seq bioinformatics analysis

For all VirScan libraries, the null distribution of each peptide’s log10 (rpK) was modeled using a set of 71 pre-pandemic healthy controls sera collected from the New York Blood Center.^137^ Peptide counts were computed for all control samples, then were converted into proportions relative to the sum of sample counts. Each sample was downsampled by 1,500,000 weighted by the proportion values. The down-sampled counts produced were then converted to rpK values. Multiple distribution fits were examined for these data, including Poisson, Negative Binomial, and Normal distributions. Quantile-quantile plots for each distribution demonstrated that the Normal distribution had the best fit across all peptides, as the median linear correlation coefficient across all peptides was the highest for this distribution. These null distributions were used to calculate p values for the observed log10(rpK) of each peptide within a given sample. For peptides with less than 10 unique values across all 71 controls, the background model was substituted with a peptide whose counts closely match the median. The calculated p values were corrected for multiple hypotheses using the Benjamini-Hochberg (BH) method. Any peptide with a corrected p value of <0.001 was considered significantly enriched over the set of pre-pandemic blood center serum controls.

To identify regions targeted by host antibodies, all library peptides were aligned to the SARS-CoV-2 reference genome, concatenating all of its open reading frames (ORFS) into a single sequence. This reference sequence was used to generate a blastp database, and all peptides in the library were aligned to it using NCBI blastp (v2.11.0). Using these data, the summation of enrichment relative to the healthy background was calculated at each position across SARS-CoV-2 for all significant peptides for each experimental sample. Finally, the results were summed at each position across all experimental samples, and the summed enrichment was plotted by position using the R ggplot2 library. Only full-length alignments (38 amino acids) were included in this analysis.

To identify clusters of peptides, CD-HIT was used to group together peptides with at least 70% AA sequence identity. Annotation categories were defined based on the composition of each cluster. Clusters containing peptides exclusive to SARS-CoV-2 or SARS-CoV1 were defined as SARS clusters. All remaining clusters were defined as Non-SARS clusters. Alphavirus strains were omitted from the downstream analysis. Only peptides significantly enriched above the control background (adj.p < 0.001) were used in this analysis. In addition, only peptides that fell into the SARS peptide clusters and aligned at full length to the SARS-Cov-2 protein reference were kept for further analysis. A sliding window algorithm, with a window size of 20 and a step size of 1, was used to sequentially sum the rpK values for every window across both the Spike and N region of the SARS-CoV-2 proteome.

#### Antibody peptide (PhIP-Seq) data pre-processing

Sequencing reads from 1,318 samples were aligned to a reference database of the full coronavirus peptide library, which consisted of 3,745 peptides, using the Bowtie2 aligner v2.2.7.^102^ Prior to the alignment, paired R1 and R2 reads from each sample were stitched together using FLASH (v1.2.11). All SAM format files outputted from Bowtie2 were converted to BAM using the samtools (v1.11)^89^ view command. The CIGAR string was examined for each aligned sequence, and all alignments where the CIGAR string did not indicate a perfect match were removed. The filtered set of aligned sequences was then translated, and only translated peptides that matched perfectly to the input library were retained for subsequent analysis. The final set of aligned peptides was tabulated to generate counts for each peptide in each individual sample. All of this analysis was automated using an R language script. Peptide counts were normalized for read depth by dividing them by the total number of peptides in the sample and multiplying by 100,000, resulting in a reads/100,000 reads (rpK) for each peptide.^102^^,^^138^^,^^139^^,^^140^

#### Serum proximity extension assay (olink) data processing

Data were analyzed using Real-time PCR analysis software via the ΔΔCt method and Normalized Protein Expression (NPX) manager. NPX is calculated in three steps from the Cq-values: (i) ΔCqsample = Cqsample − Cqextensioncontrol, (ii) ΔΔCq = ΔCqsample − ΔCqinterplatecontrol, (iii) NPX = Correction factor − ΔΔCqsample. Data were normalized using internal controls in every sample, inter-plate control (IPC) and negative controls, and correction factor and expressed as Log2 scale proportional to the protein concentration. One NPX difference equals to the doubling of the protein concentration.

Batch normalization was performed to account for potential batch effects caused by re-assayed samples which were not able to adhere to the study randomization scheme or assay condition changes including those due to assay kit lot# changes or differences in study collection phases. Olink Data Analysis Normalization employed identical reference samples in all plates. NPX value for each analyte was adjusted based on the adjust factor that makes the median of all reference samples the same for all plates. Sequential steps included: 1) the reference sample the-inter-plate-median was calculated; 2) for each assay, the pairwise difference from the inter-plate median was calculated in first step 1 for each of the reference sample on all plates; 3) plate- and assay-specific differences in step 2 were used as normalization factors; and 4) plate- and assay-specific normalization factors were added from step 3 to each value for each assay and plate.

#### Plasma global proteomics data processing and quality control

All raw timsTOF data were searched on a high-performance computing environment where Fragpipe (including MSFragger, Philosopher, and IonQuant^141^^,^^142^^,^^143^^,^^144^) was run to identify and quantify peptides and protein throughout the data.^145^ MSFragger 3.4 was run using the standard settings without the fixed modification of carboxylmethylation and with the variable modification’s oxidation and N-term acetylation. Data were scored against a human FASTA file without isoforms where SARS-COV-2 proteins were manually added. Philosopher 4.1.1 was used where PeptideProphet was used for statistical validation of identified peptides. IonQuant 1.7.17 was used for quantification, where a minimum of 1 ion was used for peptide quantification.

Genes were first filtered based on “Homo Sapiens” and “*Homo sapiens* OX = 9606”. For each sample, the “Total intensity” column was selected. Then Genes without any values across the samples were removed. Finally, sample outliers were removed. A sample is considered an outlier if its total number of quantified proteins is more than 3 standard deviations below the mean of quantified proteins of all samples. In brief, the number of proteins quantified for each sample was calculated, and log2-transformed. Then the mean and standard deviation of quantified proteins across all samples was calculated, and any samples outside 3 standard deviations were considered an outlier and removed. Finally, a protein had to be identified and quantified in at least half of all samples to be analyzed in any of the downstream analyses. We identified 508 proteins that were present in at least 699 (50%) of the samples (out of 2109 proteins in total).

#### Plasma targeted proteomics data processing

The raw data were exported into Skyline software (v20.2.1.315)^113^ for peak area and retention time refinement. The peptide intensity (average of transition pairs) and the protein abundance (average of peptide intensities) in all samples were exported from Skyline. These effects were corrected using Combat^33^ The means of the peptide intensities were used for the different protein abundances, which were exported for further analysis using RStudio Pro Server.

#### Plasma global metabolomics data processing and quality control

Raw data were measured based on LC-MS peak areas proportional to feature concentration. For quality control, missing values were imputed with half the minimum detected level for a given metabolite. Metabolites with an interquartile range of 0 were excluded from the analysis, as previously described.^146^ All features were log-transformed, normalized then Pareto-scaled to reduce variation in fold-change differences between features (Figures S5A and S5B). After pre-processing, 5 metabolites were filtered out with zero interquartile range, yielding 1012 remaining metabolites (Figure S5C). Statistical analyses for univariate, chemometrics, and clustering analysis used in-house algorithms, R statistical packages, and *MetaboAnalyst 5*.*0*.^98^^,^^147^

#### Blood CyTOF data processing and demultiplexing

Samples from a given batch were acquired on the Fluidigm Helios mass cytometer in multiple acquisitions. The resulting FCS files were normalized and concatenated using Fluidigm’s CyTOF software. The FCS file was further cleaned using the Human Immune Monitoring Center at Mt. Sinai’s internal pipeline. The pipeline removed any aberrant acquisition time windows of 3 s where the cell sampling event rate was too high or too low (2 standard deviations from the mean). EQ normalization beads that were spiked into every acquisition and used for normalization were removed, along with events that had low DNA signal intensity.

The pipeline was also used to demultiplex the cleaned and pooled FCS files into single sample files. The cosine similarity of every cell’s Pd barcoding channels to every possible barcode used in a batch was calculated and then was assigned to its highest similarity barcode. Once the cell had been assigned to a sample barcode, the difference between its highest and second highest similarity scores was calculated and used as a signal-to-noise metric. Any cells with low signal-to-noise were flagged as multiplets and removed from that sample. Finally, acquisition multiplets were removed based on the Gaussian parameters Residual and Offset acquired by the Helios mass cytometer.

Cells from a single biological sample were clustered into 1000 K-means clusters. A subset of samples was then selected and manually annotated into cell types using Clustergrammer2’s widget interface (https://github.com/ismms-himc/clustergrammer2) to create a training dataset (n x n matrix of cell types by median marker intensities) for each manually annotated sample.

To annotate a given sample’s 1000 K-means clusters, the cosine similarity of every cluster to all possible cell types within the training datasets was calculated, and that cluster was assigned to either its highest similarity score cell type or the greatest consensus cell type across the training datasets. Finally, the cluster cell-type annotation was assigned back to the single cells within that cluster, and the number of cells was calculated for a cell type within a given single sample.

#### Blood CyTOF count normalization

To account for differences in cell events acquired for each sample, the cell population count matrix was converted into cell frequencies. We first processed immune cell frequencies of the more broadly defined cell subsets (e.g., CD4 T cells, B cells, monocytes, etc.) as a percentage of total CD45^+^ immune cells by excluding debris, RBCs, platelets, and multiplets. Next, we processed the broadly defined cell subsets as a percentage of all non-granulocytes by further excluding eosinophils, neutrophils, and basophils. We also processed immune cell frequencies of the more granularly defined subsets (e.g., CD4 effector memory T cells, Naive B cells, CD14^+^CD16^−^classical monocytes, etc.) as a percentage of total CD45^+^ immune cells and as a percentage of non-granulocytes.

#### PBMC transcriptomics data processing and quality control

Processing and quality control was performed using an internal Snakemake workflow for RNA-Seq analysis (Github: https://github.com/yerkes-gencore/IMPACC-RNA_Seq). Reads were trimmed for adapter sequence and quality score with cutadapt v1.14112. Reads were aligned with STAR v2.4.2a91 to a composite reference of human (GRCh38)^72^ reference sequence with gene annotations from Ensembl release (release 91)^73^ and SARS-CoV-2 (NCBI strain MN908947.3).^74^ Transcript abundance estimates were calculated internal to the STAR aligner91 using the algorithm of htseq-count94. Sequencing quality metrics were determined using FastQC^77^ (v0.11.5), alignment quality metrics with Picard tools (v2.22)^93^ and STAR91logs and gene counts, including average quality per read > Q30, percent and absolute counts of reads uniquely mapped to annotated transcripts.

#### Genetics data processing and quality control

Genotype data were processed as previously described.^148^ Briefly, it was required that samples had genotyping rate >95%; and markers had minor allele frequency >1% and were within Hardy-Weinberg equilibrium (p < 1e-6). After removing 8 sample outliers in a heterozygosity/missingness space, 483 samples remained.

Chromosomal sex was then inferred from rates of X chromosome homozygosity (XHE), using 26,051 X chromosome markers. As expected, two clear clusters of individuals were found, corresponding to males (one X chromosome, high XHE) and females (two X chromosomes, low XHE). 5 samples where genetically determined sex was discordant with physician-reported sex at birth in the clinical database were removed from the subsequent genetics analysis.

Duplicate samples were also removed to prevent bias in downstream genetic association testing. Using all autosomal markers, the proportion of identity by descent (IBD, the sharing of DNA segments from common ancestors) was calculated between all pairs of individuals. A value near 1 denotes that the entire genome is identical and inherited from the same common ancestor: the pair of samples are therefore either from the same person or from monozygotic twins. 12 samples with an IBD greater than 0.98, denoting duplication, were removed leaving 466 participants for genetic association testing.

#### Data quality assurance

Data quality assurance (QA) refers to the curation of raw datasets from their generation by an assay core to the production of their canonical forms as the bases for analyses within the IMPACC network. A typical assay core-generated raw dataset is comprised of two components: [1] a “Counts” matrix of samples (as rows) and assay features (as columns) values, and [2] a “Metadata” matrix of the sample identifiers and assay-core specific processing and quality control features for each sample in the “Counts” matrix. Each IMPACC sample has a unique global sample identifier (sample_id) associated with the subject and biosample extraction time in the study. Assay feature values may be continuous real numbers, categorical variables, or character strings of nucleotides, depending on the assay type. Assay core-specific processing features may include technical quality control metrics of the sample assay. First, sample identifiers are checked for concordance between “Counts”, “Metadata” and the central IMPACC database of sample identifiers, clinical and assay core processing parameters. Second, depending on the assay type, the sample-wise distributions of Counts values are qualitatively investigated for technical anomalies. For the genetics association testing, the sex/kinship of a participant derived from their sample Counts variables are compared with their clinical records. Lastly, for downstream analyses, canonical and cumulative “Counts” and “Metadata” matrices with the well-defined and standardized assay, technical, and additional QA feature annotations are generated for each assay type.

#### Data preparation and batch effect evaluation

Samples included for analysis have undergone prior core internal and assay-specific quality control steps. In addition, proper procedures for quality assurance outlined previously were performed to ensure the data standards for each assay were met. Table S13 provides information on additional steps adopted to prepare the data for statistical analysis.

For each assay, we first filtered out samples using the sample filtering criterion and followed by a filtration on features based on the feature filtering criteria, we performed data imputation and data transformation as indicated in the table. N/A: no additional step taken. Half-min: replacing missing value using half of the minimum of observed values for the corresponding feature. Impute.knn: using impute.knn function from R package impute. Pareto-Scaling: in-house function of dividing each variable by the square root of the standard deviation. We evaluated the influence of potential batch effects on different assays using Principal variance component analysis (PVCA) or PCA.

#### Common statistical analyses framework

##### Overview of common analyses framework

Data from each assay were pre-processed to a counts matrix as described in the section “OMIC-specific preprocessing from raw to computable matrices”. Then for each of the omics assays, we used Principal Variance Component Analysis (PVCA) to identify co-variates to include in the models. These included participant enrollment site, elapsed time from self-reported disease onset, sex, age, ethnicity, race, and core lab site. Covariates that explained >10% of the variance were either used for batch correction of the data (RNA-seq) or as covariates in the models as detailed shortly after in the section “data preparation and batch effect evaluation*”*. To reduce the dimensionality of assay readouts with >50 features for analysis, we utilized all available samples to identify feature correlated modules (referred to here as “modules”) for each assay using WGCNA.^13^ The number of modules ranged from six modules in the Olink assay to 41 modules in the global plasma metabolomics assay. Details of tuning parameters used in WGCNA are given in the section “weighted gene correlation network analysis (WGCNA)”. For a given module in an assay, we define the module values across samples as the first principal component constructed using features included in this module. To aid in interpretation, the features in each module were annotated to biological processes by performing an enrichment analysis leveraging biological knowledge bases, such as MSigDB Hallmark.^39^ Olink WGCNA modules were annotated by testing the overlap between the soluble proteins in a module with the cytokine annotations in ImmuneXpresso^25^ and the COVID-19 Drug and Gene Set Library^75^ using Fisher’s exact test.

We investigated the behavior of each feature (or module) both at visit 1 (within 72 h of hospital admission) and longitudinally for scheduled visits (visits 1–6, up to 28 days post-hospital admission) and correlated it with clinical outcomes. We first tested whether the value of each feature at visit 1 exhibited a monotonic trend from mildest (TG1) to most severe (TG5) disease course using mixed effect ordinal regression (clmm). Features with false discovery rate (FDR) < 5% were considered significant based on adjusted p value (referred to as adj.p) (Chen et al., 2021). To identify longitudinal associations, we next tested if feature kinetics across the whole time-course (visits 1–6) were different across the clinical trajectory groups via a generalized additive model with mixed effects (gamm4). Features for which the average (referred to as intercept in the gamm4 documentation) or shape (referred to as the smoothing term in the gamm4 documentation) differed between the clinical trajectory groups at FDR<5% were considered significant. For both the visit 1 and longitudinal analyses, significant features were further tested for differences between each pair of TGs to facilitate interpretation. More details on the association tests are given in the section “visit 1 and longitudinal model analysis”.

#### Weighted gene correlation network analysis (WGCNA)

We used R package Weighted Gene Coexpression Network Analysis (WGCNA) (v1.71)^13^ to identify modules of correlated features from high throughput assays, specifically, RNA-seq, proteomics, metabolomics, Olink. We used the data as input to WGCNA after following assay specific QC/QA steps and additional data preparation steps as described in Table S14. For each assay, we first computed optimum soft-thresholding power parameter using `*pickSoftThreshold`* function. Then, we built modules using `*blockwiseModules*` function with the selected power parameter. When building the modules, we set networkType = ”signed”, TOMType = ”unsigned”, maxPOutlier = 0.1. Details of the other assay-specific parameters are provided below. Note that escalation samples were included in the WGCNA module creation, while only samples from scheduled visits were part of the visit 1 and longitudinal modeling.^10^

#### Visit 1 and Longitudinal Model Analysis

We performed a mixed effect analysis using module levels from baseline visit samples and investigated (1) if there is an ordinal trend from trajectory group 1 to trajectory group 5 and (2) if any pair of groups exhibit significant differences. This analysis included enrollment sites as a random effect, and age group, and sex as fixed effects, and tested for the ordinal trend with the R package clmm and pairwise difference with the R package lmer. We identified significant WGCNA modules whose adjusted p values are below 0.05. Significant modules can potentially be used for separating clinical groups at hospital admission.

We next moved to longitudinal analysis for scheduled visits (visits 1–6) to identify WGCNA modules whose trajectories differ for different clinical groups. We performed a mixed generalized additive modeling analysis and modeled the module levels as a smooth function of admission time using the R package gamm4. For each pair of groups, we tested if the two groups have different longitudinal trends for the WGCNA modules after including the participant ID and enrollment site as random effects along with sex and age group as fixed effects. We claimed significance when the adjusted p value is below 0.05, and significant modules could indicate interesting molecular dynamics across clinical groups. Physician-reported sex at birth was used in all analyses except genetic association testing.

#### Identification of overlap between assays’ annotations

For each assay, geneset set analysis was used to identify pathways overrepresented among feature (WGCNA modules or individual features) associated with the trajectory groups. To identify pathways enriched in two assays or more, the name/label of the pathways were matched across assays based on the similarity of the name using fuzzy matching (match allowing for mismatches characters) as implemented in the function stringdist_join of the R package fuzzyjoin (with default parameters). Incorrect matches were filtered out manually.

### Additional resources

Clinical trials number: NCT04378777.

Link: https://clinicaltrials.gov/ct2/show/NCT04378777?term = IMPACC&draw = 2&rank = 1.

## Data Availability

Data files are available at ImmPort under accession number SDY1760 and dbGAP accession number phs002686.v1.p1. Accession numbers are listed in the key resources table. Additional supplementary items are available from Mendeley Data at https://doi.org/10.17632/vcskpv8tjk.1. All analysis codes have been deposited at Bitbucket: https://bitbucket.org/kleinstein/impacc-public-code^120^ and are publicly available as of the date of publication. DOIs are listed in the key resources table. Any additional information required to reanalyze the data reported in this paper is available from the lead contact upon request.

## Supplemental information

Document S1. Figures S1–S13

Table S1. Demographics and clinical characteristics of cohort participants at baseline (N = 540), related to Figure 1

Table S2. Regions of seroreactivity within the SARS-CoV-2 proteome (antibody titers: peptides) modules and association, related to Figure 2

Table S3. Sex, age, and trajectory group distribution of participants with and without auto-Abs against IFNs, related to Figure 2

Table S4. Olink modules and association with clinical outcome, related to Figure 3

Table S5. Targeted proteomics modules and association with clinical outcome, related to Figure 3

Table S6. Global proteomics modules and association with clinical outcome, related to Figure 3

Table S7. Global metabolomics modules and association with clinical outcome, related to Figure 4

Table S8. Blood CyTOF modules and association with clinical outcome, related to Figure 5

Table S9. PBMC transcriptomics modules and association with clinical outcome, related to Figure 6

Table S10. Genomic associations with COVID-19 hospitalization previously reported by HGI, related to Figure S8

Table S11. Nasal transcriptomics sample module results, related to Figure 6

Table S12. Nasal metagenomics results and association results, related to Figure S10

Table S13. Information on data preparation, related to STAR Methods

Table S14. Number of samples used for WGCNA module creation, along with assay-specific parameters, related to STAR Methods

Document S2. Supplemental author information

Document S3. Article plus supplemental information
