# Supplementary material for: Multi-omic longitudinal study reveals immune correlates of clinical course among hospitalized COVID-19 patients
Source: Cell Rep Med. 2023 May 23;4(6):101079. doi: 10.1016/j.xcrm.2023.101079 (PMC10203880; doi:10.1016/j.xcrm.2023.101079)
Supplement: Document S1. Figures S1–S13 [file mmc1.pdf]

Cell Reports Medicine, Volume *4*

Supplemental information

Multi-omic longitudinal study reveals immune correlates of clinical course among hospitalized COVID-19 patients

Joann Diray-Arce, Slim Fourati, Naresh Doni Jayavelu, Ravi Patel, Cole Maguire, Ana C. Chang, Ravi Dandekar, Jingjing Qi, Brian H. Lee, Patrick van Zalm, Andrew Schroeder, Ernie Chen, Anna Konstorum, Anderson Brito, Jeremy P. Gygi, Alvin Kho, Jing Chen, Shrikant Pawar, Ana Silvia Gonzalez-Reiche, Annmarie Hoch, Carly E. Milliren, James A. Overton, Kerstin Westendorf, IMPACC Network, Charles B. Cairns, Nadine Rouphael, Steven E. Bosinger, Seunghee Kim-Schulze, Florian Krammer, Lindsey Rosen, Nathan D. Grubaugh, Harm van Bakel, Michael Wilson, Jayant Rajan, Hanno Steen, Walter Eckalbar, Chris Cotsapas, Charles R. Langelier, Ofer Levy, Matthew C. Altman, Holden Maecker, Ruth R. Montgomery, Elias K. Haddad, Rafick P. Sekaly, Denise Esserman, Al Ozonoff, Patrice M. Becker, Alison D. Augustine, Leying Guan, Bjoern Peters, and Steven H. Kleinstein

Multi-omic longitudinal study reveals immune correlates of clinical course among hospitalized COVID-19 patients

Supplementary Figures

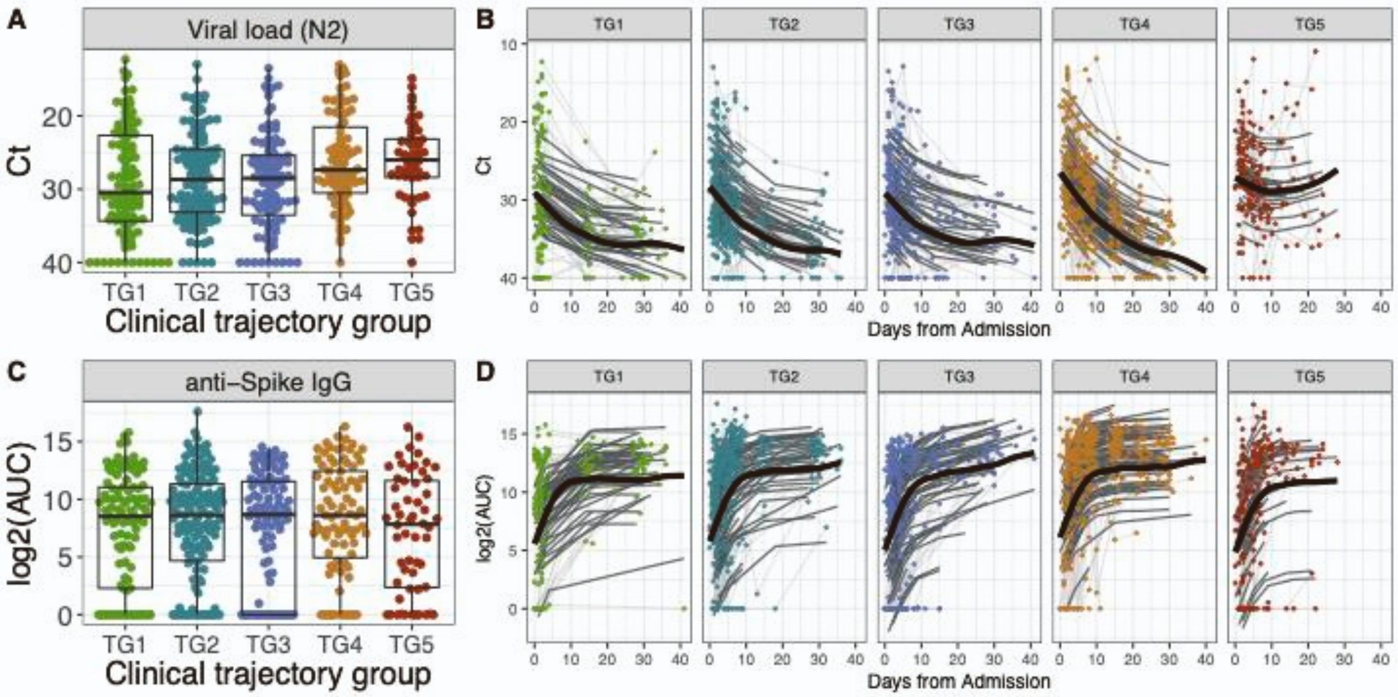

Figure S1: Viral load and antibody responses in hospitalized COVID-19 participants. Related to Figure 2. (A) Viral loads (SARS-CoV-2 N2 gene Ct values) measured from samples collected at hospital admission are significantly higher in more disease severe groups (adj. p = 0.037). Shown are median values (horizontal lines), interquartile ranges (boxes), and 1.5 IQR (whiskers), as well as all individual points. (B) Viral loads (SARS-CoV-2 N2 gene Ct values) from samples collected during the first 28 days of hospital admission differ significantly among trajectory groups (shape: adj.p = 0.0003, average: adj.p = 1.68e-05). (C) Anti-Spike IgG AUC values measured from samples collected at hospital admission are lower in the more disease severe group, TG5 (adj. p = 0.68). Shown are median values (horizontal lines), interquartile ranges (boxes), and 1.5 IQR (whiskers), as well as all individual points. (D) Anti-Spike IgG AUC values from samples collected during the first 28 days of hospital admission differ significantly among trajectory groups (shape: adj.p = 0.013, average: adj.p = 0.07).

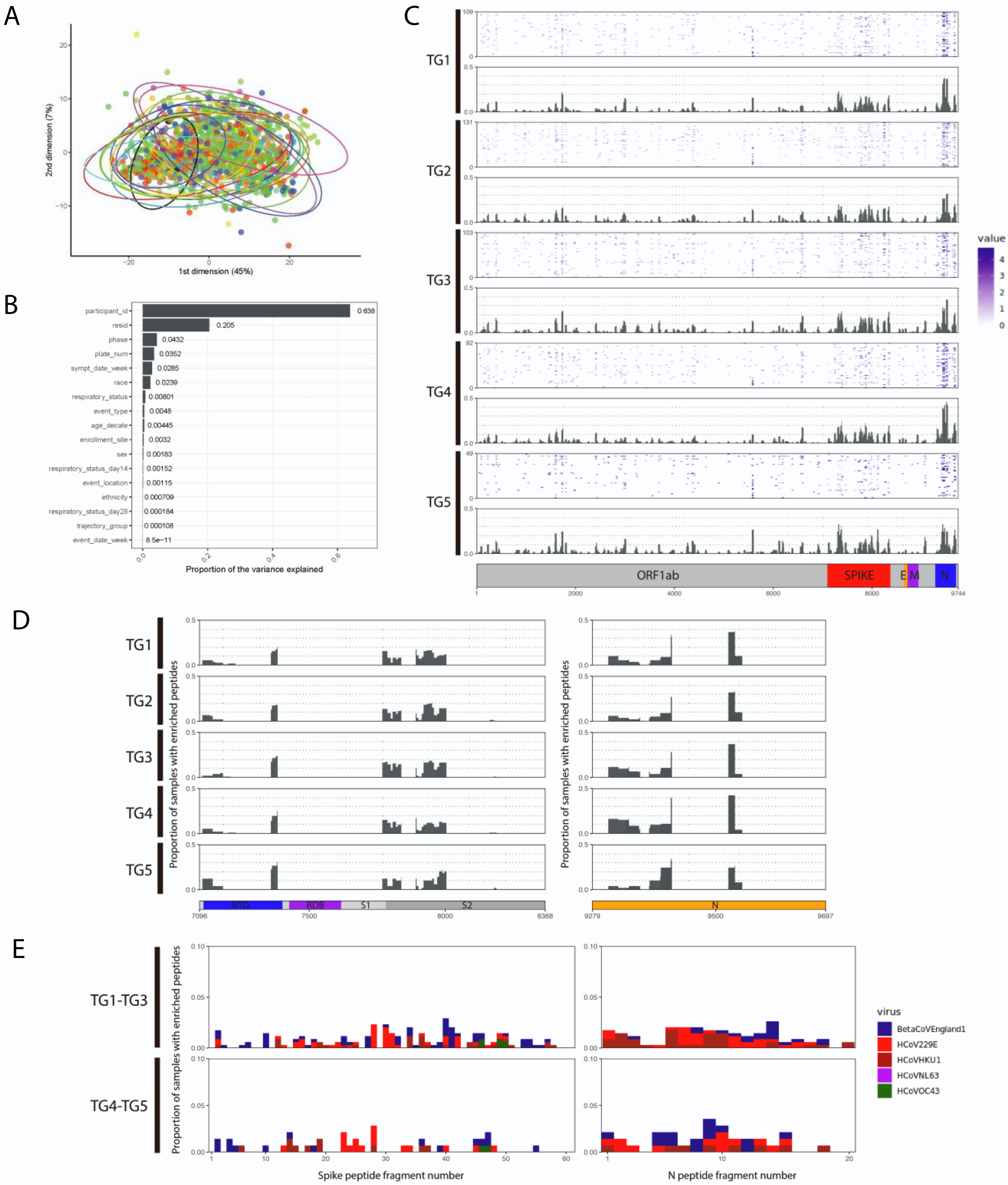

Figure S2. Sero-reactivity to coronavirus peptides in hospitalized COVID-19 patients. Related to Figure 2. (A) Principal component analysis among all enrollment sites, which included all serum phage immunoprecipitation sequencing (PhIP-Seq) SARS-CoV-2- positive samples and healthy controls (black). (B) Principal variance component analysis of all serum PhIP-seq samples in which each row of the bar plot depicts the proportion of variance contributed by site-specific, experiment-specific, and patient-specific demographic and clinical variables (C) Proportionate seroreactivity (bar plots) and summed log10 RPK (heatmaps) expression of all COVID19 positive samples across the SARS-CoV-2 proteome for all five trajectory groups (TGs) at hospital admission. For each sample, summed RPK and proportionate reactivity values were calculated by only including peptides determined to be enriched above pre-pandemic healthy controls. Each TG group was processed separately. (D) Longitudinal, proportionate SARS-CoV-2 spike protein and nucleoprotein seroreactivity of all SARS-CoV-2-positive samples using a 20 amino acid sliding window. Proportionate reactivity was calculated separately for each TG, revealing eight antigenic regions (E). Proportionate spike protein and nucleoprotein seroreactivity of other seasonal human CoVs at hospital admission with mild to moderate COVID-19 (TG1-TG3) relative to severe COVID-19 (TG4-TG5) and vice versa.

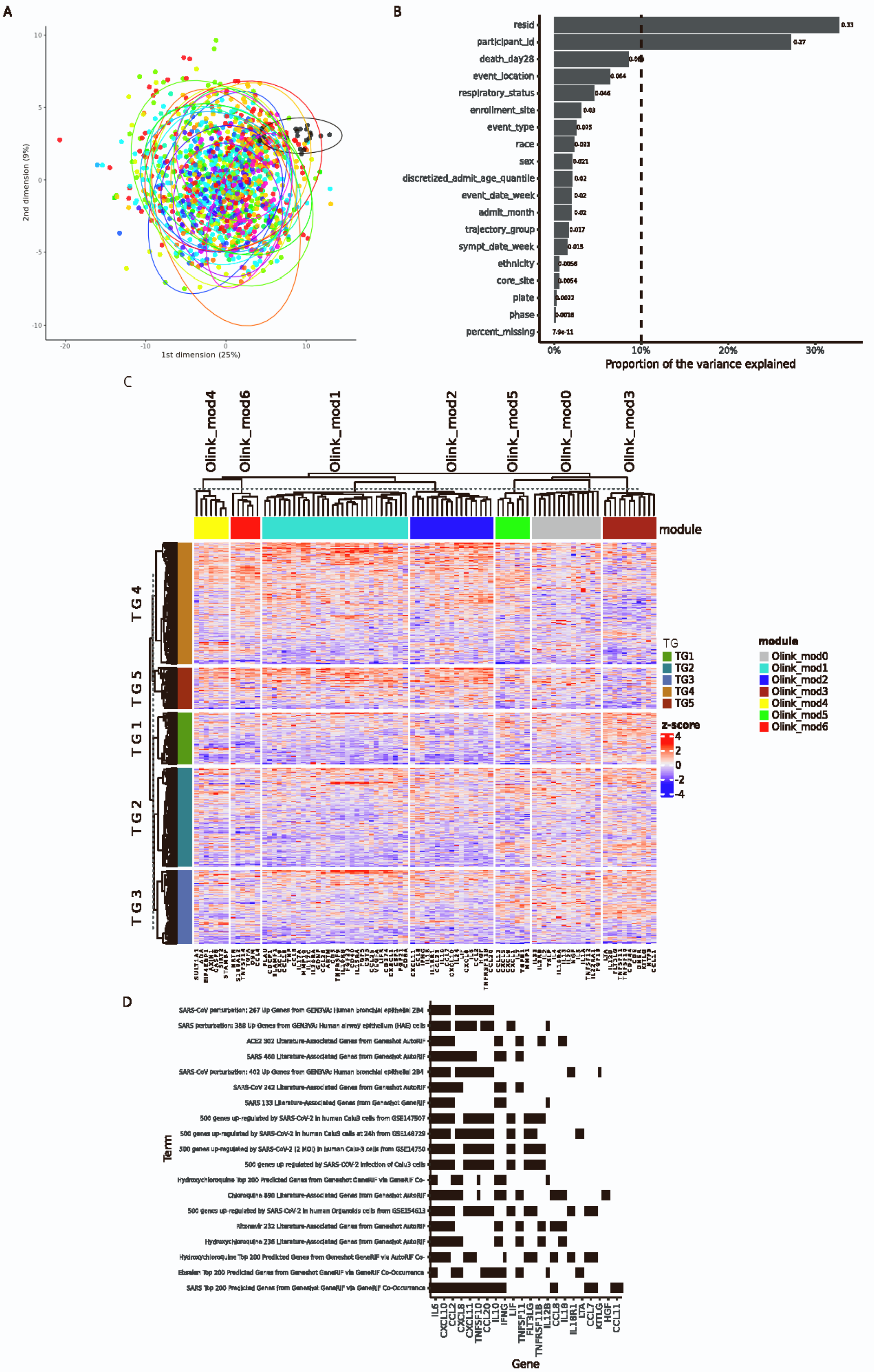

Figure S3: Cytokines in the pro-inflammatory modules are directly induced by SARS-CoV-2 infection. Related to Figure 3. (A) Principal component analysis including samples from COVID-19 participants and healthy controls (black). Co-centric ellipses encompass 95% of the samples from each enrollment site. (B) Principal variance component analysis using Olink samples from confirmed COVID-19 cases. Bar plot depicts the proportion of variance in the Olink data explained by each technical and clinical variable. (C) Heatmap presenting the six modules identified by WGCNA analysis. Hierarchical clustering (Euclidean distance and complete linkage) was used to regroup samples (rows) and Olink features (columns) with similar expression levels across the samples. (D) Soluble proteins in the pro-inflammatory module (Olink.mod2) were tested for overlap with the COVID-19 Drug and Gene Set Library using a Fisher's exact test. This analysis revealed an enrichment of protein coding genes known to be induced by SARS-CoV-2 infection in that module.

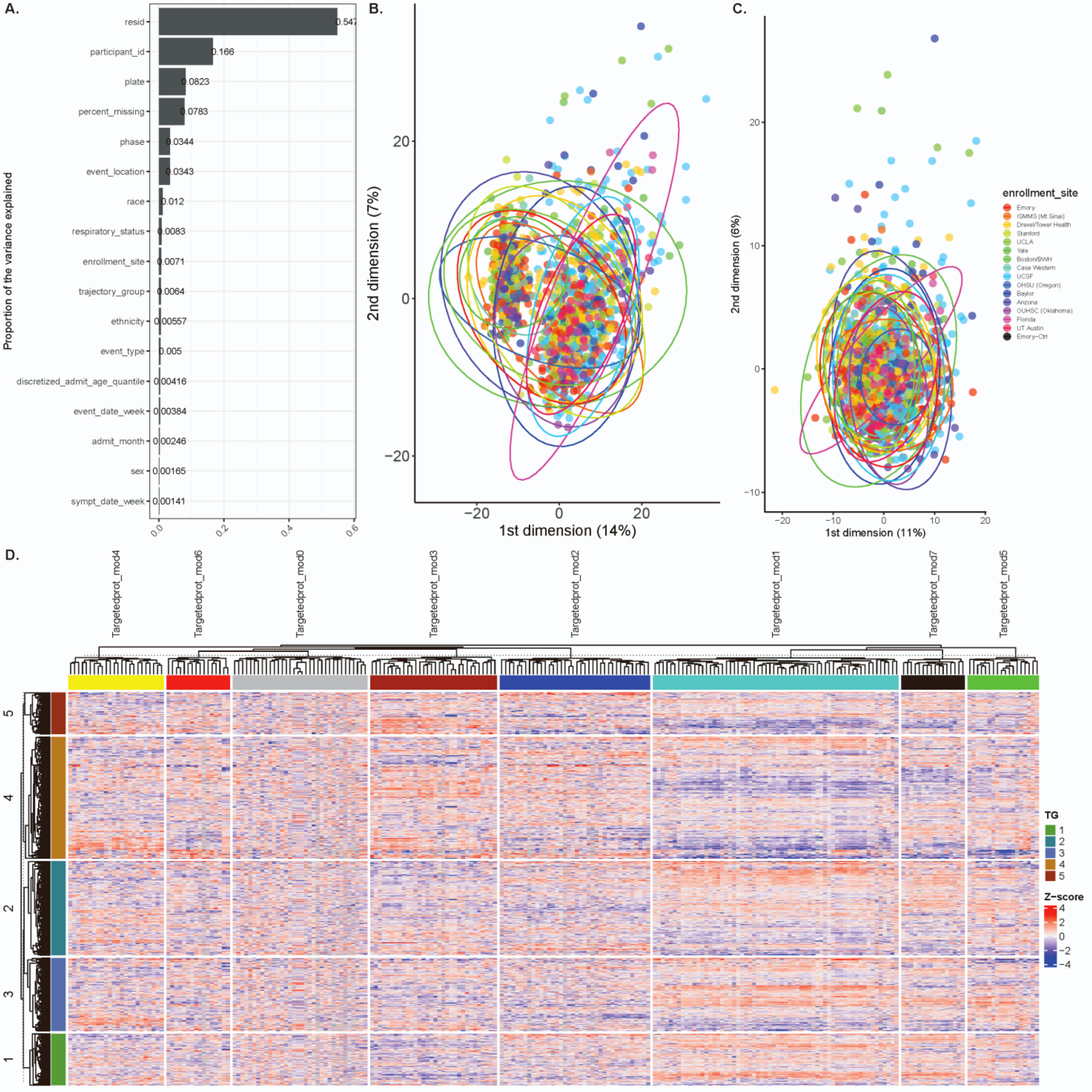

Figure S4. Targeted Proteomics. Related to Figure 3. Targeted: (A). Principal variance component analysis of all plasma targeted proteomics in which each row of the bar plot depicts the proportion of variance contributed by site-specific, experiment-specific, and patient-specific demographic and clinical variables. (B). Principal component analysis among all enrollment sites included all plasma targeted proteomics samples (B) before batch correction and (C) after batch correction.(D). Heatmap presenting the eight modules identified by WGCNA analysis. Hierarchical clustering (Euclidean distance and complete linkage) was used to regroup samples (rows) and targeted proteomics features (columns) with similar expression levels across the samples.

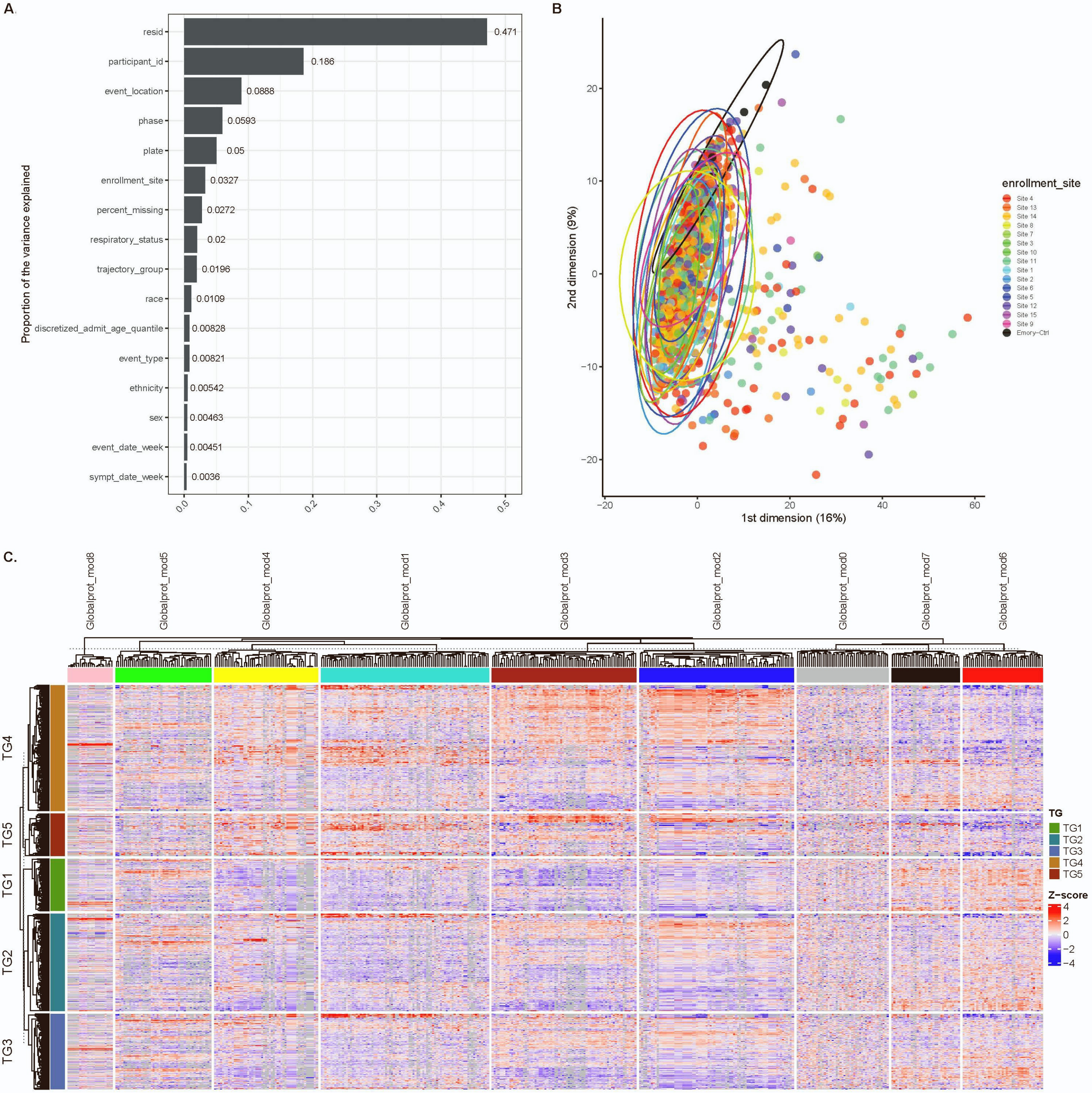

Figure S5. Global Proteomics. Related to Figure 3. (A) Principal variance component analysis of all global proteomics samples in which each row of the bar plot depicts the proportion of variance contributed by site-specific, experiment-specific, and patient-specific demographic and clinical variables (B). Principal component analysis among all enrollment sites included all global proteomics samples and healthy controls (black). (C) Heatmap presenting the eight modules identified by WGCNA analysis. Hierarchical clustering (Euclidean distance and complete linkage) was used to regroup samples (rows) and global proteomics features (columns) with similar expressions across the samples.

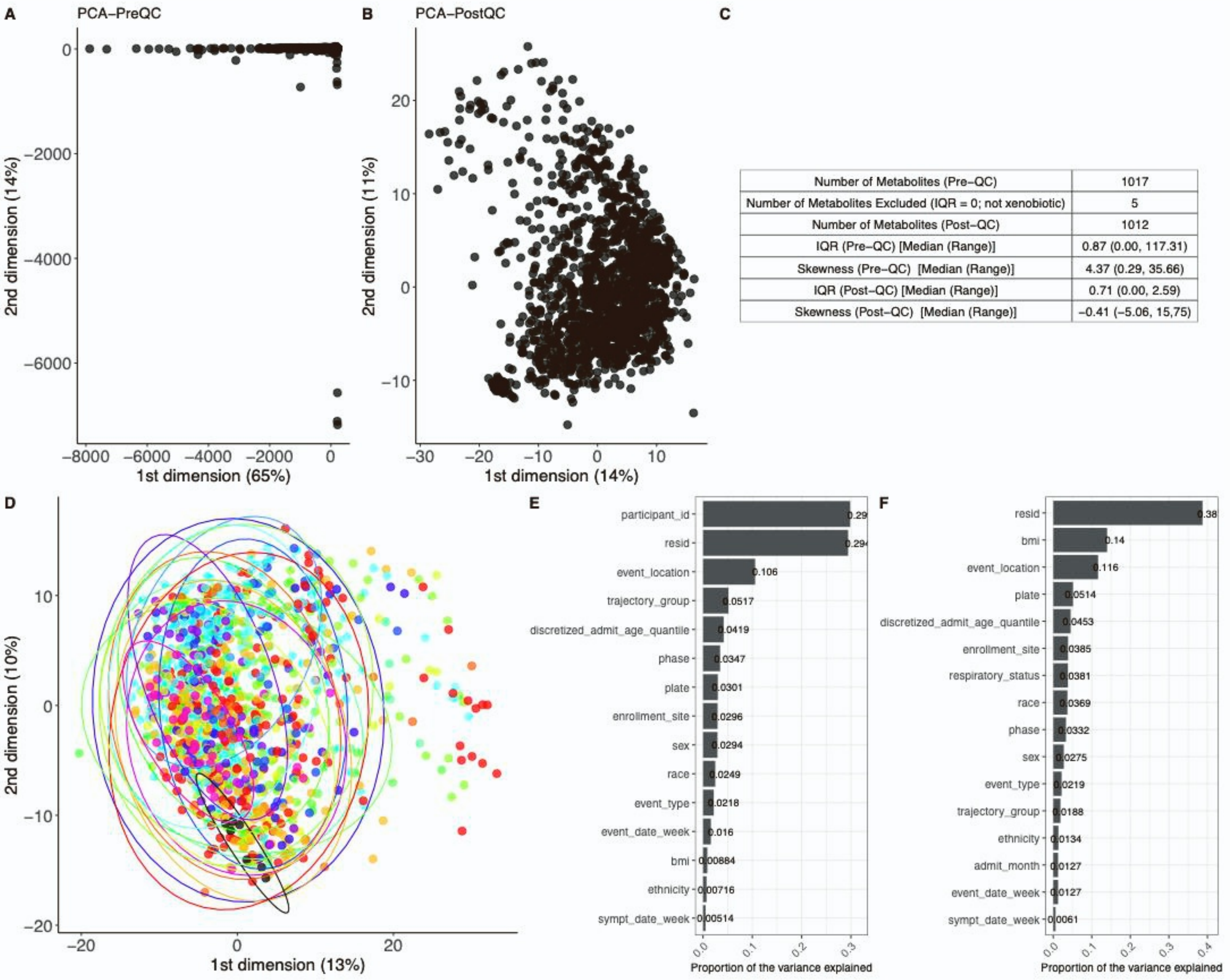

Number of Metabolites (Pre–QC)	1017
Number of Metabolites Excluded (IQR = 0; not xenobiotic)	5
Number of Metabolites (Post–QC)	1012
IQR (Pre–QC) [Median (Range)]	0.87 (0.00, 117.31)
Skewness (Pre–QC) [Median (Range)]	4.37 (0.29, 35.66)
IQR (Post–QC) [Median (Range)]	0.71 (0.00, 2.59)
Skewness (Post–QC) [Median (Range)]	–0.41 (–5.06, 15,75)

Figure S6. QC, PCA, and PVCA results for Global Metabolomics. Related to Figure 4. (A) PCA scores plot (PC1 v. PC2) of Pre-QC metabolomics data. (B) PCA scores plot (PC1 vs. PC2) of Post-QC metabolomics data. (C) Quality Control Metrics Summary for Plasma Global Metabolomics. (D) PCA of metabolomics samples, colored for sample collection sites. (E) PVCA plot for longitudinal analysis. (F) PVCA plot for visit 1 analysis.

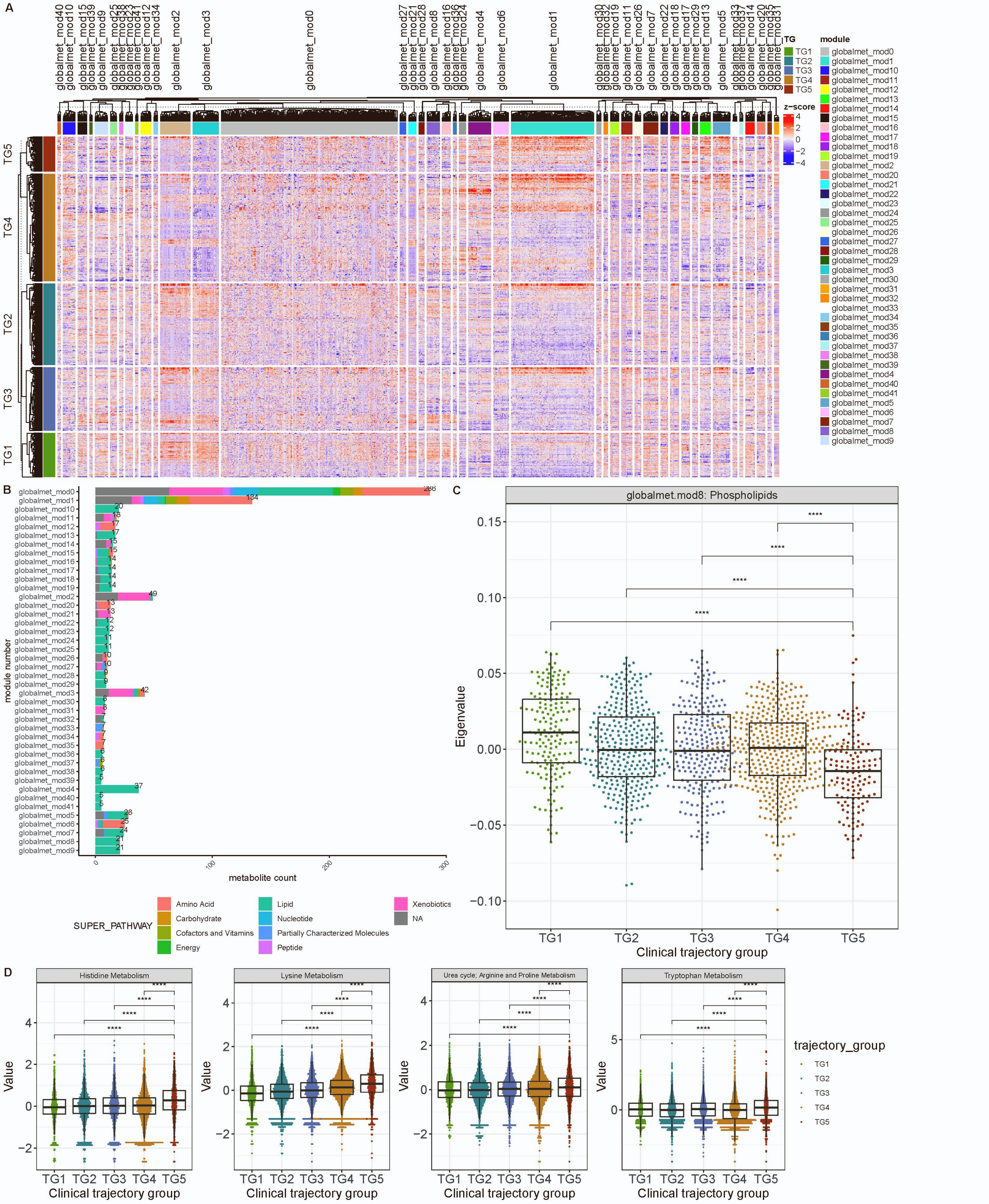

Figure S7. QC, PCA, and PVCA results for Global Metabolomics. Related to Figure 4. (A) Module grouping using Weighted Gene Correlation Network Analysis (WGCNA) clusters metabolites into 42 modules. Heatmap shows module cluster assignment of metabolites with the column as metabolite feature and row as each individual patient. Red indicates increased level, blue decreased level. (B) Annotations of the metabolites in each module assignment are structurally grouped corresponding to their super pathway classification (e.g., amino acid, carbohydrate, cofactors and vitamins, energy, lipid, nucleotide, partially characterized molecules, peptide, xenobiotics). NA depicts unnamed metabolites that have not yet been fully annotated to their corresponding super pathway. (C). Metabolites under phospholipid pathways were selected and compared across trajectory groups. (D). Metabolites individually annotated as part of Histidine, Lysine, Urea cycle: Arginine and Proline Metabolism, and Tryptophan Metabolism demonstrate significance across trajectory group as depicted by boxplots.

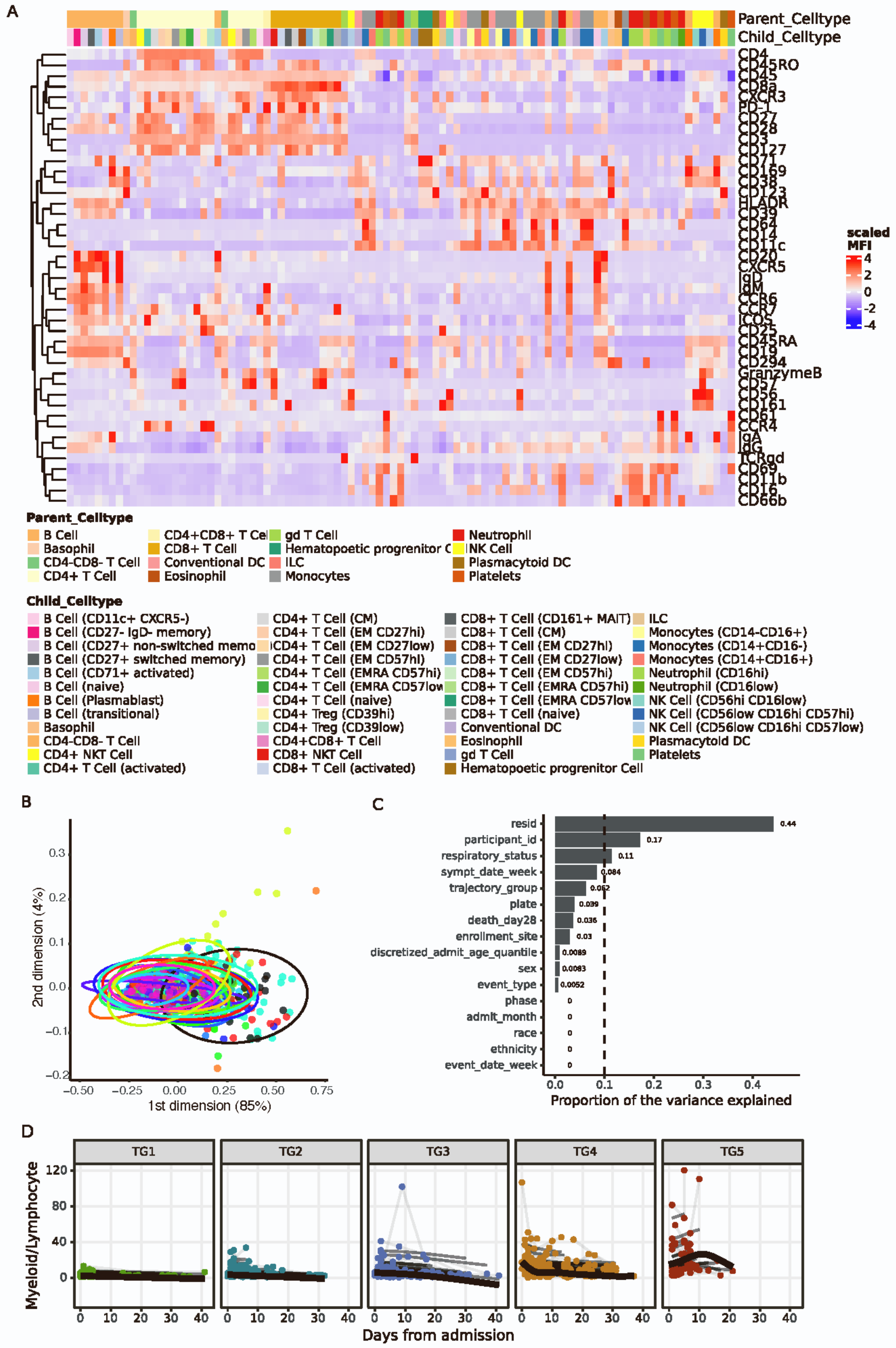

Figure S8. Blood CyTOF. Related to Figure 5. (A) Heatmap of single sample annotation showing mean marker expression across 1000 K-means clusters. (B) PCA on parental cell frequencies normalized for the total number of events per samples (C) PVCA on parental cell frequencies normalized for the total number of events per sample. (D) Longitudinal analysis of the myeloid (granulocytes + monocytes) to lymphocyte ratio.

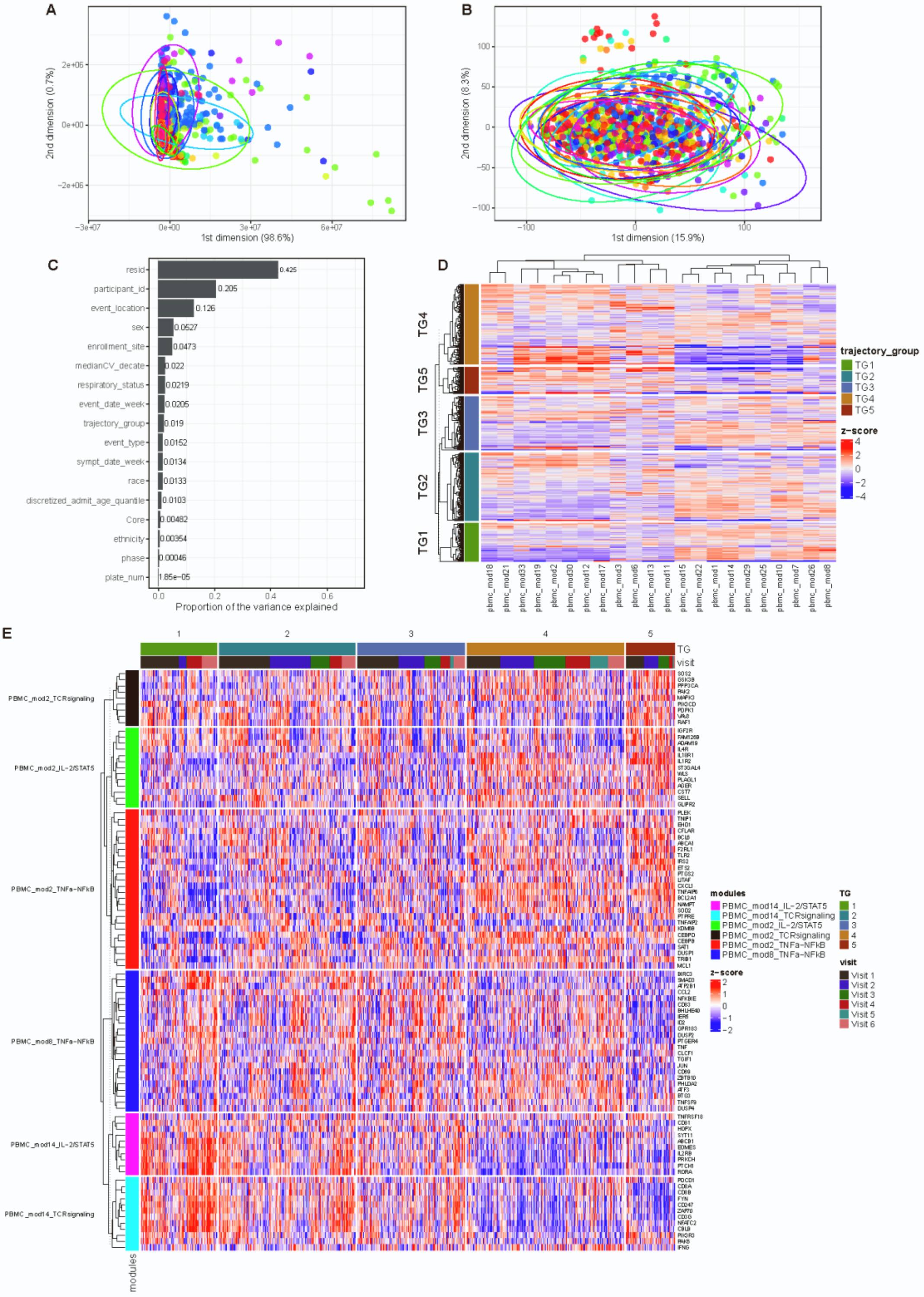

Figure S9. PBMC Transcriptomics. Related to Figure 6. (A) Principal component analysis before batch correction using PBMC RNA-seq data from samples across all enrollment sites. (B) Principal component analysis after batch correction. The shape of the point denotes the sample sequencing core site. (C) Principal variance component analysis. Bar plots depict the proportion of variance in the PBMC transcriptomic data explained by each technical and clinical variable. (D) Heatmap presenting the 22 gene modules statistically associated with clinical trajectory group longitudinally by both shape and average $p < .05$. Hierarchical clustering grouped these modules by similarity (columns). The rows are ordered by the clinical trajectory group. The scale represents the log normalized values of the WGCNA module expression. (E) A heatmap showing unique genes in common pathways between PBMC.mod2, PBMC.mod14, and PBMC.mod8. Rows represent genes ordered in an unsupervised manner clustered within module and pathway groups. Columns represent samples grouped by clinical trajectory group ordered by participant visit number from left to right within each trajectory group. Genes in the PBMC.mod2 pathways generally have increased expression across the trajectory group, while genes in PBMC.mod8 and PBMC.mod14 decrease across the trajectory group. Each gene is unique to a module.

A

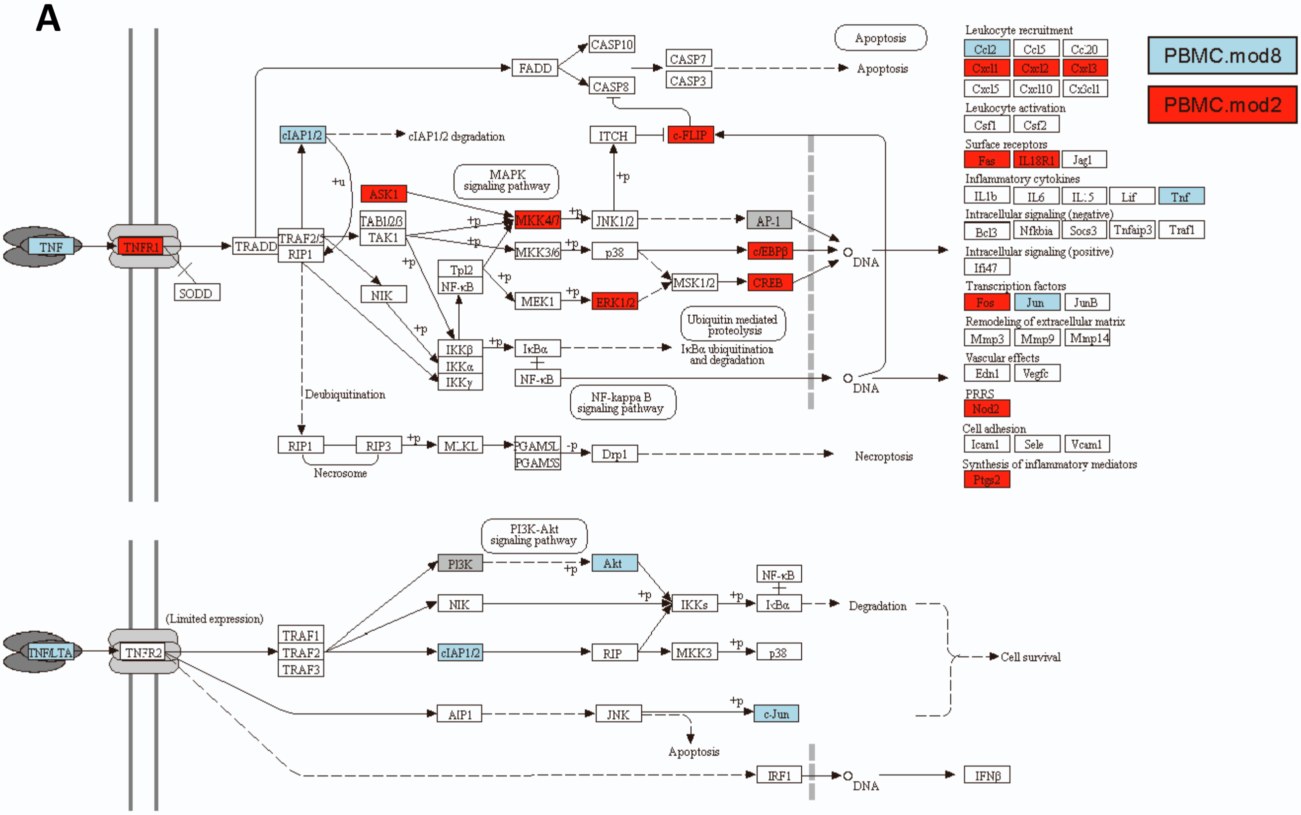

Figure S10. PBMC Transcriptomics. Related to Figure 6. (A) KEGG graph of the TNF signaling pathway (hsa04668) colored by gene module assignment. PBMC.mod8 (decreased expression in TG5) is in light blue and PBMC.mod2 (increased expression in TG5) is in red.

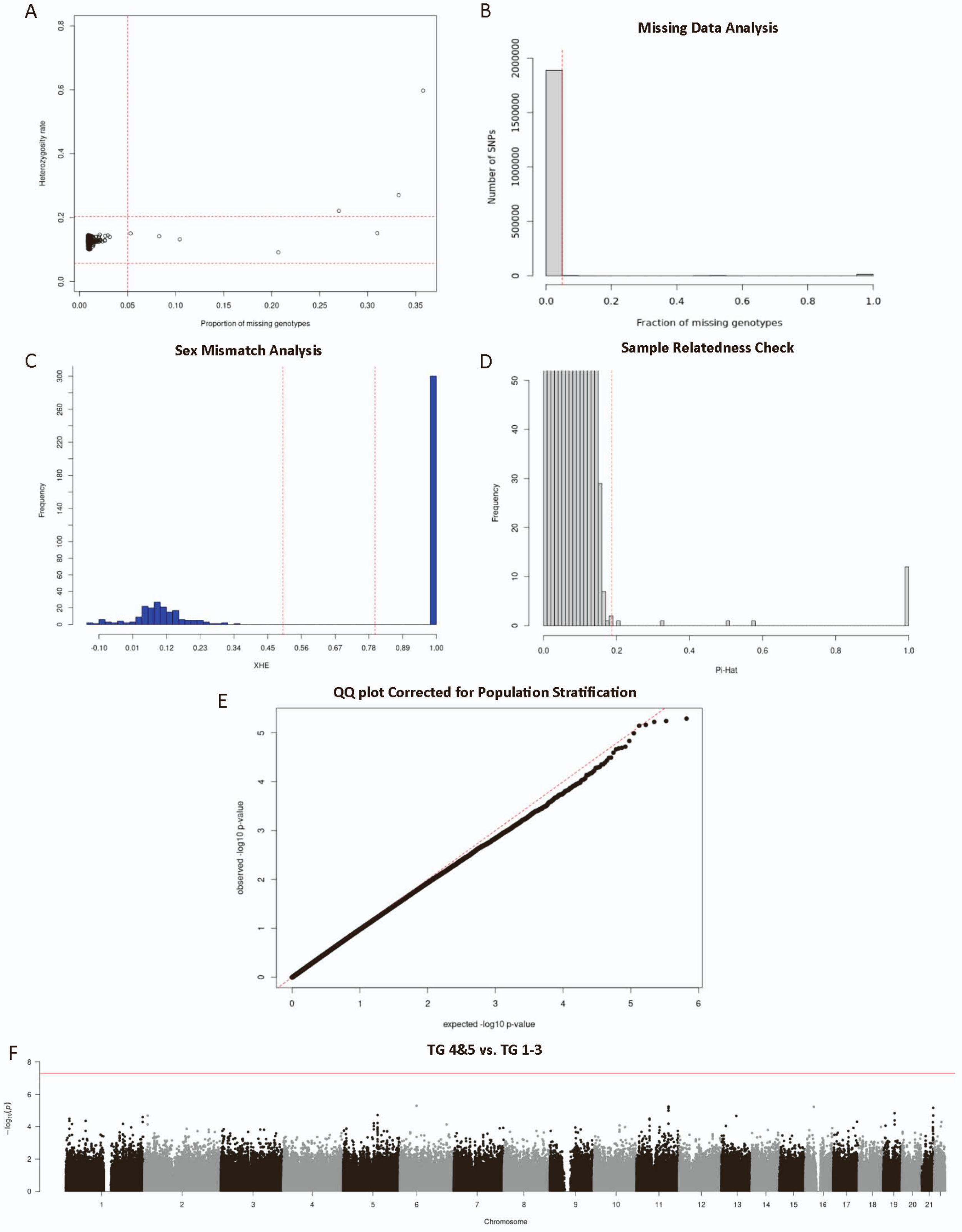

Figure S11: Genetics. Related to Table S10. (A) Analysis of heterozygosity rate and missing genotype was carried out on 491 samples. 8 samples with heterozygosity greater or less than 3 SD from the mean or with greater than 5% missing genotype data were removed. (B) 24,581 SNPs with greater than 5% missing data across all samples were removed. (C) Genotype determined sex based on X-chromosome homozygosity (XHE). Samples with less than 0.5 XHE were classified as female, while samples greater than 0.8 XHE were classified as male. 5 samples where XHE was less than 0.5 but physician-reported sex at birth was male were excluded from the genetic association analysis. (D) Sample relatedness based on identity by descent (IBD). 12 samples with greater than 0.98 pi-hat were removed. This left 466 samples that passed all the genetics QC steps, corresponding to 466 IMPACC study participants. (E) QQ plot for genotype data after accounting for population substruction. The red line indicates the uniform distribution of association p-values. (F) Manhattan plot of genome-wide association study for severe TG4&5 vs. milder TG1-3 disease trajectories (n=139 vs.327 cases). Red line indicates the threshold for novel associations.

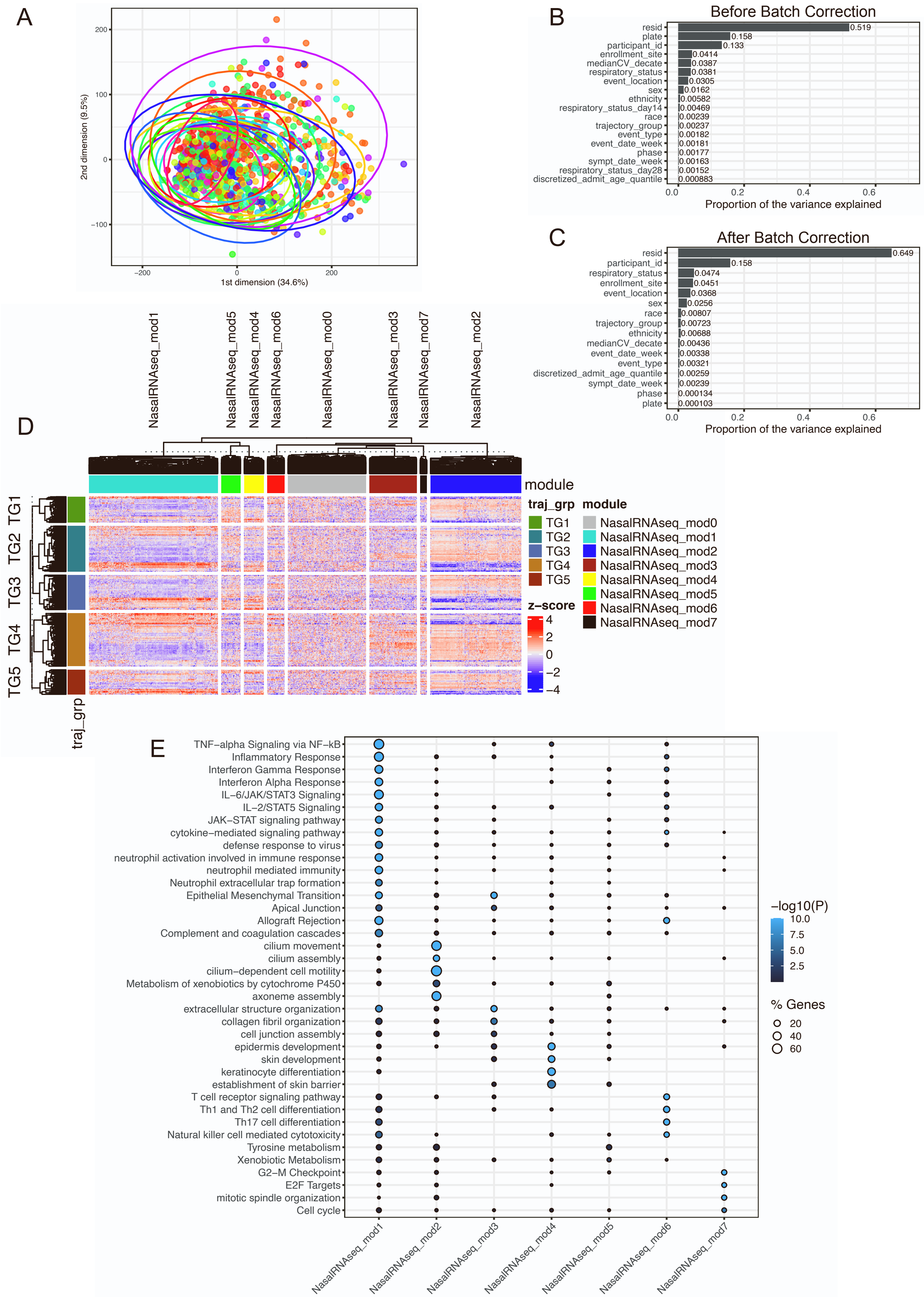

Figure S12. Nasal transcriptomics. Related to Figure 6. (A) The distribution of samples on the first two principal components (PC1, PC2) and samples are colored by enrollment site, and ellipses denote 95% of sample distribution from each enrollment site. (B) PVCA before batch correction depicting the amount of variance explained by various technical and clinical variables. (C) Barplot depicting the amount of variance explained by various technical and clinical variables from principal component variance analysis.(D) Heatmap showing the overall module's expression across all samples. Hierarchical clustering was used to groups samples (rows) and genes (columns). (E) Dot/Bubble plot showing top enriched terms for each module, size of dot denotes percent of genes in the enriched term, and color denotes statistical significance for enrichment.

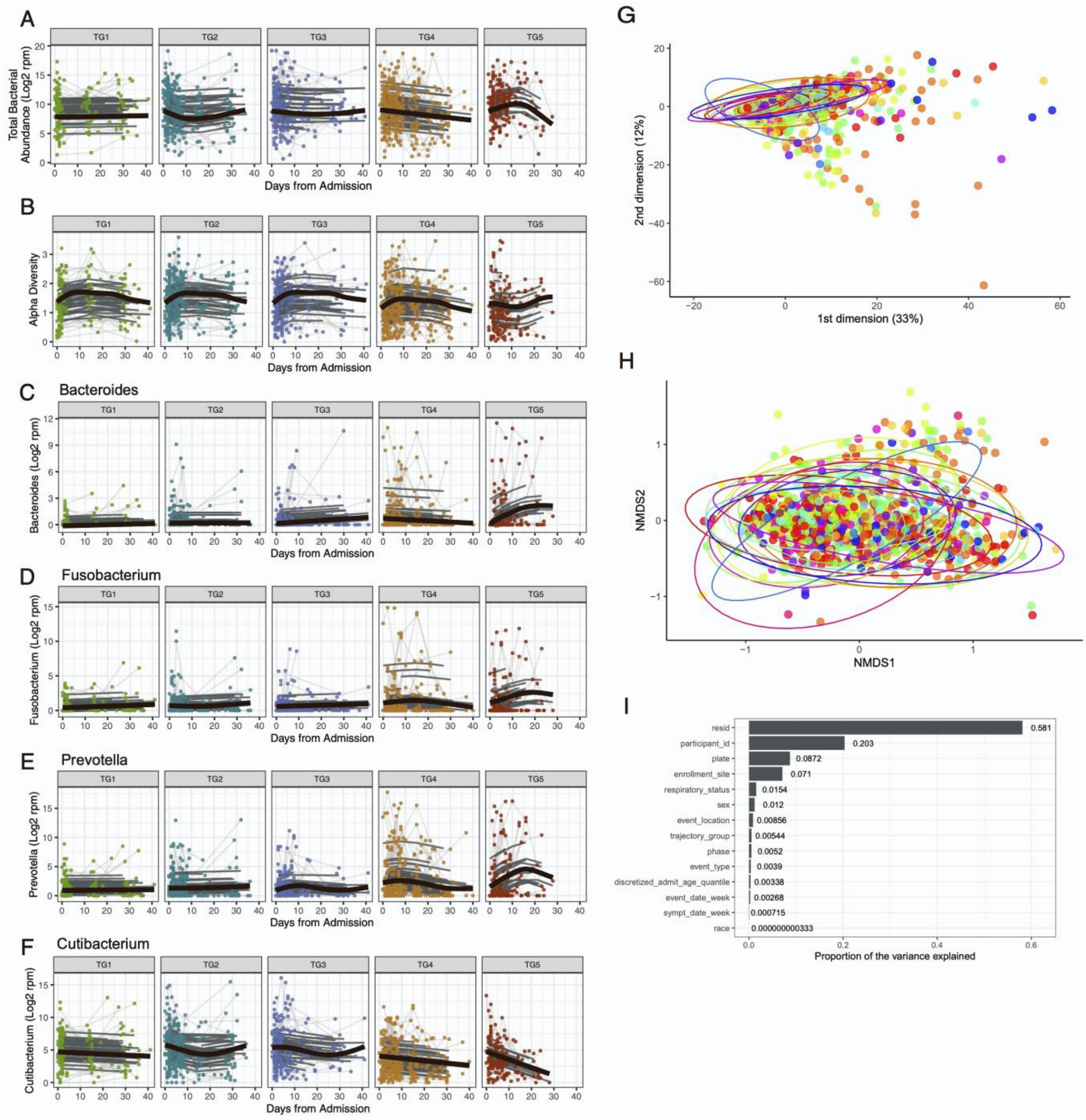

Figure S13: Nasal Metagenomics. Related to Figure 6. (A-B) Total bacterial abundance and microbial alpha diversity demonstrate no significant differences between clinical trajectory groups. Longitudinal modeling of nasal microbial genera between trajectory groups for (C) Bacteroides (shape adj.p = 0.038, average adj.p = 5.4e-4), (D) Fusobacterium (shape adj.p = 0.25, average adj.p = 0.0013), (E) Prevotella (shape adj.p =0.0501, average adj.p = 5.4e-4), and (F) Cutibacterium (shape adj.p = 0.16, average adj.p = 1.7e-5) (G) Principal components 1 and 2 are colored by enrollment site with 95% confidence ellipses demonstrating sample distribution.(H) Non-metric dimensional scaling based on Bray-Curtis Dissimilarity colored by enrollment site with 95% confidence ellipses demonstrating sample distribution. (I) Percent variation is explained by technical variables calculated by principal variance component analysis.
