# Supplementary material for: Multi-omic longitudinal study reveals immune correlates of clinical course among hospitalized COVID-19 patients
Source: Cell Rep Med. 2023 May 23;4(6):101079. doi: 10.1016/j.xcrm.2023.101079 (PMC10203880; doi:10.1016/j.xcrm.2023.101079)
Supplement: Document S2. Supplemental author information [file mmc16.docx]

**Supplementary Author File**

**^#^The IMPACC Network**

**National Institute of Allergy and Infectious Diseases, National Institute of Health, Bethesda, MD 20814, USA:** Patrice M. Becker, Alison D. Augustine**,** Steven M. Holland**,** Lindsey B. Rosen**,** Serena Lee**,** Tatyana Vaysman

**Clinical and Data Coordinating Center (CDCC) Precision Vaccines Program, Boston Children’s Hospital, Boston, MA 02115, USA:** Al Ozonoff, Joann Diray-Arce, Jing Chen, Alvin Kho, Carly E. Milliren, Annmarie Hoch, Ana C. Chang, Kerry McEnaney, Brenda Barton, Claudia Lentucci, Maimouna Murphy, Mehmet Saluvan, Tanzia Shaheen, Shanshan Liu, Caitlin Syphurs, Marisa Albert, Arash Nemati Hayati, Robert Bryant, James Abraham

**Benaroya Research Institute, University of Washington, Seattle, WA 98101, USA:** Matthew C. Altman, Naresh Doni Jayavelu, Scott Presnell, Bernard Kohr, Azlann Arnett

**La Jolla Institute for Immunology, La Jolla, CA 92037, USA:** Bjoern Peters, Randi Vita, Kerstin Westendorf

**Knocean Inc. Toronto, ON M6P 2T3, Canada:** James A. Overton

**Precision Vaccines Program, Boston Children’s Hospital, Harvard Medical School, Boston, MA 02115, USA:** Ofer Levy, Hanno Steen, Patrick van Zalm, Benoit Fatou, Kinga Smolen, Arthur Viode, Simon van Haren, Meenakshi Jha, Boryana Petrova, Naama Kanarek

**Brigham and Women’s Hospital, Harvard Medical School, Boston, MA 02115, USA:** Lindsey R. Baden, Kevin Mendez, Jessica Lasky-Su, Alexandra Tong, Rebecca Rooks

**Metabolon Inc, Morrisville, NC 27560, USA:** Scott R. Hutton, Gregory A. Michelotti, Kari Wong

**Case Western Reserve University and University Hospitals of Cleveland, Cleveland, OH 44106, USA:** Rafick-Pierre Sekaly, Slim Fourati, Grace A. McComsey, Paul Harris, Scott Sieg, Susan Pereira Ribeiro

**Drexel University, Tower Health Hospital, Philadelphia, PA 19104, USA:** Charles B. Cairns, Elias K. Haddad, Michele A. Kutzler, Mariana Bernui, Gina Cusimano, Jennifer Connors, Kyra Woloszczuk, David Joyner, Carolyn Edwards, Edward Lin, Nataliya Melnyk, Debra L. Powell, James N. Kim, I. Michael Goonewardene, Brent Simmons, Cecilia M. Smith, Mark Martens, Brett Croen, Nicholas C. Semenza, Mathew R. Bell, Sara Furukawa, Renee McLin, George P Tegos, Brandon Rogowski, Nathan Mege, Kristen Ulring

**MyOwnMed Inc., Bethesda, MD 20817, USA:** Vicki Seyfert-Margolis

**Emory School of Medicine, Atlanta, GA 30322, USA:** Nadine Rouphael, Steven E. Bosinger, Arun K. Boddapati, Greg K. Tharp, Kathryn L. Pellegrini, Brandi Johnson, Bernadine Panganiban, Christopher Huerta, Evan J. Anderson, Hady Samaha, Jonathan Sevransky, Laurel Bristow, Elizabeth Beagle, David Cowan, Sydney Hamilton, Thomas Hodder

**Icahn School of Medicine at Mount Sinai, New York, NY 10029, USA:** Ana Fernandez-Sesma, Viviana Simon, Florian Krammer, Harm Van Bakel, Seunghee Kim-schulze, Ana Silvia Gonzalez-Reiche, Jingjing Qi, Brian Lee, Juan Manuel Carreño, Gagandeep Singh, Ariel Raskin, Johnstone Tcheou, Zain Khalil, Adriana van de Guchte, Keith Farrugia, Zenab Khan, Geoffrey Kelly, Komal Srivastava, Lily Eaker, Maria Carolina Bermúdez González, Lubbertus C.F. Mulder, Katherine Beach

**Immunai Inc. New York, NY 10016, USA:** Adeeb Rahman

**Oregon Health Sciences University, Portland, OR 97239, USA:** William B. Messer, Catherine L. Hough, Sarah Siegel, Peter Sullivan, Zhengchun Lu

**Stanford University School of Medicine, Palo Alto, CA 94305, USA:** Holden Maecker, Bali Pulendran, R. Kari C. Nadeau, Yael Rosenberg-Hasson, Michael Leipold, Natalia Sigal, Angela Rogers, Andrea Fernandez, Monali Manohar, Evan Do, Iris Chang

**David Geffen School of Medicine at the University of California Los Angeles, Los Angeles CA 90095, USA:** Elaine F. Reed, Joanna Schaenman, Ramin Salehi-Rad, Adreanne M. Rivera, Harry C. Pickering, Subha Sen, David Elashoff, Dawn C. Ward

**University of California San Francisco, San Francisco, CA 94115, USA:** David J. Erle, Carolyn S. Calfee, Carolyn M. Hendrickson, Kirsten N. Kangelaris, Viet Nguyen, Deanna Lee, Suzanna Chak, Rajani Ghale, Ana Gonzalez, Alejandra Jauregui, Carolyn Leroux, Luz Torres Altamirano, Ahmad Sadeed Rashid, Andrew Willmore, Prescott G. Woodruff, Matthew F. Krummel, Sidney Carrillo, Alyssa Ward, Charles R. Langelier, Ravi Patel, Michael Wilson, Ravi Dandekar, Bonny Alvarenga, Jayant Rajan, Walter Eckalbar, Andrew W. Schroeder, Gabriela K. Fragiadakis, Alexandra Tsitsiklis, Eran Mick, Yanedth Sanchez Guerrero, Rajani Ghale, Christina Love, Lenka Maliskova, Michael Adkisson

**Yale School of Medicine, New Haven, CT 06510, USA:** David A. Hafler, Ruth R. Montgomery, Albert C. Shaw, Steven H. Kleinstein, Jeremy Gygi, Shrikant Pawar, Anna Konstorum, Ernie Chen, Chris Cotsapas, Xiaomei Wang, Leqi Xu, Charles Dela Cruz, Akiko Iwasaki, Subhasis Mohanty, Allison Nelson, Yujiao Zhao, Shelli Farhadian, Hiromitsu Asashima

**Yale School of Public Health, New Haven, CT 06510, USA:** Denise Esserman, Leying Guan, Anderson Brito, Jessica Rothman, Nathan Grubaugh, Albert I. Ko

**Baylor College of Medicine and the Center for Translational Research on Inflammatory Diseases, Houston, TX 77030, USA:** David B. Corry, Farrah Kheradmand, Li-Zhen Song, Ebony Nelson

**Oklahoma University Health Sciences Center, Oklahoma City, OK 73104, USA:** Jordan P. Metcalf, Nelson I Agudelo Higuita, Lauren Sinko, J. Leland Booth

**University of Arizona, Tucson AZ 85721, USA:** Monica Kraft, Chris Bime, Jarrod Mosier, Heidi Erickson, Ron Schunk, Hiroki Kimura, Michelle Conway

**University of Florida, Gainesville, FL 32611, USA:** Mark A. Atkinson, Scott C. Brakenridge, Ricardo F. Ungaro, Brittany Roth Manning,

**University of Florida, Jacksonville, FL 32218, USA:** Jordan Oberhaus, Faheem W. Guirgis,

**University of South Florida, Tampa FL 33620, USA:** Brittney Borresen, Matthew L. Anderson

**University of Texas, Austin, TX 78712, USA:** Lauren I. R. Ehrlich, Esther Melamed, Cole Maguire, Justin F. Rousseau, Kerin C. Hurley, Janelle N. Geltman, Nadia Siles, Jacob E. Rogers
